# Using automated synthesis to understand the role of side chains on molecular charge transport

**DOI:** 10.1038/s41467-022-29796-2

**Published:** 2022-04-19

**Authors:** Songsong Li, Edward R. Jira, Nicholas H. Angello, Jialing Li, Hao Yu, Jeffrey S. Moore, Ying Diao, Martin D. Burke, Charles M. Schroeder

**Affiliations:** 1 Department of Materials Science and Engineering, University of Illinois at Urbana-Champaign, Urbana, IL 61801 United States; 2 Beckman Institute for Advanced Science and Technology, University of Illinois at Urbana-Champaign, Urbana, IL 61801 United States; 3 Department of Chemical and Biomolecular Engineering, University of Illinois at Urbana-Champaign, Urbana, IL 61801 United States; 4 Department of Chemistry, University of Illinois at Urbana-Champaign, Urbana, IL 61801 United States; 5 Carle Illinois College of Medicine, University of Illinois at Urbana-Champaign, Urbana, IL 61801 United States

**Keywords:** Automation, Chemical physics, Molecular electronics

## Abstract

The development of next-generation organic electronic materials critically relies on understanding structure-function relationships in conjugated polymers. However, unlocking the full potential of organic materials requires access to their vast chemical space while efficiently managing the large synthetic workload to survey new materials. In this work, we use automated synthesis to prepare a library of conjugated oligomers with systematically varied side chain composition followed by single-molecule characterization of charge transport. Our results show that molecular junctions with long alkyl side chains exhibit a concentration-dependent bimodal conductance with an unexpectedly high conductance state that arises due to surface adsorption and backbone planarization, which is supported by a series of control experiments using asymmetric, planarized, and sterically hindered molecules. Density functional theory simulations and experiments using different anchors and alkoxy side chains highlight the role of side chain chemistry on charge transport. Overall, this work opens new avenues for using automated synthesis for the development and understanding of organic electronic materials.

## Abstract (web summary)

Development of organic electronic materials relies on understanding structure-function relationships in conjugated polymers but the synthetic workload to make large numbers of new compounds presents a practical barrier to properly survey conjugated organic derivatives. Here, the authors use automated synthesis to prepare a library of conjugated oligomers with systematically varied side chain composition followed by single-molecule characterization of charge transport.

## Introduction

The development of high-performance organic electronic devices critically relies on a fundamental understanding of intra- and intermolecular charge transport^1,2^. Organic electronic materials are typically designed for high charge-carrier mobilities and generally have highly delocalized conjugated backbones, with common building blocks including acenes, fluorenes, phenylene vinylene derivatives, thiophene derivatives, diketopyrrolopyrrole, isoindigo, and their donor-acceptor copolymers^1,2^. Strong pi–pi interactions in these systems significantly decrease their solubility in organic solvents, which hinders solution processing. To overcome this issue, flexible side chains such as alkyl, fluoroalkyl, and oligo(ethylene glycol) chains are appended to pi-conjugated backbones to enhance solubility, facilitate solution processing, and tune intermolecular packing^3,4^. In recent years, side chain engineering has been pursued organic electronic materials, which highlights the importance of side chain length, bulkiness, branching, substitution, and chain-end functionalization on charge transport in conjugated polymers^3,4^. However, the majority of prior work has focused on understanding the role of backbone composition and side chain chemistry on the macroscopic or device-level properties of these materials. Despite recent progress, the role of side chain chemistry on the molecular charge transport properties of conjugated organics is not fully understood.

Unlocking the full potential of organic materials relies on accessing their vast chemical space, but the synthetic workload to make large numbers of new compounds presents a practical barrier to properly survey conjugated organic derivatives. In recent years, automated iterative coupling has emerged as a promising avenue to achieve precise sequence control and enable high-throughput synthesis of oligomeric small molecules^5^. Recent efforts have leveraged general Suzuki cross-coupling reactions using stable and accessible chemical building blocks in the development of an automated small-molecule synthesizer^6^. In this way, automated synthesis platforms have emerged as powerful tools to advance our understanding of functional materials properties, but these methods have not yet been widely applied to the field of organic electronics. In general, we lack a full systematic understanding of structure-property relationships for organic electronic materials due to the tedious and challenging nature of chemical synthesis.

In this work, we systematically investigate the role of side chain chemistry on the charge transport properties of conjugated oligomers using a combination of automated synthesis and single-molecule charge transport experiments. A library of terphenyl derivatives with different side chain compositions and anchors was synthesized using automated iterative Suzuki coupling. Following synthesis and chemical characterization, a scanning tunneling microscope-break junction (STM-BJ) technique was used to directly characterize the charge transport properties of these molecules. STM-BJ methods provide an ideal approach for understanding charge transport at the molecular level, thereby enabling quantitative structure-property relationships for organic materials^7–9^. Our results show that molecular junctions with long alkyl side chains exhibit a bimodal conductance distribution with an unexpectedly high conductance state upon increasing concentration. Systematic control experiments using asymmetric, planarized, and sterically encumbered molecules indicate that the high conductance state results from surface adsorption and molecular planarization facilitated by long alkyl side chains. Our results further show that the choice of chemical anchors and side chains directly affects molecular adsorption, and density functional theory (DFT) simulations are in reasonable agreement with experiments. Taken together, these results highlight the use of automated chemical synthesis to/understand molecular charge transport for the development of new organic electronic materials.

## Results and discussion

### Automated synthesis of terphenyl derivatives with varying alkyl side chains

**Fig. 1:**
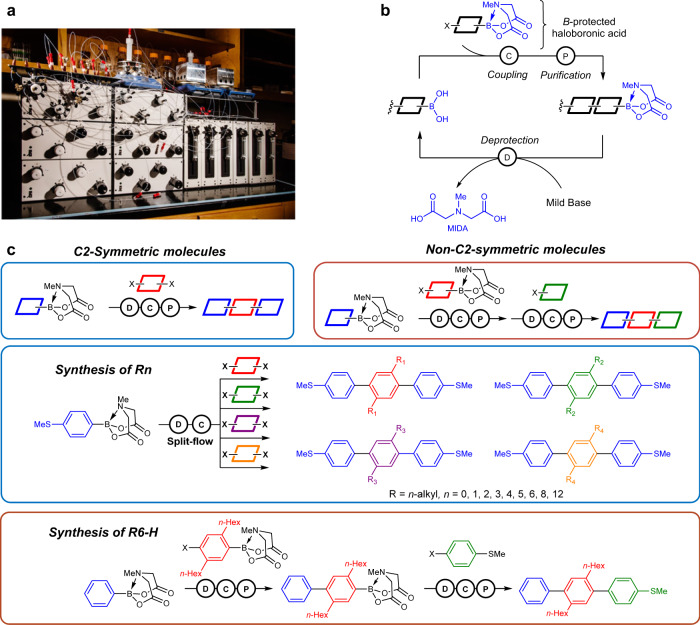
Automated chemical synthesis of terphenyl derivatives with different side chain compositions. **a** Picture of the automated synthesis instrument in our lab. **b** Iterative coupling strategy for small-molecule synthesis using MIDA boronates. **c** Synthesis schemes for C2-symmetric and non-C2-symmetric terphenyl derivatives via iterative Suzuki coupling.

We used automated iterative Suzuki-Miyaura cross-coupling to prepare a library of terphenyl derivatives using stable and easily accessible molecular building blocks containing methyliminodiacetic acid (MIDA) boronates and dihalide groups (Supplementary Information, Sections S.1 and S.2 and Supplementary Figs. S16–S81). Building blocks contained phenyl rings with pre-installed alkyl side chains and terminal anchor moieties to facilitate STM-BJ experiments. Building on the first-generation automated small-molecule synthesizer designed by Burke and coworkers^6^, we developed a second-generation small-molecule synthesizer capable of parallel runs of deprotections, couplings, and purifications (Fig. 1a). Briefly, the synthesis strategy relies on the MIDA protecting group rendering boron unreactive until deprotected with mild aqueous base, analogous to the Fmoc group in iterative peptide synthesis (Fig. 1b). The new automated synthesis instrument leverages advances in hardware and software to enable up to 12 fully parallelized, simultaneous preparative-scale iterative Suzuki reactions. Importantly, the increased throughput allows for the screening of large regions of chemical space in a single automated synthetic run. Using this approach, a library of symmetric terphenyl derivatives with different alkyl side chains (**Rn**) and an asymmetric target molecule (**R6-H**) were prepared using two automated procedures in conjunction with the standard building block set (Fig. 1c).

### Role of alkyl side chain length on molecular charge transport

**Fig. 2:**
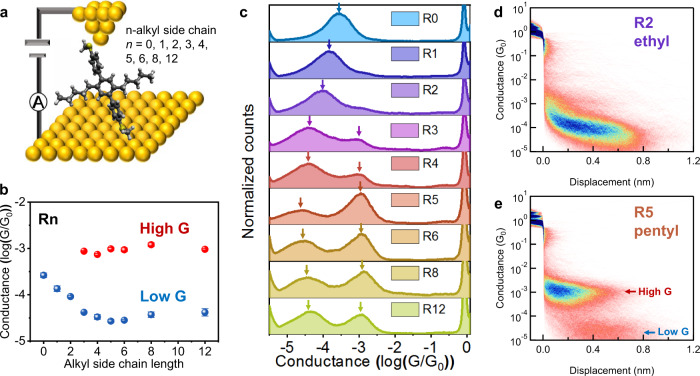
Single-molecule characterization of charge transport in terphenyl derivatives with different alkyl side chains. **a** Schematic of Au–Rn–Au junction. **b** Conductance peak values of 1 mM Rn at 0.25 V applied bias. **c** 1D conductance histograms for 1 mM Rn at 0.25 V bias voltages, each constructed from >4000 traces. **d**, **e** Representative 2D conductance histograms for R2 and R5.

We began by studying the molecular conductance properties of **Rn** (Fig. 2a) using a custom-built scanning tunneling microscope-break junction (STM-BJ) instrument, as previously described^10,11^. STM-BJ has been used in prior work to study single-molecule charge transport as a function of backbone length^10,12^, chemical substituents^13,14^, molecular conformation^15,16^, and anchor-electrode contacts^17^. Prior work has shown that alkyl side chains alter backbone conformation^16^, thereby affecting molecular conductance. Here, we used STM-BJ to systematically understand the role of side chain length and composition in the molecular library generated by automated synthesis. Using this approach, we determined the molecular conductance of **Rn** in a nonpolar solvent (1 mM solution in 1,2,4-trichlorobenzene) at 0.25 V applied bias. One-dimensional (1D) (Fig. 2b, c) and two-dimensional (2D) (Fig. 2d, e and Supplementary Fig. 1) molecular conductance histograms were determined for **Rn**, such that each histogram is generated from a large ensemble of >4000 individual traces. Interestingly, molecular/conductance in these terphenyl derivatives shows an unexpected dependence on alkyl chain length. In particular, **R0-R2** only shows a single prominent conductance peak, and molecular conductance decreases upon increasing the alkyl chain length (Fig. 2b). The average molecular displacement for **R0-R2** is constant (≈0.8 nm) because the alkyl side chain does not significantly affect molecular end-to-end distance for terphenyl derivatives with short side chains (Fig. 2d and Supplementary Fig. 1). In contrast, **R3-R12** exhibits two dominant and well-spaced conductance states (high G and low G). Interestingly, the conductance of the high G state (10^−3^ *G*_*0*_) is more than one order of magnitude larger than the low G state (≈10^−4^–10^−5^ *G*_*0*_). The molecular displacement corresponding to the high G state is ~0.4 nm, and the molecular conductance of the high G state is independent of the alkyl side chain length for **R3-R12** (Fig. 2b and Supplementary Fig. 1).

**Fig. 3:**
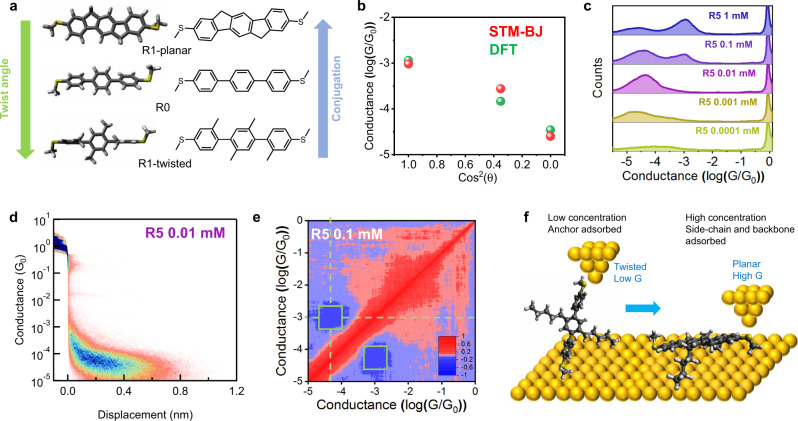
Molecular origin of the high conductance state. **a** Terphenyl derivatives with different conformations. **b** Peak molecular conductance values for terphenyl derivatives with different dihedral angles from DFT simulations and experiments. Dihedral angles are determined from the lowest energy conformers using DFT simulations. **c** 1D molecular conductance histograms showing the concentration-dependent behavior of R5. **d** Representative 2D conductance histograms of R5 (0.01 mM) showing only the low G state. **e** 2D correlation of molecular conductance for R5 (0.1 mM). **f** Schematic showing mechanism for concentration-dependent conformation and conductance behavior of single-molecule junctions. The high G and low G states both arise due to transport through the long axis of the molecule, facilitated by the linkage of the molecule to the tip and substrate between two terminal anchors.

Alkyl side chains are known to change the twist angle between planar conjugated units in molecular backbones, which affects electronic properties^15,16^. To understand the role of backbone conformation on molecular conductance, we performed molecular modeling of conductance in terphenyl junctions with varying twist angles. Terphenyl junctions with different conformations (Fig. 3a) were modeled using DFT calculations performed on Spartan’16 Parallel Suite using the B3LYP functional with a 6–31 G (d,p) basis set. Following the determination of geometry-optimized structures, transmission functions were calculated using nonequilibrium Green’s function-density functional theory (NEGF-DFT) via the Atomistix Toolkit package (Fig. 3b and Supplementary Figs. 2, 3). Our results show that the transmission values close to the Fermi energy decrease with *cos*^*2*^ *θ* (between *θ* = 0^o^ and 90^o^*)*, where *θ* is the dihedral angle between the first two phenyls. The simulated conductance is ≈10^−2.9^ *G*_*0*_ for terphenyl junctions with full planar conformations and ≈10^−4.5^ *G*_*0*_ when the dihedral angle is close to 90^o^, which is in good agreement with the experimentally observed high G and low G states, respectively (Fig. 2b). To further validate these results, we synthesized two control molecules with planar (**R1-planar**) and twisted (**R1-twisted**) conformations (Fig. 3a). The experimentally measured conductance values for these molecules are in reasonable agreement with results from NEGF-DFT simulations (Fig. 3b and Supplementary Fig. 4). These results suggest that the low G state derives from twisted conformations and depends on backbone dihedral angles, whereas the high G state is likely associated with a planarized backbone but is only observed for long alkyl side chains (*n* ≥ 3). However, the molecular displacement corresponding to the high G state is ≈0.4 nm, which is significantly smaller than the expected displacement of an extended molecular backbone for a terphenyl derivative.

To fully understand the molecular origin of the high G state, we performed a series of single-molecule conductance experiments using **R3-R12** at different concentrations (100 nM–1 mM) (Fig. 3c and Supplementary Fig. 5). Surprisingly, our results show that the emergence of the high conductance state depends on the solution concentration of **R3-R12**. **R3** exhibits a single peak (low G state) at relatively low concentrations (<1 mM) and dual peaks (high G and low G states) at high concentrations (1 mM). Interestingly, the onset concentration for the emergence of the high G state decreases from 1 to 0.1 mM upon increasing the length of the alkyl side chain (Supplementary Fig. 5). Moreover, **R3-R12** do not exhibit dual peaks at very low concentrations at 0.01 mM (Fig. 3d and Supplementary Fig. 6), whereas **R2** and **R3-iPr** (containing the isomeric isopropyl side chain) do not exhibit a high G state at very high concentrations (10 mM) (Supplementary Fig. 7), suggesting that a minimum linear alkyl side chain length is required for the emergence of the high conductance state. In addition, we analyzed correlations between the high G and low G states for **R3-R12** by determining a 2D covariance histogram^18–20^ (Fig. 3e and Supplementary Fig. 8). In a 2D covariance histogram, correlations are used to determine whether two conductance states occur independently (negative correlation, shown in blue) or sequentially (positive correlation, shown in red) in a single-molecule conductance trace. These results show a clear negative correlation between the high G and low G states (two blue regions in the green boxes), indicating that these two conductance states occur independently and therefore do not occur sequentially in a single-molecule trace.

Recent work has reported that additional conductance states may arise from in-backbone molecule-electrode linkages^9,21–24^, in situ dimerization^17,25–27^, or intermolecular interactions^28–30^. To investigate the origin of the high G state, we synthesized the non-C2-symmetric terphenyl derivative **R6-H** containing only one terminal anchor using automated iterative cross-coupling (Fig. 1c). Single-molecule characterization using STM-BJ shows that **R6-H** cannot form stable molecular junctions and therefore does not exhibit a high G state (10^−3^ *G*_*0*_) at 0.1 mM (Supplementary Fig. 9). These results suggest that the high G state does not arise from intermolecular interactions or in-backbone linkages between the molecule and gold electrodes. To investigate the potential for solution-based aggregation, we performed an ^1^H NMR and UV-vis dilution experiments at experimentally relevant concentrations for **R6** (Supplementary Information, Section S.4 and Supplementary Figs. 13, 14). Overall, these experiments showed no significant spectral changes for concentrations between 0.01 and 10 mM, which further suggests that solution-based molecular aggregation is not responsible for the high G state.

We further performed flicker noise analysis experiments for **R6** at a solution concentration of 1 mM. Flicker noise analysis has been used in prior work to understand the nature of electronic coupling at metal-molecule interfaces^29,31,32^. In this way, through-bond transport (where flicker noise scales as *G*^*1.0*^) is distinguished from through-space transport (where flicker noise scales as *G*^*2.0*^) based on the power-law exponent of “noise power” versus average conductance, where noise power is determined from power spectral density analysis of conductance fluctuations^13^. Intermolecular charge transport typically results in larger fluctuations in conductance, which corresponds to through-space distributions of flicker noise^29^. Our results show that the noise power scales as *G*^*1.01*^ for the high G and *G*^*1.36*^ for the low G state, corresponding to through-bond coupling in molecular junctions (Supplementary Fig. 10). These results further exclude the possibility of intermolecular interactions such as molecular aggregation, which is consistent with ^1^H NMR dilution experiments. Taken together, the combination of flicker noise analysis and control experiments indicates that the high G state arises from molecule-electrode linkages between SMe anchors at both termini.

Based on these results, we hypothesized that long alkyl side chains (*n* ≥ 3) promote adsorption of the terphenyl backbone onto gold surfaces through van der Waals interactions, thereby inducing backbone conformational changes leading to increasingly planar backbone geometries (Fig. 3f). To test this hypothesis, we developed an analytical model based on Langmuir adsorption with a weakly bound conformation (low G state) that converts to a strongly bound state (high G state) (Supplementary Information, Section S.5). Here, the low G state corresponds to an upright molecular conformation with a junction anchored at both termini to metal electrodes, whereas the high G state corresponds to a molecular conformation in which a molecule is “lying down” on the electrode surface via side chain-mediated van der Waals interactions. The concentration-dependent conductance behavior of the high G state suggests that molecular junctions transition from a “standing up” to a “lying down” conformation facilitated by intermolecular interactions between free molecules in solution and molecules linked to the surface electrode via a terminal anchor^33^, thereby planarizing the terphenyl backbone and reducing steric hindrance for the adsorbed molecule to lie down on the surface. The sequential two-state adsorption results in a threshold-level dependence of solution concentration for the “lying down” conformation (Supplementary Fig. 15). As solution concentration is increased, the “lying down” confirmation becomes increasingly dominant, and the molecular sub-population exhibiting the high G state conductance increases. Overall, the results from the analytical model qualitatively agree with the concentration-dependent conductance behavior observed in experiments.

### Molecular adsorption behavior in amine-terminated terphenyl derivatives

**Fig. 4:**
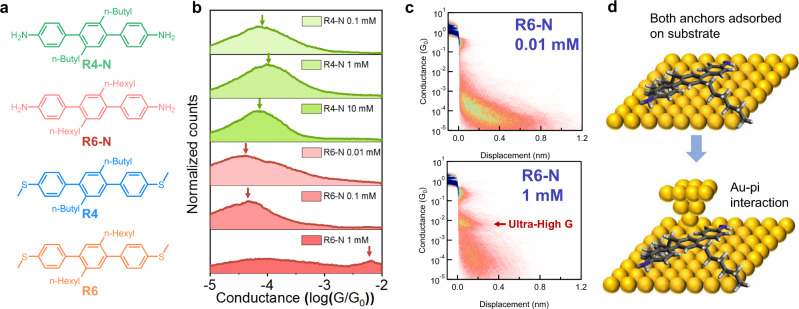
Effect of alkyl side chains in amine-terminated terphenyl derivatives. **a** Chemical structures of R4-N, R6-N, R4, and R6. **b** Concentration-dependent study of R4-N and R6-N. **c** 2D conductance histograms of 0.01 mM (low G state) and 1 mM (ultra-high G state) R5. **d** Hexyl side chains in R6-N facilitate backbone adsorption and planarization. However, the gold tip cannot pick up from the amine end, which induces Au–pi interaction and exhibit ultra-high conductance.

To further understand the role of terminal anchor groups on the conductance behavior of terphenyl derivatives, we synthesized two amine-terminated terphenyls with different alkyl side chain lengths (**R4-N** and **R6-N**) using our automated synthesis method (Fig. 4a). It is known that SMe and NH_2_ serve as dative coordination anchors for Au electrodes, yet exhibit different contact resistance, binding behavior, and adsorption free energies in single-molecule experiments^34–36^. Interestingly, our results show that amine-terminated terphenyls exhibit different concentration-dependent conductance behaviors compared to their SMe-terminated counterparts. **R4-N** does not show a high G state even at relatively high concentrations (10 mM), suggesting a lack of side chain-mediated molecular adsorption for **R4-N** on electrode surfaces (Fig. 4b) despite containing identical alkyl chains as **R4**. We posit that this phenomenon arises from the low adsorption free energy and different binding behavior of NH_2_ compared to SMe anchors, which in turn inhibits backbone adsorption of **R4-N** at concentrations up to 10 mM. On the other hand, **R6-N** shows the emergence of an ultra-high conductance state (10^−2^ *G*_*0*_) at 1 mM concentration (Fig. 4b, c), which contrasts with the behavior of **R6** and **R4-N**. The ultra-high conductance state of **R6-N** occurs over small molecular displacements and likely arises due to perpendicular transport via Au–pi–orbital interactions (Fig. 4d), as reported in prior work^37,38^. If two-terminal amine anchors are involved in surface binding, the probability of junction formation is likely reduced due to an inability of the tip to disrupt molecule-surface binding interactions. Terminal amine anchors only contain a single lone electron pair instead of two lone pairs for SMe anchors, which we speculate may decrease the availability for dative interactions between a lone electron pair and the free orbital on the gold tip. This mechanism of transport is consistent with the conductance pattern of **R6-H** (containing a single terminal SMe anchor) at 1 mM concentration (Supplementary Fig. 9), which exhibits a similar ultra-high conductance state (10^−2^ *G*_*0*_) as **R6-N**.

### Role of side chain chemistry on molecular charge transport

**Fig. 5:**
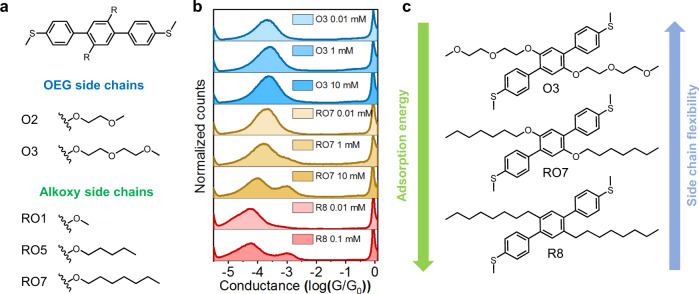
Single-molecule characterizations of terphenyl derivatives with OEG and alkoxy chains. **a** Chemical structures of terphenyl derivatives with OEG and alkoxy chains. **b** Concentration-dependent study of O3, RO7, and R8. **c** Differences of flexibility and adsorption free energy between O3, RO7, and R8.

Oligo(ethylene glycol) (OEG) and alkoxy chains contain oxygen atoms that increase hydrophilicity, polarity, and chain flexibility compared to hydrophobic alkyl side chains^39^. Conjugated materials bearing OEG or alkoxy side chains exhibit different morphologies and electronic properties compared to their alkyl side chains counterparts^39–42^. To investigate the role of side chain chemistry and composition on molecular charge transport, we synthesized a series of terphenyl derivatives with OEG and alkoxy side chains using automated synthesis (Fig. 5a). In general, molecules with OEG/alkoxy chains show higher conductance and reduced molecular/adsorption behavior compared to their alkyl side chain counterparts (Fig. 5b and Supplementary Figs. 11, 12). Enhanced intramolecular conductance for molecules with OEG/alkoxy chains likely derives from two aspects. First, oxygen atoms in the side chain generally increase HOMO energy levels, which results in slightly better alignment with the Fermi energy level of Au electrodes^14^ (Supplementary Table 1). Second, molecules with oxygen-containing side chains generally show more planar backbone conformations^43^, which enhances charge transport (Supplementary Table 1). We further hypothesize that the reduced adsorption behavior for molecules with OEG/alkoxy chains is related to side chain flexibility. Prior work reported that introducing oxygen atoms into linear alkyl chains decreases the barriers to rotation around the corresponding single bonds, thereby increasing degrees of freedom for the linear chains^39^. More flexible oxygen-containing side chains may incur a greater entropic cost upon surface immobilization. The increased protein binding affinity of macrocyclic small molecules relative to their linear counterparts is attributed to a similar phenomenon^44,45^. To test this hypothesis, we synthesized a series of molecules with the same side chain length but varying levels of side chain oxygenation (**O3**, **RO7**, and **R8**). In the case of **RO7** (side chains with a single oxygen atom), the high conductance peak appears prominently at 10 mM compared to 0.1 mM for **R8** (Fig. 5b). On the other hand, **O3** (containing three oxygen atoms) did not show a prominent high conductance peak for concentrations up to 10 mM (Fig. 5b and Supplementary Fig. 12). These results are consistent with increased oxygenation of side chains increasing chain flexibility and suppressing side chain-mediated adsorption (Fig. 5c). Moreover, it is possible that aliphatic chains form stronger van der Waals interactions with the electrode surface than their oxygenated counterparts^46^.

In this work, we use automated synthesis and single-molecule experiments to investigate the effect of side chain chemistry on the charge transport properties of terphenyl derivatives. Broadly, these results deepen our understanding of structure–function relationships in organic electronic materials and highlight the need to fully understand the effect of substitution position, side chain chemistry, backbone identity, and side chain functionalization on charge transport. Overall, our results show that molecular adsorption and molecular conformation can be controlled using different side chain chemistries and anchor groups, which is useful for informing complementary studies on side chain engineering involving organic electronic devices and thin films. However, our work focuses primarily on intrachain transport which complicates direct comparison to studies involving organic thin films due to the combined roles of intra- and intermolecular transport in those systems. Nevertheless, our results provide new strategies for interface engineering to tune the device performance of organic electronics. Interface engineering has been widely used to tune the device performance of organic electronics^47^. Adsorbed molecules at organic electronics interfaces affect charge injection barriers and change thin film morphologies^47–49^. From this view, our work provides molecular engineering strategies to control the molecular adsorption and conformation at semiconductor-electrode interfaces using different side chain chemistries and anchors. For example, our work shows that the amine-terminated **R6-N** molecule exhibits “standing up” and “lying down” conformations at different concentrations. Here, the interfacial surface energy is expected to similarly change with concentration as well. Broadly, our work demonstrates the utility of automated chemical synthesis to enable efficient and systematic exploration of chemical space for organic electronic materials. Overall, this work opens new avenues in combining automated synthesis with single-molecule characterization to aid in the design of new materials for organic electronics.

## Methods

### Small-molecule synthesis

Automated small-molecule synthesis was performed in parallel on a Burke-type small-molecule synthesizer^6^ using two general automated procedures (iterative or non-iterative Suzuki coupling). Building blocks (MIDA boronates, halides), palladium catalyst, and inorganic base were loaded onto the synthesizer along with cartridges for drying, precipitation, and purification, followed by execution of the automated synthesis procedures. After completion of the automated procedures, crude reaction mixtures were purified using medium pressure liquid chromatography (MPLC) or preparative high-performance liquid chromatography (HPLC). These procedures are generally amenable to scale-up and can be performed by hand to achieve similar results at a slower pace. For full experimental details and chemical characterization, see the Supplementary Information.

### Single-molecule conductance measurements

Single-molecule conductance measurements were performed using a home-built scanning tunneling microscope setup^9,15^. Gold STM tips were prepared using 0.25 mm Au wire (99.998%, Alfa Aesar). Gold substrates were prepared by evaporating 120 nm of gold onto polished AFM metal specimen disks (Ted Pella). Conductance measurements were carried out in 0.001–1 mM molecule solution in 1,2,4-trichlorobenzene. Break junction experiments were performed at a constant bias as described. One- and two-dimensional conductance histograms (>4000 traces for each molecule) are constructed without data selection.

### Flicker noise analysis

Flicker noise analysis was performed to distinguish between intramolecular and intermolecular modes of charge transport for a subset of molecules^31^. Conductance fluctuations were measured by holding individual molecular junctions at a fixed position for 150 ms with a sampling rate of 40 kHz after the junctions were formed between the tip and substrate electrode. After each measurement, the junctions were further elongated until ruptured before repeating the measurement. This process was repeated for >15,000 iterations for each sample. During analysis, only traces where junctions survived throughout the entire 150-ms holding period were considered. The discrete Fourier transformation was applied and squared to the conductance data in the holding phase of the experiment to obtain the noise power spectral density (PSD). Flicker noise was quantified by numerically integrating the PSD between frequencies of 100 Hz to 1 kHz, followed by normalizing the average conductance of the corresponding trace. Two-dimensional histograms of normalized noise power (PSD/*G*) versus the average conductance (*G*) were constructed from “effective” traces with junction conductance within one standard deviation from the ensemble peak average conductance. The relationship between the normalized noise power (PSD/*G*^*n*^) and the average conductance (*G*) was determined by 2D Gaussian fitting to the 2D histograms^29^, where *n* is the scaling exponent. The scaling exponent *n* was varied between *n* = 1–2 with a step size of 0.05 to minimize the correlation parameter from the 2D Gaussian fit.

### Molecular modeling and density functional theory (DFT) simulations

Electron transport calculations were performed using the nonequilibrium Green’s function-density functional theory (NEGF-DFT) method via the Atomistix Toolkit package. Molecular geometries are first optimized using Spartan with a 6–31 G** basis. Geometry-optimized molecules are then placed in built junctions, and all atoms are relaxed to 0.05 ev/Å using DFT with local spin density approximation, a double-ζ polarized basis set for the molecules (except for gold atoms which use a single-ζ basis set), and k-point samplings of 3 × 3 × 50, where 50 is the direction of transport. Transmission spectra are then calculated for the junction.

## Supplementary information

Supplementary Information

Supplementary dataset

## Data Availability

All other data are available from the corresponding author upon request.

## Code availability

The STM-BJ data that support the findings were acquired using a custom instrument controlled by custom software (Igor Pro, Wavemetrics). The automated small-molecule synthesizer was controlled by custom software (LabVIEW). The data analysis software is available from the corresponding author upon request.

## References

[1] Wang C, Dong H, Hu W, Liu Y, Zhu D (2012). Semiconducting π-conjugated systems in field-effect transistors: a material odyssey of organic electronics. Chem. Rev..

[2] Mei J, Diao Y, Appleton AL, Fang L, Bao Z (2013). Integrated materials design of organic semiconductors for field-effect transistors. J. Am. Chem. Soc..

[3] Mei J, Bao Z (2014). Side chain engineering in solution-processable conjugated polymers. Chem. Mater..

[4] Lei T, Wang J-Y, Pei J (2014). Roles of flexible chains in organic semiconducting materials. Chem. Mater..

[5] Trobe M, Burke MD (2018). The molecular industrial revolution: automated synthesis of small molecules. Angew. Chem. Int. Ed..

[6] Li J (2015). Synthesis of many different types of organic small molecules using one automated process. Science.

[7] Su TA, Neupane M, Steigerwald ML, Venkataraman L, Nuckolls C (2016). Chemical principles of single-molecule electronics. Nat. Rev. Mater..

[8] Xin N (2019). Concepts in the design and engineering of single-molecule electronic devices. Nat. Rev. Phys..

[9] Yu H (2020). Charge transport in sequence-defined conjugated oligomers. J. Am. Chem. Soc..

[10] Li B (2019). Intrachain charge transport through conjugated donor–acceptor oligomers. ACS Appl. Electron. Mater..

[11] Li S (2019). Charge transport and quantum interference effects in oxazole-terminated conjugated oligomers. J. Am. Chem. Soc..

[12] Venkataraman L (2006). Single-molecule circuits with well-defined molecular conductance. Nano Lett..

[13] Tan, Z. et al. The control of intramolecular through-bond and through-space coupling in single-molecule junctions. *CCS Chem.* **4**, 929–937 (2021).

[14] Li, S. et al. Transition between nonresonant and resonant charge transport in molecular junctions. *Nano Lett.* **21**, 8340–8347 (2021). 10.1021/acs.nanolett.1c02915 34529446

[15] Venkataraman L, Klare JE, Nuckolls C, Hybertsen MS, Steigerwald ML (2006). Dependence of single-molecule junction conductance on molecular conformation. Nature.

[16] Dell EJ (2013). Impact of molecular symmetry on single-molecule conductance. J. Am. Chem. Soc..

[17] Li S (2020). Covalent Ag–C bonding contacts from unprotected terminal acetylenes for molecular junctions. Nano Lett..

[18] Makk P (2012). Correlation analysis of atomic and single-molecule junction conductance. ACS Nano.

[19] Huang C (2017). Single-molecule detection of dihydroazulene photo-thermal reaction using break junction technique. Nat. Commun..

[20] Chen H (2021). Single-molecule charge transport through positively charged electrostatic anchors. J. Am. Chem. Soc..

[21] Su TA (2013). Silicon ring strain creates high-conductance pathways in single-molecule circuits. J. Am. Chem. Soc..

[22] Kim NT, Li H, Venkataraman L, Leighton JL (2016). High-conductance pathways in ring-strained disilanes by way of direct σ-Si–Si to Au coordination. J. Am. Chem. Soc..

[23] Pan X, Lawson B, Rustad AM, Kamenetska M (2020). pH-activated single molecule conductance and binding mechanism of imidazole on gold. Nano Lett..

[24] Osorio HM (2017). Influence of surface coverage on the formation of 4,4′-bipyridinium (viologen) single molecular junctions. J. Mater. Chem. C..

[25] Chen W (2011). Highly conducting π-conjugated molecular junctions covalently bonded to gold electrodes. J. Am. Chem. Soc..

[26] Zheng J (2018). Electrical and SERS detection of disulfide-mediated dimerization in single-molecule benzene-1,4-dithiol junctions. Chem. Sci..

[27] Vladyka A (2019). In-situ formation of one-dimensional coordination polymersin molecular junctions. Nat. Commun..

[28] Li X (2020). Structure-independent conductance of thiophene-based single-stacking junctions. Angew. Chem. Int. Ed..

[29] Tang Y (2020). Electric field-induced assembly in single-stacking terphenyl junctions. J. Am. Chem. Soc..

[30] Fu T (2019). Enhanced coupling through π-stacking in imidazole-based molecular junctions. Chem. Sci..

[31] Adak O (2015). Flicker noise as a probe of electronic interaction at metal–single molecule interfaces. Nano Lett..

[32] Chen H (2020). Giant conductance enhancement of intramolecular circuits through interchannel gating. Matter.

[33] Tahara K (2006). Two-dimensional porous molecular networks of dehydrobenzo[12]annulene derivatives via alkyl chain interdigitation. J. Am. Chem. Soc..

[34] Park YS (2007). Contact chemistry and single-molecule conductance: a comparison of phosphines, methyl sulfides, and amines. J. Am. Chem. Soc..

[35] Zhan C (2019). Single-molecule measurement of adsorption free energy at the solid–liquid interface. Angew. Chem. Int. Ed..

[36] Hong W (2012). Single molecular conductance of tolanes: experimental and theoretical study on the junction evolution dependent on the anchoring group. J. Am. Chem. Soc..

[37] Afsari S, Li Z, Borguet E (2014). Orientation-controlled single-molecule junctions. Angew. Chem. Int. Ed..

[38] Xiang L (2016). Non-exponential length dependence of conductance in iodide-terminated oligothiophene single-molecule tunneling junctions. J. Am. Chem. Soc..

[39] Meng B, Liu J, Wang L (2020). Oligo(ethylene glycol) as side chains of conjugated polymers for optoelectronic applications. Polym. Chem..

[40] Paulsen BD, Tybrandt K, Stavrinidou E, Rivnay J (2020). Organic mixed ionic–electronic conductors. Nat. Mater..

[41] Meng B (2015). Replacing alkyl with oligo(ethylene glycol) as side chains of conjugated polymers for close π–π stacking. Macromolecules.

[42] Giovannitti A (2016). Controlling the mode of operation of organic transistors through side-chain engineering. Proc. Natl Acad. Sci. USA.

[43] Yang S-F (2017). Diketopyrrolopyrrole-based conjugated polymer entailing triethylene glycols as side chains with high thin-film charge mobility without post-treatments. Adv. Sci..

[44] Driggers EM, Hale SP, Lee J, Terrett NK (2008). The exploration of macrocycles for drug discovery — an underexploited structural class. Nat. Rev. Drug Discov..

[45] Yudin AK (2015). Macrocycles: lessons from the distant past, recent developments, and future directions. Chem. Sci..

[46] Harder P, Grunze M, Dahint R, Whitesides GM, Laibinis PE (1998). Molecular conformation in oligo(ethylene glycol)-terminated self-assembled monolayers on gold and silver surfaces determines their ability to resist protein adsorption. J. Phys. Chem. B.

[47] Chen H, Zhang W, Li M, He G, Guo X (2020). Interface engineering in organic field-effect transistors: principles, applications, and perspectives. Chem. Rev..

[48] Zhou Y (2012). A universal method to produce low-work function electrodes for organic electronics. Science.

[49] Kim DH (2005). Enhancement of field-effect mobility due to surface-mediated molecular ordering in regioregular polythiophene thin film transistors. Adv. Funct. Mater..

