# Supplementary material for: Using automated synthesis to understand the role of side chains on molecular charge transport
Source: Nat Commun. 2022 Apr 19;13:2102. doi: 10.1038/s41467-022-29796-2 (PMC9019014; doi:10.1038/s41467-022-29796-2)
Supplement: Supplementary file 1 — Supplementary Information [file 41467_2022_29796_MOESM1_ESM.pdf]

Supplementary Information for:

Using Automated Synthesis to Understand the Role of Side Chains on Molecular Charge Transport

Songsong Li[1,2,], Edward R. Jira[2,3,*], Nicholas H. Angello[2,4,*], Jialing Li[2,3], Hao Yu[3], Jeffrey S. Moore[1,2,4], Ying Diao[2,3,4], Martin D. Burke[2,4,5], and Charles M. Schroeder[1,2,3,4]*

[1]Department of Materials Science and Engineering, University of Illinois at Urbana-Champaign, Urbana, Illinois, 61801, United States

[2]Beckman Institute for Advanced Science and Technology, University of Illinois at Urbana-Champaign, Urbana, Illinois, 61801, United States

[3]Department of Chemical and Biomolecular Engineering, University of Illinois at Urbana-Champaign, Urbana, Illinois, 61801, United States

[4]Department of Chemistry, University of Illinois at Urbana-Champaign, Urbana, Illinois, 61801, United States

[5]Carle Illinois College of Medicine, University of Illinois at Urbana-Champaign, Urbana, Illinois, 61801, United States

[*]These authors contributed equally to this work.

Correspondence and requests for materials should be addressed to C.M.S. (email: cms@illinois.edu).

Table of Contents:

S.1 General Methods ……………………………………………………………S2

S.2 Synthesis Details……………………………………………………………S5

S.3 Additional Experimental Data…………………………………………………S18

S.4 NMR and UV-vis Dilution Experiment…………………………………………S26

S.5 Analytical kinetic model………………………………………………………S28

S.6 NMR Spectra…………………………………………………………………S30

S.1 General Methods

Materials. Commercial reagents were purchased and used without further purification. 4-Methylthiophenylboronic acid, 1,4-Di-n-butylbenzene, and N-Boc-4-bromoaniline were purchased from Combi-Blocks. XPhos Pd G2, PCy_3 Pd G2, NaOH, TFA, benzyltrimethylammonium tribromide, $ZnCl_2$, bromine, iodine, NaSMe, 1,4-Dipropylbenzene, 1,4-Diisopropylbenzene, 6,12-Dihydroindeno[1,2-b]fluorene, 3-Methyl-4-(methylthio)phenylboronic acid, 1-Bromoheptane, 1-Bromopentane, 1,4-Dibromo-2,5-dimethoxybenzene, 2-Methoxyethyl 4-methylbenzenesulfonate, 2-(2-methoxyethoxy)ethyl 4-methylbenzenesulfonate, 1,4-Dibromo-2,5-bis(decyloxy)benzene, 2,5-dibromo-p-xylene, 2-Methoxyethoxymethyl chloride, 2-Methyl-4-methylthiophenylboronic acid, and K_2CO_3 were purchased from Sigma-Aldrich. K_3PO_4 was purchased from Sigma-Aldrich and, for iterative reactions, finely ground using a hot mortar and pestle (mortar and pestle removed from a 120 °C oven prior to grinding) and stored in a sealed vial in a dry box over blue-indicating Drierite prior to use. 2,5-Dibromohydroquinone was purchased from TCI America. Anhydrous tetrahydrofuran was purchased from Fisher Scientific and further dried by distillation from sodium/benzophenone and stored under Argon over 4Å molecular sieves in a Straus flask prior to use. Anhydrous dioxane, ethyl acetate, *n*-hexane, diethyl ether, methanol, and dichloromethane were purchased from Fisher Scientific.

General experimental procedures. All reactions were carried out under an atmosphere of nitrogen or argon in flame-dried or oven-dried glassware with magnetic stirring unless otherwise indicated. Organic solutions were concentrated via rotary evaporation under reduced pressure with a bath temperature of 40 °C unless otherwise noted. Reactions were monitored by analytical thin layer chromatography (TLC) performed using the indicated solvent on normal phase Merck silica gel 60 F254 plates (0.25mm). Compounds were visualized by exposure to a UV lamp (λ = 254 nm). Normal phase flash column chromatography was performed using Merck silica gel grade 9385 60Å (230-400mesh) and a Biotage Selekt medium pressure liquid chromatography (MPLC) instrument. Preparative high-performance liquid chromatography was performed on an Agilent 1200 series instrument with a Waters SunFire Prep C18 OBD 5 μM 30 x 150 mm column. Automated small molecule synthesis procedures performed on a Burke type small molecule synthesizer.[1]

Characterization

NMR. ^{1}H-NMR and ^{13}C-NMR spectra were recorded on Varian Unity 500, Varian Unity Inova 500NB, Varian Unity 400, or Carver B500 instruments. ^{11}B-NMR were recorded on a Carver B500 spectrometer. Chemical shifts (δ) are reported in parts per million (ppm) downfield from tetramethylsilane and referenced to residual protium in the NMR solvent ($CHCl_3$, $\delta = 7.26$; $(CD_3)_2CO$, $\delta = 2.05$, center line; DMSO $\delta = 2.50$, center line). Data are reported as follows: chemical shift, multiplicity (s = singlet, d = doublet, t = triplet, q = quartet, quint = quintet, sept = septet, m = multiplet, b = broad, app = apparent), coupling constant (J) in Hertz (Hz), and integration. Chemical shifts (δ) for ^{13}C NMR are reported in ppm downfield from tetramethylsilane and referenced to carbon resonances in the NMR solvent (CDCl3, $\delta = 77.0$, center line; $(CD_3)_2CO$, $\delta = 29.8$, center line; DMSO-d6, $\delta = 39.5$, center line). Carbons bearing boron substituents were not observed (quadrupolar relaxation).

Mass Spectrometry. High resolution EI and ESI mass spectra were recorded on a Micromass 70-VSE spectrometer and Micromass Q-TOF Ultima spectrometer. For low resolution mass spectra, data are reported in the form of m/z (intensity relative to the base peak = 100).

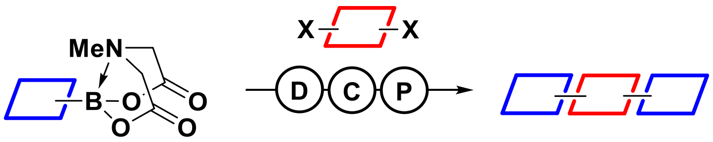

General Automated Procedure I - Experimental Details: To a 10g, polypropylene Luknova column, outfitted with a polyethylene frit and charged with starting MIDA boronate (2.4 mmol) and NaOH (7.2 mmol, 288 mg) is added 10 mL THF followed by 3 mL water. This solution is then agitated by bubbling argon through the solution for 20 minutes at room temperature. After agitation, 3 mL aqueous potassium phosphate buffer (pH=6, 0.5 M) and 5 mL Et_2O are added. The layers are briefly mixed (again, via argon sparging) before being allowed to separate. The aqueous layer is disposed of using a syringe pump pulling from the bottom of the column. Then, 3 mL 50% saturated aqueous NaCl is added, the layers are mixed, and allowed to separate. Again, the aqueous layer is disposed of using a syringe pump. The solution is then passed to a second polypropylene Luknova column with the frit removed. This organic solution is then degassed and concentrated to 10 mL (evaporating most of the Et_2O) via argon sparging.

In the coupling module, a 40 mL I-Chem vial with Teflon septa equipped with a rare earth, Teflon coated stir bar (10 mm diameter) is charged with alkyl dihalide (0.1 mmol), XPhos 2nd generation palladacycle (0.01 mmol, 8 mg, 10 mol%), and K_3PO_4 (1.8 mmol, 382 mg) To this cartridge is added 3 mL of a thoroughly argon sparged 5:1 Dioxane:water mixture. The THF solution of freshly deprotected boronic acid is split evenly and added via syringe pump to four different reaction vials. At the end of the addition, the reaction is heated to 100 °C and stirred for 16 hours. Products were then purified by automated MPLC with ethyl acetate/hexanes eluent.

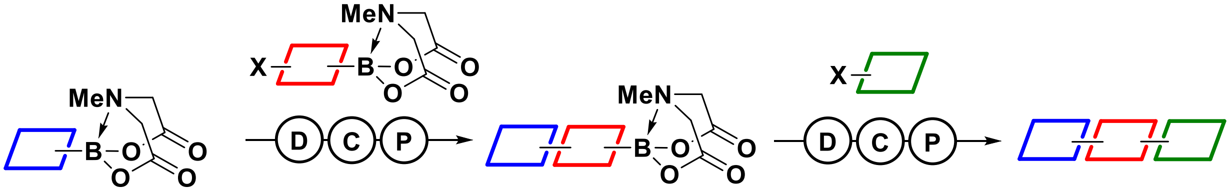

General automated procedure II - Deprotection: To a 10g, polypropylene Luknova column, outfitted with a polyethylene frit and charged with starting MIDA boronate (1.9 mmol, 442 mg) and NaOH (5.7 mmol, 228 mg) is added 10 mL THF followed by 3 mL water. This solution is then agitated by bubbling argon through the solution for 20 minutes at room temperature. After agitation, 3 mL aqueous potassium phosphate buffer (pH=6, 0.5 M) and 5 mL Et_2O are added. The layers are briefly mixed (again, via argon sparging) before being allowed to separate. The aqueous layer is disposed of using a syringe pump pulling from the bottom of the column. Then, 3 mL 50% saturated aqueous NaCl is added, the layers are mixed, and allowed to separate. Again, the aqueous layer is disposed of using a syringe pump. The solution is then transferred to a Luknova column charged with celite (800 mg) and $MgSO_4$ (2.5 g). The solution is dried by repeated cycles of withdrawal and injection into this column (20 repetitions) The solution is then transferred to a Luknova column charged with celite (300 mg, tapped into pellet) and activated molecular sieves (3.6g, powdered, 4 Å) and further dried by repeated withdrawals and injections to this column. (20 repetitions). The solution is then passed into an empty Luknova column with the frit removed before washing the $MgSO_4$ and molecular sieve drying agents sequentially with 6 mL THF and adding the wash to the cartridge

containing boronic acid mixture. This organic solution is then concentrated to 10 mL (evaporating most of the Et_2O) before washing the drying agents with a further 6 mL THF. The organic solution (now only THF) is concentrated to 10 mL. This deoxygenated, dry solution is used directly in the subsequent coupling reaction.

In the coupling module, a 40 mL I-Chem vial with Teflon septa equipped with a rare earth, Teflon coated stir bar (10 mm diameter) is charged with bifunctional MIDA boronate (0.63 mmol, 303 mg), XPhos 2nd generation palladacycle (0.0032 mmol, 2.5 mg, 5 mol%), and K_3PO_4 (5.7 mmol, 1.2 g) The contents of this vial are placed under argon and dissolved in anhydrous THF (3 mL) before being magnetically stirred. The THF solution of freshly deprotected boronic acid is then added via automated syringe pump to this vial. At the end of the addition, the reaction is heated to 60 °C and stirred for 16 hours.

In the purification module, 3 mL of the crude reaction mixture is added to 20 mL of hexanes in a magnetically stirred Precipitation Cartridge (containing 250 mg aminopropyl functionalized silica gel and 150 mg Celite) and connected to a Silica Gel Plug, precipitating the MIDA boronate. The solvent is removed from the cartridge, loading any crude reaction product onto the Silica Gel Plug (“catch”). This process is performed a total of 5 times, using 3 mL THF to wash the Reaction Cartridge twice. Then, 12 mL of 1.5% MeOH in Et_2O is added and the solvent is removed three times (36 mL total). Then, 12 mL of Et_2O is added and the solvent is removed 3 times (36 mL total). Finally, 12 mL THF is added and slowly reverse eluted through the Silica Gel Plug (“release”), giving a purified solution of MIDA boronate.

S.2 Synthesis Details

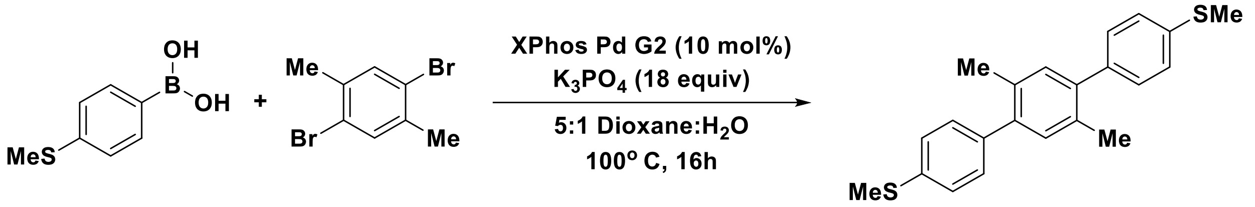

6 equiv 1 equiv R1

Automated Procedure I was followed using 2,5-dibromo-para-xylene (26.4 mg, 0.1 mmol) and (4-thiomethoxyphenyl)boronic acid MIDA ester (167.4 mg, 0.6 mmol). The pure compound **R1** was obtained following purification by flash column chromatography on silica gel (elution: 15% EtOAc in hexanes) as a white solid. (8.76 mg, 0.025 mmol, 25% yield). **^{1}H NMR** (500MHz, ($CDCl_3$) δ 7.32 (d, J = 8.4 Hz, 4H), 7.30 (d, J = 8.4 Hz, 4H), 7.13 (s, 2H), 2.54 (s, 6H), 2.28 (s, 6H).; **^{13}C NMR** (126 MHz, ($CDCl_3$): δ 140.2, 138.4, 136.9, 132.7, 131.8, 129.7, 126.3, 19.9, 15.9.; **HRMS (EI)** calcd for $C_{22}H_{22}S_2$ $[M]^+$ 350.1163, found 350.1169.

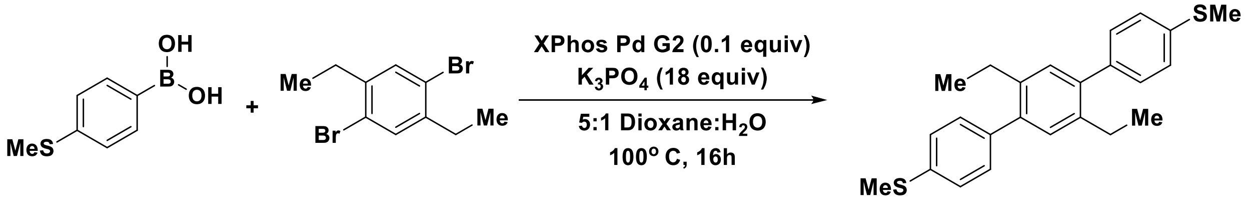

6 equiv 1 equiv R2

Automated Procedure I was followed using 1,4-dibromo-2,5-diethylbenzene (29.2 mg, 0.1 mmol) and (4-thiomethoxyphenyl)boronic acid MIDA ester (167.4 mg, 0.6 mmol). The pure compound **R2** was obtained following purification by flash column chromatography on silica gel (elution: 15% EtOAc in hexanes) as a white solid. (12.42 mg, 0.033 mmol, 33% yield). **^{1}H NMR** (500MHz, ($CDCl_3$): δ 7.31 (dd, J = 13.5, 8.5 Hz, 8H), 7.13 (s, 2H), 2.61 (q, J = 7.5 Hz, 4H), 2.54 (s, 6H), 1.11 (t, J = 7.5 Hz, 6H).**^{13}C NMR** (126 MHz, ($CDCl_3$): δ 140.1, 138.8, 138.7, 136.8, 130.2, 129.7, 126.3, 25.7, 15.9, 15.2.; **HRMS (EI)** calcd for $C_{24}H_{26}S_2$ $[M]^+$ 378.1476, found 378.1481.

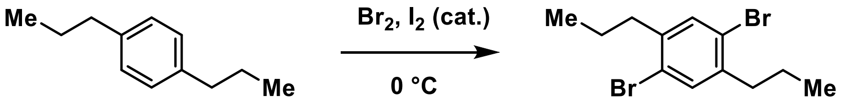

SI-1

SI-1 was prepared in analogy to a literature procedure.[2] To an oven dried 40 mL I-Chem vial with septa cap and equipped with a rare earth Teflon coated stir bar (10 mm diameter) was added 1,4-Dipropylbenzene (956 mg, 5.89 mmol) and a catalytic quantity of I_2 (~1 mol%). At 0 °C, Br_2 (0.607 mL, 11.78 mmol) was added dropwise affording effervescence. The reaction was stirred neat at 0 °C under subdued light. After 16 h, the reaction was quenched with 1M sodium sulfite (15 mL) and extracted with Et_2O (3 x 10 mL). The combined organic layers were dried over Na_2SO_4 and concentrated *in vacuo*. The crude reaction mixture was recrystallized from hot EtOH, yielding **SI-1** as a white crystalline solid (1.48 g, 4.63 mmol, 79% yield). **^{1}H NMR** (500MHz, $CDCl_3$): δ 7.36 (s, 2H), 2.63 (t, J = 7.6 Hz, 4H), 1.68 – 1.57 (m, 4H), 0.98 (t, J = 7.3 Hz, 6H). **^{13}C NMR** (126 MHz, $CDCl_3$): δ 141.1, 133.8, 123.1, 37.5, 23.0, 13.8. **HRMS (EI)** calcd for $C_{12}H_{16}Br_2$ $[M]^+$ 317.9619, found 317.9627.

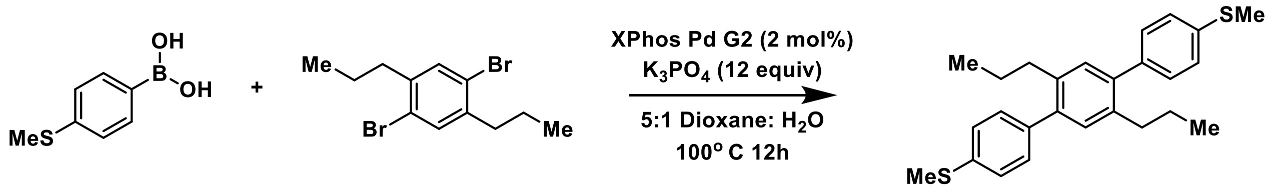

4 equiv **1 equiv** **R3**

A 40 mL I-Chem vial with Teflon septa equipped with a rare earth Teflon coated stir bar (10 mm diameter) containing SI-1 (640 mg, 2 mmol), was charged with 4-(Methylthio)phenylboronic acid (1.34 g, 8 mmol), XPhos Pd G2 (31.4 mg, 0.04 mmol, 2 mol%), and K_3PO_4 (5.09 g, 24 mmol) and was subsequently dissolved in 5:1 dioxane:H_2O (10 mL, 0.2 M). The reaction was heated to 100 °C and magnetically stirred at 300 rpm for 12 h. The reaction was transferred to a separatory with H_2O (2 x 20 mL) and Et_2O (2 x 20 mL) and extracted with Et_2O (3 x 20 mL). The combined organic layers were dried over Na_2SO_4 and concentrated *in vacuo*. The crude reaction mixture was recrystallized from hot EtOH, and the precipitate further washed with cold EtOH (~20 mL), yielding R3 as a white crystalline solid (514.5 mg, 1.27 mmol, 63 % yield). **^{1}H NMR** (500 MHz, $CDCl_3$): δ 7.32 (d, J = 8.3 Hz, 4H), 7.28 (d, J = 8.4 Hz, 4H), 7.10 (s, 2H), 2.58 – 2.50 (m, 10H), 1.53 – 1.45 (m, 4H), 0.83 (t, J = 7.3 Hz, 6H). **^{13}C NMR** (126 MHz, $CDCl_3$): δ 140.2, 138.8, 137.3, 136.7, 130.9, 129.7, 126.2, 34.7, 24.5, 15.9, 14.1. **HRMS (EI)** calcd for $C_{26}H_{30}S_2$ $[M]^+$ 406.1789, found 406.1797.

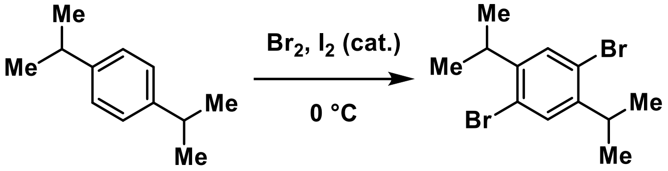

SI-2

SI-2 was prepared following a literature procedure.[2] To an oven dried 40 mL I-Chem vial with septa cap and equipped with a rare earth Teflon coated stir bar (10 mm diameter) was added 1,4-Diisopropylbenzene (1 g, 6.16 mmol) and a catalytic quantity of I_2 (~1 mol%). At 0 °C, Br_2 (0.635 mL, 12.3 mmol) was added dropwise affording effervescence. The reaction was stirred neat at 0 °C under subdued light. After 16 h, the reaction was quenched with 1M sodium sulfite (15 mL) and extracted with Et_2O (3 x 10 mL). The combined organic layers were dried over Na_2SO_4 and concentrated *in vacuo*. The crude reaction mixture was recrystallized from hot EtOH, yielding **SI-2** as a white crystalline solid (592 mg, 1.85 mmol, 30% yield). The spectra are consistent with that reported in the literature.[2] **^{1}H NMR** (500MHz, $CDCl_3$): δ 7.40 (s, 2H), 3.36 – 3.22 (m, 2H), 1.23 (d, J = 7.0 Hz, 12H). **^{13}C NMR** (126 MHz, $CDCl_3$): δ 146.5, 130.7, 123.4, 32.6, 22.7.

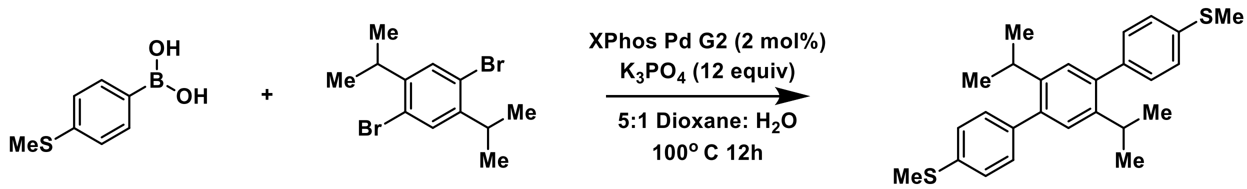

4 equiv **1 equiv** **R3-iPr**

A 40 mL I-Chem vial with Teflon septa equipped with a rare earth Teflon coated stir bar (10 mm diameter) containing SI-2 (592 mg, 1.85 mmol), was charged with 4-(methylthio)phenylboronic acid (1.24 g, 7.4 mmol), XPhos Pd G2 (31.4 mg, 0.04 mmol, ~2 mol%), and K_3PO_4 (4.71 g, 22.2 mmol) and was subsequently dissolved in 5:1 dioxane:H_2O (10 mL, ~0.2 M). The reaction was heated to 100 °C and magnetically stirred at 300 rpm for 12 h. The reaction was transferred to a separatory funnel with H_2O (2 x 20 mL) and Et_2O (2 x 20 mL) and extracted with Et_2O (3 x 20 mL). The combined organic layers were dried over Na_2SO_4 and concentrated *in vacuo*. The crude reaction mixture was recrystallized from hot EtOH, and the precipitate further washed with cold EtOH (~20 mL), yielding **R3-iPr** as a white crystalline solid (492 mg, 1.21 mmol, 65 % yield). **^{1}H NMR** (500 MHz, $CDCl_3$): δ 7.33 (d, J = 8.2 Hz, 4H), 7.28 (d, J = 8.3 Hz, 4H), 7.14 (s, 2H), 3.06 (h, J = 6.9 Hz, 2H), 2.55 (s, 6H), 1.15 (d, J = 6.8 Hz, 12H). **^{13}C NMR** (126 MHz, $CDCl_3$): δ 143.3, 139.8, 139.0, 136.8, 129.9, 127.1, 126.2, 29.1, 24.3, 15.9. **HRMS (EI)** calcd for $C_{26}H_{30}S_2$ $[M]^+$ 406.1789, found 406.1790.

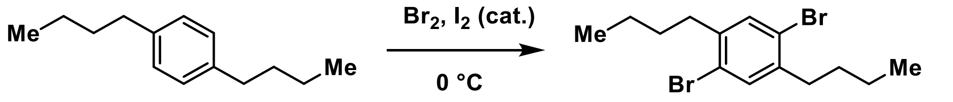

SI-3

SI-3 was prepared following a literature procedure.[2] To an oven dried 40 mL I-Chem vial with septa cap and equipped with a rare earth Teflon coated stir bar (10 mm diameter) was added 1,4-di-n-butylbenzene (907 mg, 4.77 mmol) and a catalytic quantity of I_2 (~1 mol%). At 0 °C, Br_2 (0.492 mL, 9.54 mmol) was added dropwise affording effervescence. The reaction was and stirred neat under subdued light. After 16 h, the reaction was quenched with 1M sodium sulfite (15 mL) and extracted with Et_2O (3 x 10 mL). The combined organic layers were dried over Na_2SO_4 and concentrated *in vacuo*. The crude reaction mixture was recrystallized from hot EtOH, yielding **SI-3** as a white crystalline solid (1.26 g, 3.62 mmol, 76 % yield). The spectra are consistent with that reported in the literature.[2] **^{1}H NMR** (500MHz, $CDCl_3$): δ 7.36 (s, 2H), 2.66 (t, J = 7.6 Hz, 4H), 1.52 – 1.60 (m, 4H), 1.34 – 1.44 (m, 4H), 0.98 (t, J = 7.3 Hz, 6H); **^{13}C NMR** (126 MHz, $CDCl_3$): δ 141.3, 133.8, 123.0, 35.2, 32.6, 22.4, 13.9.

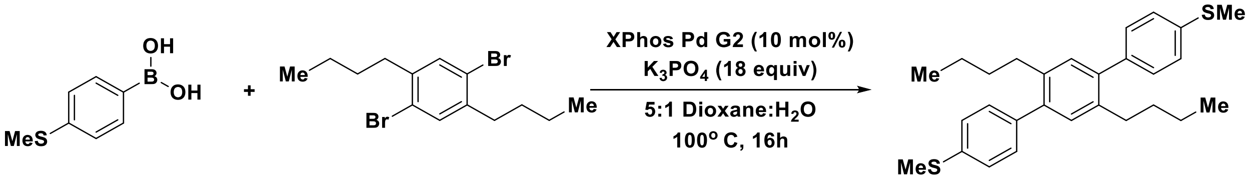

6 equiv 1 equiv R4

Automated Procedure I was followed using dihalide **SI-3** (34.8 mg, 0.1 mmol) and (4-thiomethoxyphenyl)boronic acid MIDA ester (167.4 mg, 0.6 mmol). The pure compound **R4** was obtained following purification by flash column chromatography on silica gel (elution: 15% EtOAc in hexanes) as a white solid. (9.1 mg, 0.021 mmol, 21% yield). **^{1}H NMR** (500MHz, ($CDCl_3$): δ 7.32 (d, J = 8.2 Hz, 4H), 7.28 (d, J = 8.3 Hz, 4H), 7.10 (s, 2H), 2.65 – 2.33 (m, 10H), 1.51 – 1.39 (m, 4H), 1.34 – 1.16 (m, 4H), 0.80 (t, J = 7.3 Hz, 6H). **^{13}C NMR** (126 MHz, $CDCl_3$): δ 140.1, 138.8, 137.5, 136.7, 130.9, 129.8, 126.2, 33.6, 32.3, 22.6, 15.9, 13.9.; **HRMS (EI)** calcd for $C_{28}H_{34}S_2$ $[M]^+$ 434.2102, found 434.2099.

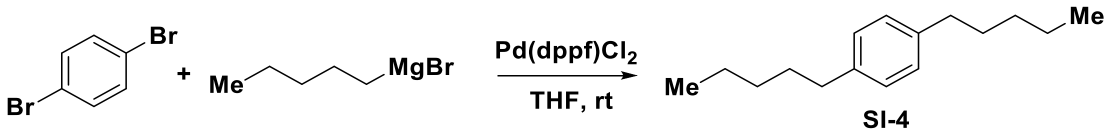

SI-4 was prepared following a modified literature procedure.[3] To an oven dried 40 mL I-Chem vial with septa cap and equipped with a rare earth Teflon coated stir bar (10 mm diameter) was added 1,4-dibromobenzene (708 mg, 3 mmol), Pd(dppf)Cl_2 (132 mg, 0.18 mmol), and THF (18 mL) followed by dropwise addition of the pentylmagnesium bromide solution (4.9 mL, 2M in Et_2O). The reaction was stirred for 48h at rt. The reaction changed color from orange to red upon addition of the Grignard reagent, and after 24h, appeared as a darkened, dull red color with additional precipitate formation. After 48h, the reaction was quenched by pouring into 25 mL 1M HCl and extracted with Et_2O (2 x 20 mL). The combined organic layers were dried over Na_2SO_4 and concentrated *in vacuo* (bath temp: 35 °C). The crude reaction mixture, an oil with red solid, was loaded onto a 10 g MPLC column with hexanes (1 mL) and purified by flash chromatography (elution: hexanes), yielding **SI-4** as a clear oil (408 mg, 1.87 mmol, 62% yield). The spectra are consistent with that reported in the literature.[2] ^{1}**H NMR** (500MHz, ($CDCl_3$): δ 7.10 (s, 4H), 2.57 (d, J = 7.6 Hz, 4H), 1.65 – 1.57 (m, 4H), 1.39 – 1.29 (m, 8H), 0.90 (t, J = 6.7 Hz, 6H)

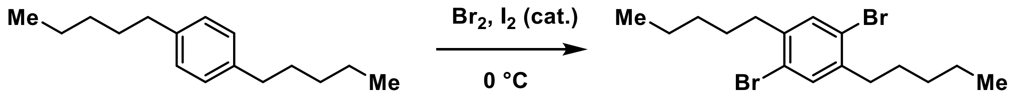

SI-5 was prepared following a literature procedure.[2] To an oven dried 40 mL I-Chem vial with septa cap and equipped with a rare earth Teflon coated stir bar (10 mm diameter) was added **SI-4** (408 mg, 1.87 mmol) and a catalytic quantity of I_2 (~1 mol%). At 0 °C, Br_2 (0.145 mL, 5.61 mmol) was added dropwise affording effervescence. The reaction was warmed to room temperature and stirred neat under subdued light. After 16 h, the reaction was quenched with 1M sodium sulfite (15 mL) and extracted with Et_2O (3 x 10 mL). The combined organic layers were dried over Na_2SO_4 and concentrated *in vacuo*. The crude reaction mixture, a yellow oil, was loaded onto a 10 g MPLC column with hexanes (1 mL) and purified by flash chromatography (elution: hexanes), yielding **SI-5** as a clear oil which solidifies upon cooling to a white solid (699 mg, 2.01 mmol, 98% yield). The spectra are consistent with that reported in the literature.[3] ^{1}**H NMR** (500MHz, $CDCl_3$): δ 7.35 (s, 2H), 2.64 (d, J = 7.7 Hz, 4H), 1.64 – 1.55 (m, 4H), 1.41 – 1.32 (m, 8H), 0.91 (d, J = 6.9 Hz, 6H). ^{13}**C NMR** (126 MHz, $CDCl_3$): δ 141.3, 133.7, 123.0, 35.5, 31.5, 29.5, 22.5, 14.0.

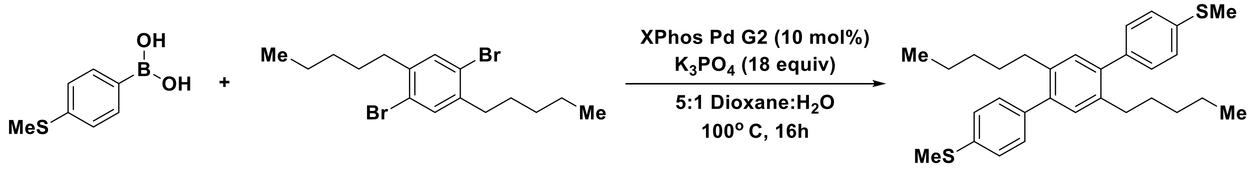

6 equiv 1 equiv R5

Automated Procedure I was followed using 37.6 mg (0.1 mmol) dihalide **SI-5** and 167.4 mg (0.6 mmol) (4-thiomethoxyphenyl)boronic acid MIDA ester. The pure compound **R5** was obtained following purification by flash column chromatography on silica gel (elution: 15% EtOAc in hexanes) as a white solid. (7.18 mg, 0.016 mmol, 16% yield). ^{1}**H NMR** (500MHz, ($CDCl_3$): δ 7.32 (d, J = 8.1 Hz, 4H), 7.30 (d, J = 8.1 Hz, 4H), 7.12 (s, 2H), 2.58 (t, J = 7.8 Hz, 4H), 2.55 (s, 6H), 1.50 (p, J = 7.2 Hz, 4H), 1.27 – 1.17 (m, 6H), 0.82 (t, J = 6.6 Hz, 6H); ^{13}**C NMR** (126 MHz, $CDCl_3$): δ 140.1, 138.7, 137.5, 136.7, 130.9, 129.7, 126.2, 32.5, 31.7, 31.1, 22.3, 15.9, 13.9.; **HRMS (EI)** calcd for $C_{30}H_{38}S_2$ $[M]^+$ 462.2415, found 462.2431.

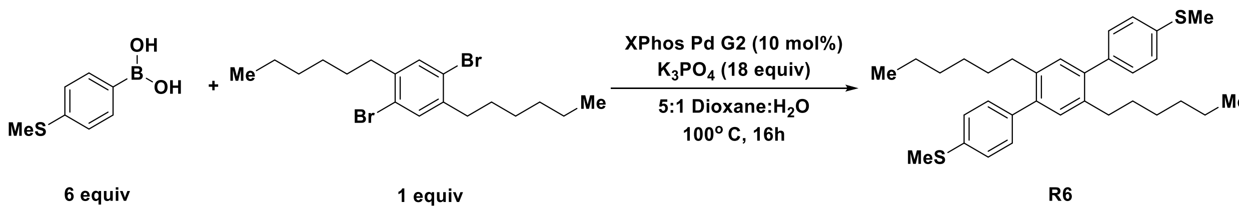

Automated Procedure I was followed using 40.4 mg (0.1 mmol) dihalide 1,4-dibromo-2,5-di-n-hexylbenzene and 167.4 mg (0.6 mmol) MIDA boronate S-1. The pure compound **R6** was obtained following purification by flash column chromatography on silica gel (elution: 15% EtOAc in hexanes) as a white solid. (35.06 mg, 0.072 mmol, 72% yield). **^{1}H NMR** (500MHz, ($CDCl_3$): δ 7.24 (d, J = 8.1 Hz, 4H), 7.21 (d, J = 8.1 Hz, 4H), 7.02 (s, 2H), 2.53 – 2.40 (m, 10H), 1.45 – 1.34 (m, 4H), 1.18 – 1.04 (m, 12H), 0.75 (t, J = 6.9 Hz, 6H). **^{13}C NMR** (126 MHz, $(CD_3)_2CO$): δ 140.1, 138.8, 137.6, 136.7, 130.9, 129.7, 126.2, 32.6, 31.5, 31.4, 29.2, 22.5, 15.9, 14.0.; **HRMS (EI)** calcd for $C_{32}H_{42}S_2$ $[M]^+$ 490.2728, found 490.2724.

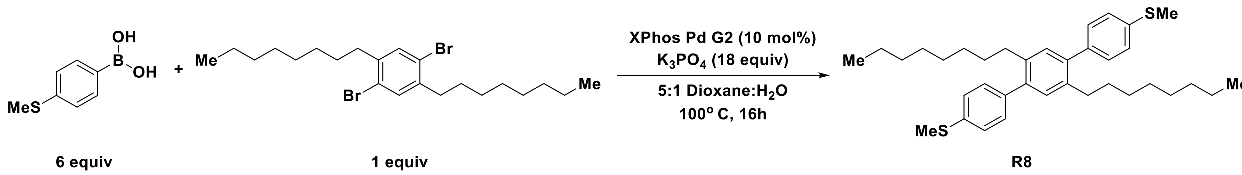

Automated Procedure I was followed using 55.4 mg (0.1 mmol) dihalide 88c and 167.4 mg (0.6 mmol) MIDA boronate S-1. The pure compound **R8** was obtained following purification by flash column chromatography on silica gel (elution: 15% EtOAc in hexanes) as a white solid. (25.75 mg, 0.047 mmol, 47% yield). **^{1}H NMR** (500MHz, ($CDCl_3$): δ 7.31 (dd, J = 16.6, 8.4 Hz, 8H), 7.11 (s, 2H), 2.57 (t, J = 7.6 Hz, 4H), 2.55 (s, 6H), 1.49 (p, J = 5.7 Hz, 4H), 1.4 – 1.1 (m, 20H), 0.9 (t, J = 7.1 Hz, 6H).; **^{13}C NMR** (126 MHz, ($CDCl_3$): δ 140.1, 138.7, 137.5, 136.7, 130.9, 129.7, 126.2, 32.6, 31.8, 31.4, 29.5, 29.3, 29.1, 22.6, 15.9, 14.1.; **HRMS (EI)** calcd for $C_{36}H_{50}S_2$ $[M]^+$ 546.3354, found 546.3362.

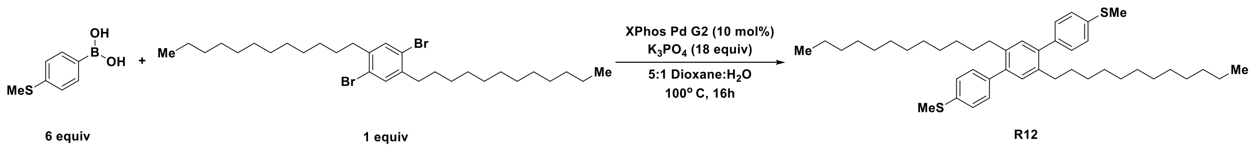

Automated Procedure I was followed using 66.7 mg (0.1 mmol) 1,4-didodecyl-2,5-diiodobenzene and 167.4 mg (0.6 mmol) MIDA boronate S-1. The pure compound **R12** was obtained following purification by flash column chromatography on silica gel (elution: 5% EtOAc in hexanes) as a white solid. (26.6 mg, 0.040 mmol, 40% yield). **^{1}H NMR** (500MHz, ($CDCl_3$): δ 7.24 (d, J = 8.3 Hz, 4H), 7.21 (d, J = 8.2 Hz, 4H), 7.02 (s, 2H), 2.51 – 2.42 (m, 10H), 1.43 – 1.35 (m, 4H), 1.24 – 1.06 (m, 36H), 0.80 (t, J = 6.8 Hz, 6H); **^{13}C NMR** (126 MHz, ($CDCl_3$): δ 140.1, 138.8, 137.6, 136.7, 130.9, 129.7, 126.2, 32.6, 31.9, 31.4, 29.7, 29.6, 29.6, 29.5, 29.5, 29.3, 29.3, 22.7, 15.9, 14.1.; **HRMS (EI)** calcd for $C_{44}H_{66}S_2$ $[M]^+$ 658.4606, found 658.4609.

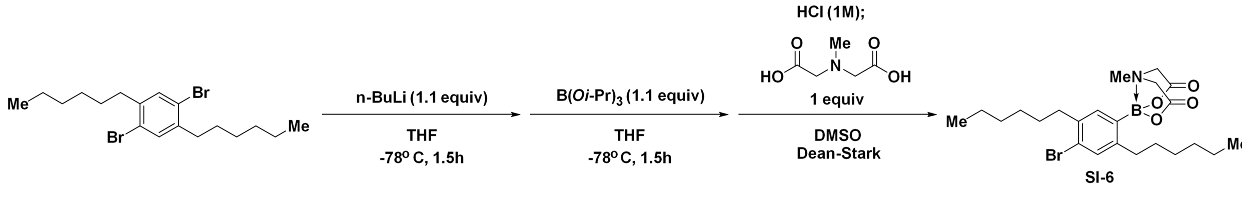

To a flame-dried, 300 ml round bottom flask, equipped with a rare earth Teflon coated stir bar (10 mm diameter) was added 1,4-dibromo-2,5-di-n-hexylbenzene (877 mg, 2.17 mmol). The flask was backfilled with nitrogen and the halide was dissolved in THF (100 mL). The solution was cooled to -78 °C and stirred. To the stirred solution was added *s*-butyl Lithium dropwise (1.55 mL, 1.4 M in cyclohexane, 2.17 mmol). The resulting mixture was stirred for 90 minutes before adding triisopropyl borate (0.5 mL, 2.17 mmol). The resulting solution was stirred for an additional 60 minutes. The mixture was then allowed to warm to 0 °C and subsequently quenched with aqueous HCl (1M, 5 mL). The solution was then diluted with EtOAc (30 mL) and washed with water (3 x 30 mL). The aqueous layers were combined and extracted further with EtOAc (2 x 30 mL). All organic layers were combined and dried over $NaSO_4$. To the EtOAc solution was added DMSO (10 mL). The EtOAc was then removed via concentrated in vacuo to afford a DMSO solution. This solution was immediately transferred to a 300 mL round bottom flask equipped with a rare earth Teflon coated stir bar (10 mm diameter). To the flask was added toluene (100 mL) and N-methyliminodiacetic acid (319 mg, 2.17 mmol). The flask was then equipped with a dean stark apparatus and heated to reflux for 12 hours. The resulting solution was concentrated *in vacuo*. The pure compound **SI-6** (303 mg, 0.63 mmol, 29% yield) was obtained following purification by flash column chromatography on silica gel (elution: 0 → 20% EtOAc in hexanes) as a white solid. **^{1}H NMR** (500MHz, $CDCl_3$): δ 7.38 (s, 1H), 7.20 (s, 1H), 3.97 (d, J = 16.6 Hz, 2H), 3.76 (d, J = 16.6 Hz, 2H), 2.66 (t, J = 7.9 Hz, 2H), 2.59 (s, 3H), 2.51 (t, J = 7.7 Hz, 2H), 1.64 – 1.51 (m, 4H), 1.41 – 1.27 (m, 12H), 0.88 (t, J = 6.8 Hz, 6H); **^{13}C NMR** (126 MHz, $CDCl_3$): δ 167.2, 147.2, 139.3, 135.5, 134.4, 126.5, 62.6, 47.6, 35.9, 35.8, 32.8, 31.7, 31.6, 30.3, 29.5, 29.2, 22.7, 22.6, 14.1; **^{11}B NMR** (160 MHz, $CDCl_3$): δ 12.4; **HRMS (ES)** calcd for $C_{23}H_{36}BBrNO_4$ $[M + H]^+$ 480.1921, found 480.1912.

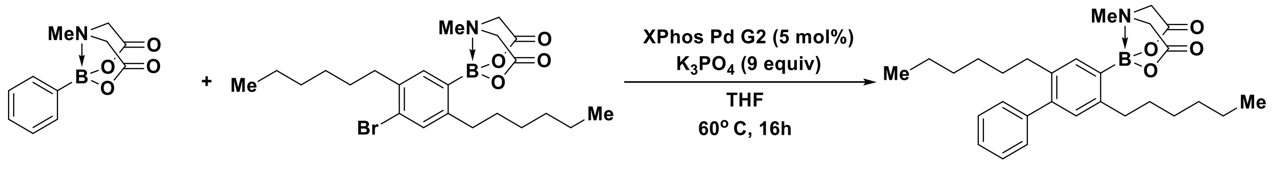

3 equiv 1 equiv SI-7

Automated Procedure II was followed using bifunctional MIDA boronate **SI-6** (303 mg, 0.63 mmol) and phenyl MIDA boronate (442.7 mg, 1.9 mmol). The pure compound **SI-7** was obtained following purification by flash column chromatography on silica gel (elution: 0 → 20% EtOAc in hexanes) as a white solid. (122 mg, 0.226 mmol, 36% yield). **^{1}H NMR** (500MHz, $CDCl_3$): δ 7.44 – 7.27 (m, 6H), 7.06 (s, 1H), 3.94 (d, J = 16.5 Hz, 2H), 3.80 (d, J = 16.5 Hz, 2H), 2.66 (s, 3H), 2.58 (t, J = 8.0 Hz, 2H), 2.52 (t, J = 8.0 Hz, 2H), 1.67 – 1.59 (m, 2H), 1.42 – 1.34 (m, 4H), 1.33 – 1.27 (m, 4H), 1.21 – 1.09 (m, 6H), 0.87 (t, J = 6.8 Hz, 3H), 0.80 (t, J = 6.8 Hz, 3H); **^{13}C NMR** (126 MHz, $CDCl_3$): δ 167.2, 145.0, 143.2, 141.8, 137.4, 134.8, 132.1, 129.1, 128.0, 126.8, 62.5, 47.5, 36.2, 33.1, 32.7, 31.8, 31.7, 31.5, 29.6, 29.2, 22.6, 22.5, 14.1, 14.0; **^{11}B NMR** (160 MHz, $CDCl_3$): δ 13.9; **HRMS (ES)** calcd for $C_{29}H_{41}BNO_4$ $[M + H]^+$ 478.3129, found 478.3117.

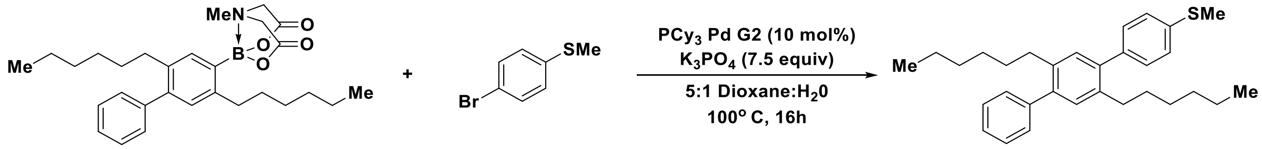

1.3 equiv 1 equiv R6-H

Automated Procedure I was followed using **SI-7** (63 mg, 0.13 mmol) and 4-bromothioanisole (20.3 mg, 0.1 mmol), K_3PO_4 (159 mg, 0.75 mmol), and PCy3 Pd G2 (5.9 mg, 0.01 mmol, 10 mol%). The pure compound **R6-H** was obtained following purification by flash column chromatography on silica gel

(elution: 5% EtOAc in hexanes) as a clear oil (24.5 mg, 0.055 mmol, 55% yield). **^{1}H NMR** (500MHz, $CDCl_3$): δ 7.42 – 7.13 (m, 9H), 7.10 – 6.98 (m, 2H), 2.66 – 2.35 (m, 7H), 1.49 – 1.36 (m, 4H), 1.20 – 1.05 (m, 12H), 0.73 (t, J = 6.7 Hz, 6H); **^{13}C NMR** (126 MHz, $CDCl_3$): δ 141.9, 140.8, 140.1, 138.8, 137.5, 137.5, 136.7, 130.9, 130.8, 129.8, 129.3, 128.0, 126.7, 126.2, 32.6, 31.5, 31.5, 31.4, 31.3, 29.2, 29.2, 22.5, 22.5, 15.9, 14.0; **HRMS (EI)** calcd for $C_{31}H_{40}S$ $[M]^+$ 444.2851, found 444.2867.

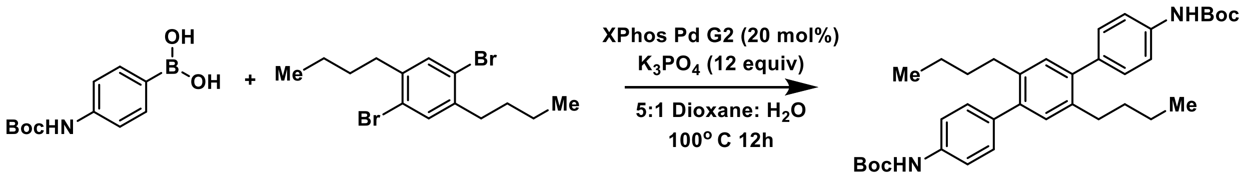

4 equiv **1 equiv** **R4-NHBoc**

In an unoptimized procedure, a 40 mL I-Chem vial with Teflon septa equipped with a rare earth Teflon coated stir bar (10 mm diameter) containing dihalide **SI-3** (1.47 g, 4.2 mmol), was charged with 4-Boc-aminophenylboronic acid (4 g, 16.9 mmol), XPhos Pd G2 (670 mg, 0.85 mmol, 20 mol%), and K_3PO_4 (10.7 g, 50.8 mmol) and was subsequently dissolved in 5:1 dioxane:H_2O (21 mL, 0.2 M) and sparged with Argon for 20 minutes. The reaction was heated to 100 °C and magnetically stirred at 300 rpm for 12 h. After cooling to rt, the reaction was concentrated *in vacuo* and dissolved in DCM (20 mL) and washed with water (2 x 20 mL). The aqueous layer was extracted further with DCM (2 x 10 mL). All organic layers were combined and dried over $NaSO_4$, filtered, and concentrated *in vacuo*. Recrystallization from hot MeOH/EtOH yielded the product at ~80% purity. This material was further purified by flash column chromatography on silica gel (elution: 5 → 35% EtOAc in hexanes), yielding **R4-NHBoc (**377 mg, 0.66 mmol, 16% yield) as a white solid. **^{1}H NMR** (500MHz, ($CDCl_3$): δ 7.41 (d, J = 8.1 Hz, 4H), 7.28 (d, J = 8.3 Hz, 4H), 7.08 (s, 2H), 6.52 (s, 2H), 2.56 (t, J = 7.8 Hz, 4H), 1.54 (s, 18H), 1.49 – 1.42 (m, 4H), 1.26 – 1.20 (m, 4H), 0.80 (t, J = 7.3 Hz, 6H).**^{13}C NMR** (126 MHz, $CDCl_3$): δ 152.8, 140.1, 137.5, 137.0, 136.7, 130.9, 129.9, 118.1, 80.6, 33.6, 32.3, 28.4, 22.6, 13.9. **HRMS (ES)** calcd for $C_{36}H_{48}N_2O_4Na$ $[M + Na]^+$ 595.3512, found 595.3497.

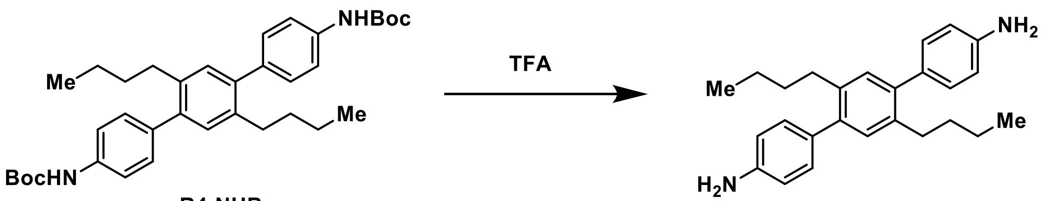

R4-NHBoc **R4-N**

Synthesized following a modified literature procedure.[4] A 40 mL I-Chem vial with Teflon septa equipped with a rare earth Teflon coated stir bar (10 mm diameter) containing protected amine **R4-NHBoc** (87.2 mg, 0.15 mmol) was charged with TFA (1.0 mL, 13.1 mmol) and stirred for 30 minutes. The reaction mixture was diluted with DCM (20 mL) and washed with 1M NaOH (3 x 10 mL). The aqueous layers were combined and extracted further with DCM (2 x 10 mL). All organic layers were combined and dried with Na_2SO_4 and concentrated *in vacuo* to afford **R4-N** as a white solid which did not require further purification (46.4 mg, 0.125 mmol, 83% yield). **^{1}H NMR** (500MHz, ($CDCl_3$): δ 7.16 (d, J = 8.0 Hz, 4H), 7.09 (s, 2H), 6.74 (d, J = 8.0 Hz, 4H), 3.65 (br s, 4H), 2.57 (t, J = 7.8 Hz, 4H), 1.51 – 1.44 (m, 4H), 1.28 – 1.20 (m, 4H), 0.81 (t, J = 7.3 Hz, 6H).**^{13}C NMR** (126 MHz, $CDCl_3$): δ 144.9, 140.2, 137.5, 132.4, 131.0, 130.2, 114.7, 33.7, 32.4, 22.7, 13.9. **HRMS (ES)** calcd for $C_{26}H_{33}N_2$ $[M + H]^+$ 373.2644, found 373.2633.

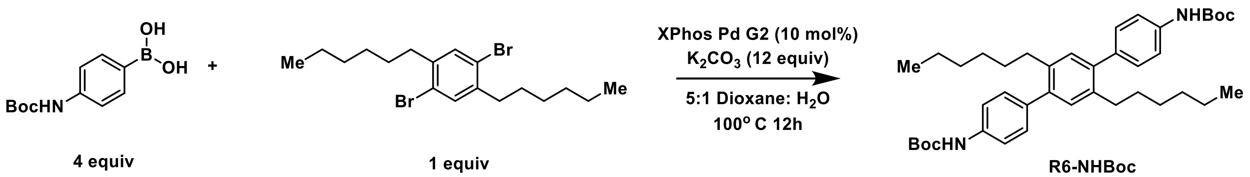

4 equiv 1 equiv R6-NHBoc

In an unoptimized procedure, a 40 mL I-Chem vial with Teflon septa equipped with a rare earth Teflon coated stir bar (10 mm diameter) containing 1,4-dibromo-2,5-dihexylbenzene (404 mg, 1 mmol), was charged with 4-Boc-aminophenylboronic acid (948 mg, 4 mmol), XPhos Pd G2 (78.6 mg, 0.1 mmol, 10 mol%), and K_2CO_3 (1.66 g, 12 mmol) and was subsequently dissolved in 5:1 dioxane:H_2O (10 mL, 0.1 M) and sparged with Argon for 20 minutes. The reaction was heated to 100 °C and magnetically stirred at 300 rpm for 12 h. After cooling to rt, the reaction was concentrated *in vacuo* and dissolved in DCM (20 mL), washed with water (2 x 20 mL), dried with Na_2SO_4, filtered, and concentrated *in vacuo*. The pure compound **R6-NHBoc** (87 mg, 0.14 mmol, 14% yield) was obtained following purification by flash column chromatography on silica gel (elution: 10 → 30% EtOAc in hexanes) as a white solid. **^{1}H NMR** (500MHz, $CDCl_3$): δ 7 7.41 (d, J = 8.1 Hz, 4H), 7.28 (d, J = 8.1 Hz, 4H), 7.08 (s, 2H), 6.51 (s, 2H), 2.55 (d, J = 8.0 Hz, 4H), 1.54 (s, 18H), 1.49 – 1.43 (m, 4H), 1.23 – 1.14 (m, 12H), 0.82 (t, J = 7.0 Hz, 6H).; **^{13}C NMR** (126 MHz, $CDCl_3$): δ 152.8, 140.1, 137.5, 137.0, 136.8, 130.9, 129.9, 118.1, 80.6, 32.6, 31.5, 31.4, 29.2, 28.4, 22.5, 14.0.; **HRMS (ES)** calcd for $C_{40}H_{56}N_2O_4Na$ $[M + Na]^+$ 651.4138, found 651.4117.

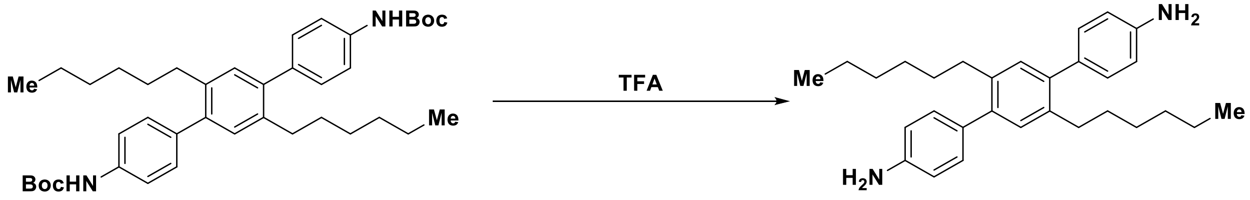

R6-N

Synthesized following a modified literature procedure.[4] A 40 mL I-Chem vial with Teflon septa equipped with a rare earth Teflon coated stir bar (10 mm diameter) containing protected amine **R6-NHBoc** (60 mg, 0.095 mmol) was charged with TFA (1.0 mL, 13.1 mmol) and stirred for 30 minutes. The reaction mixture was diluted with DCM (20 mL) and washed with 1M NaOH (3 x 10 mL). The aqueous layers were combined and extracted further with DCM (2 x 10 mL). All organic layers were combined and dried with Na_2SO_4 and concentrated *in vacuo* to afford **R6-N** as a white solid which did not require further purification (14.4 mg, 0.034 mmol, 35% yield). **^{1}H NMR** (500MHz, $(CD_3)_2SO$): δ 6.97 (d, J = 8.2 Hz, 4H), 6.93 (s, 2H), 6.61 (d, J = 8.2 Hz, 4H), 5.14 (s, 4H), 2.52 (t, J = 7.7 Hz, 4H), 1.43 – 1.37 (m, 4H), 1.22 – 1.13 (m, 12H), 0.80 (t, J = 6.9 Hz, 6H).; **^{13}C NMR** (126 MHz, $(CD_3)_2SO$): δ 147.2, 139.9, 136.7, 130.6, 129.4, 128.9, 113.6, 32.1, 30.9, 30.8, 28.6, 21.9, 13.9; **HRMS (ES)** calcd for $C_{30}H_{41}N_2$ $[M + H]^+$ 429.3270, found 429.3258.

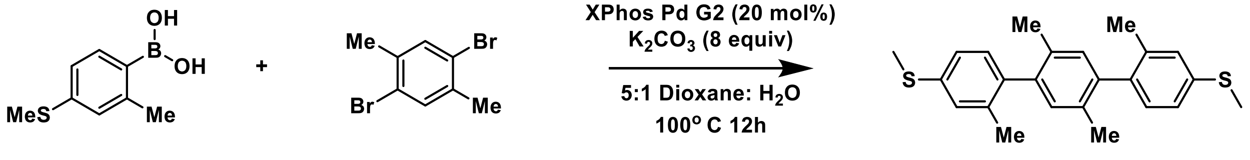

4 equiv 1 equiv R1-twisted

A 40 mL I-Chem vial with Teflon septa equipped with a rare earth Teflon coated stir bar (10 mm diameter) containing 2,5-dibromo-p-xylene (132 mg, 0.5 mmol), was charged with 2-methyl-4-methylthiophenylboronic acid (364 mg, 2 mmol), XPhos Pd G2 (78.6 mg, 0.1 mmol, 20 mol%), and K_2CO_3

(553 mg, 4 mmol) and was subsequently dissolved in 5:1 dioxane:H_2O (5 mL, 0.1 M) and sparged with Argon for 20 minutes. The reaction was heated to 100 °C and magnetically stirred at 300 rpm for 12 h. The pure compound **R1-twisted** (7.6 mg, 0.02 mmol, 4% yield) was obtained following purification by flash column chromatography on silica gel (elution: 0 → 30% EtOAc in hexanes) as a white solid. **^{1}H NMR** (500MHz, $CDCl_3$): δ 7.19 (s, 2H), 7.15 (d, J = 7.9 Hz, 2H), 7.10 (dd, J = 7.9, 3.7 Hz, 2H), 6.98 (s, 1H), 6.96 (s, 1H), 2.54 (s, 6H), 2.11 (s, 3H), 2.10 (s, 3H), 2.04 (s, 3H), 2.03 (s, 3H).**^{13}C NMR** (126 MHz, $CDCl_3$): δ 139.8, 139.8, 138.6, 138.5, 136.7, 136.6, 136.6, 136.6, 132.9, 132.8, 130.8, 130.7, 129.9, 129.8, 127.9, 123.7, 123.7, 20.0, 19.8, 19.3, 15.9, 15.9.; **HRMS (EI)** calcd for $C_{24}H_{26}S_2$ $[M]^+$ 378.1476, found 378.1483.

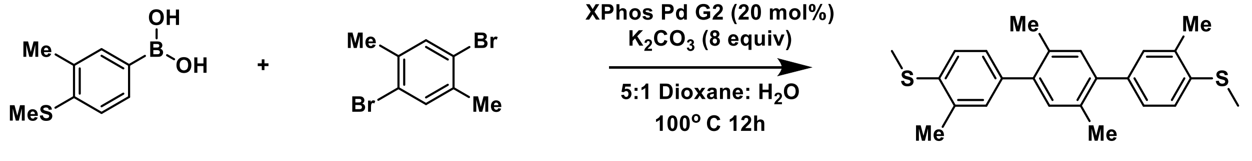

4 equiv **1 equiv** **R1-*m*-Me**

A 40 mL I-Chem vial with Teflon septa equipped with a rare earth Teflon coated stir bar (10 mm diameter) containing 2,5-dibromo-p-xylene (132 mg, 0.5 mmol), was charged with 3-methyl-4-(methylthio)phenylboronic acid (364 mg, 2 mmol), XPhos Pd G2 (78.6 mg, 0.1 mmol, 20 mol%), and K_2CO_3 (553 mg, 4 mmol) and was subsequently dissolved in 5:1 dioxane:H_2O (5 mL, 0.1 M) and sparged with Argon for 20 minutes. The reaction was heated to 100 °C and magnetically stirred at 300 rpm for 12 h. The pure compound **R1-*m*-Me** (47 mg, 0.125 mmol, 25% yield) was obtained following purification by flash column chromatography on silica gel (elution: 0 → 30% EtOAc in hexanes) as a white solid. **^{1}H NMR** (500MHz, $CDCl_3$): δ 7.23 (s, 4H), 7.18 (s, 2H), 7.14 (s, 2H), 2.53 (s, 6H), 2.41 (s, 6H), 2.30 (s, 6H); **^{13}C NMR** (126 MHz, $CDCl_3$): δ 140.2, 138.1, 136.1, 135.4, 132.6, 131.8, 130.6, 127.3, 124.2, 20.0, 19.9, 15.3. **HRMS (EI)** calcd for $C_{24}H_{26}S_2$ $[M]^+$ 378.1476, found 378.1478.

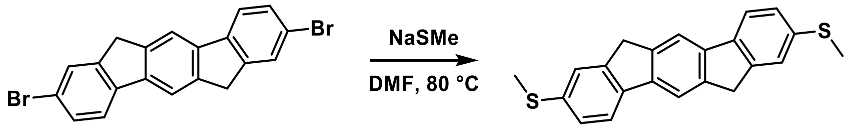

R1-planar

A 40 mL I-Chem vial with Teflon septa equipped with a rare earth Teflon coated stir bar (10 mm diameter) containing 2,8-dibromo-6,12-dihydroindeno[1,2-b]fluorene[5] (240 mg, 0.58 mmol), under argon was charged with sodium methanethiolate (244 mg, 3.48 mmol) and dissolved in anhydrous DMF (6 mL, 0.1 M). The reaction was heated to 80 °C and magnetically stirred at 300 rpm for 12 h. After cooling to rt, the resulting precipitates were filtered, washed with water (20 mL), and dried *in vacuo*. The tan solid was then purified by vacuum sublimation (water chilled cold finger, ~5 mTorr vacuum, oil bath at 180 °C for 24h then 200 °C for 24h), yielding **R1-planar** as a white solid (6 mg, 0.02 mmol, 4% yield). **HRMS (EI)** calcd for $C_{22}H_{18}S_2$ $[M]^+$ 346.0850, found 346.0859. **MS** (EI, m/z): 346.1 (M^+, 85), 252.1 (100). Insolubility of the compound precluded NMR characterization.

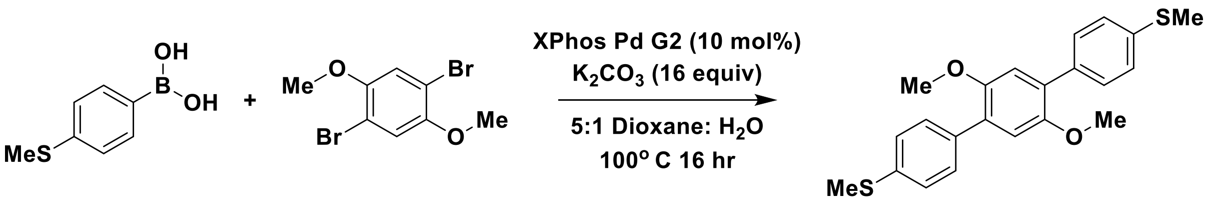

4 equiv **1 equiv** **RO1**

A 40 mL I-Chem vial with Teflon septa equipped with a rare earth Teflon coated stir bar (10 mm diameter) containing 1,4-dibromo-2,5-dimethoxybenzene (592 mg, 2 mmol), was charged with 4-(Methylthio)phenylboronic acid (1.34 g, 8 mmol), XPhos Pd G2 (160 mg, 0.2 mmol, 10 mol%), and K_2CO_3 (4.4 g, 32 mmol) and was subsequently dissolved in 5:1 dioxane:H_2O (20 mL, 0.1 M). The reaction was heated to 100 °C and magnetically stirred at 300 rpm for 12 h. The pure compound **RO1** (6 mg, 0.016 mmol, 0.8% yield) was obtained following purification by flash column chromatography on silica gel (elution: 5 → 15% EtOAc in hexanes) as a white solid. **^{1}H NMR** (500 MHz, Chloroform-*d*) δ 7.52 (d, *J* = 8.2 Hz, 4H), 7.33 (d, *J* = 8.4 Hz, 4H), 6.95 (s, 2H), 3.79 (s, 6H), 2.53 (s, 6H). **^{13}C NMR** (126 MHz, $CDCl_3$): *δ* 150.67, 137.35, 135.03, 129.80, 129.73, 126.33, 114.50, 56.43, 15.89. **HRMS (EI)** calcd for $C_{22}H_{22}O_2S_2$ $[M]^+$ 382.1061, found 382.1076.

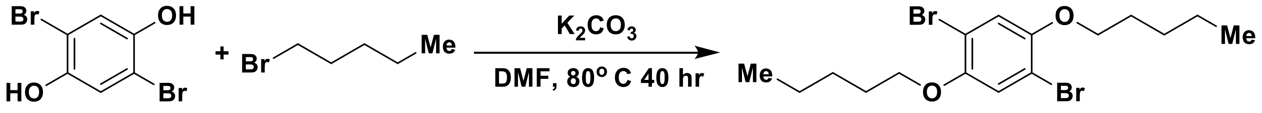

1 equiv 2 equiv RO5-Br

RO5-Br was prepared in analogy to a literature procedure.[6] To an oven dried 40 mL I-Chem vial with septa cap and equipped with a rare earth Teflon coated stir bar (10 mm diameter) was added 2,5-dibromohydroquinone (267 mg, 1 mmol) and K_2CO_3 (828 mg, 6 mmol), and DMF (5 mL) followed by addition of 1-bromopentane (302 mg, 2 mmol). The reaction was stirred for 40h at 80° C. After 48h, the reaction was quenched by addition of 1M HCl (7 ml) and stored at 4 ºC for 4 hours. After 4 hours, a large amount of white precipitate was visible in solution. The solution was filtered and the filtrate was washed with H_2O and dried in vacuo to afford pure **RO5-Br** as a white solid (347 mg, 0.85 mmol, 85% yield). **^{1}H NMR** (500 MHz, $CDCl_3$): *δ* 7.09 (s, 2H), 3.95 (t, *J* = 6.5 Hz, 4H), 1.81 (p, *J* = 6.8 Hz, 4H), 1.51 – 1.34 (m, 8H), 0.94 (t, *J* = 7.1 Hz, 6H). **^{13}C NMR** (126 MHz, $CDCl_3$): *δ* 150.1, 118.5, 111.1, 70.3, 28.8, 28.1, 22.4, 14.0. **HRMS (EI)** calcd for $C_{12}H_{16}O_2Br_2$ $[M]^+$ 406.0143, found 406.0139.

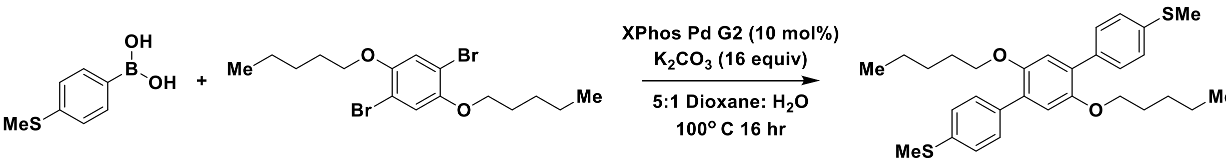

4 equiv 1 equiv RO5

A 40 mL I-Chem vial with Teflon septa equipped with a rare earth Teflon coated stir bar (10 mm diameter) containing **RO5-Br** (176 mg, 0.5 mmol), was charged with 4-(Methylthio)phenylboronic acid (336 mg, 2 mmol), XPhos Pd G2 (39.3 mg, 0.05 mmol, 10 mol%), and K_2CO_3 (1.11 g, 8 mmol) and was subsequently dissolved in 5:1 dioxane:H2O (10 mL, 0.05 M). The reaction was heated to 100 °C and magnetically stirred at 300 rpm for 12 h. The pure compound **RO5** (86 mg, 0.17 mmol, 35% yield) was obtained following purification by preparatory HPLC on C18 silica gel (elution: 0 → 100% MeCN in water) as a white solid.**^{1}H NMR** (500 MHz, $CDCl_3$): *δ* 7.54 (d, *J* = 8.4 Hz, 4H), 7.31 (d, *J* = 8.4 Hz, 4H), 6.95 (s, 2H), 3.90 (t, *J* = 6.5 Hz, 4H), 1.69 (p, *J* = 6.7 Hz, 4H), 1.39 – 1.27 (m, 8H), 0.88 (t, *J* = 6.9 Hz, 6H). **^{13}C NMR** (126 MHz, $CDCl_3$): *δ* 150.25, 136.97, 135.16, 130.05, 129.84, 126.10, 115.99, 69.56, 28.98, 28.20, 22.29, 15.84, 13.99. **HRMS (EI)** calcd for $C_{30}H_{38}O_2S_2$ $[M]^+$ 494.2313, found 494.2311.

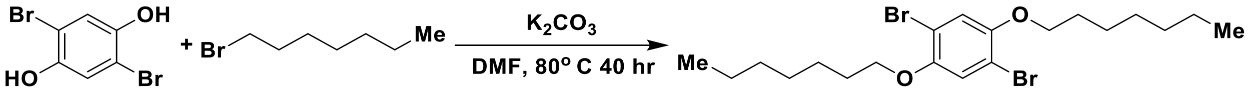

1 equiv 2 equiv RO7-Br

RO7-Br was prepared in analogy to a literature procedure.[6] To an oven dried 40 mL I-Chem vial with septa cap and equipped with a rare earth Teflon coated stir bar (10 mm diameter) was added 2,5-dibromohydroquinone (267 mg, 1 mmol) and K_2CO_3 (828 mg, 6 mmol), and DMF (5 mL) followed by addition of 1-bromopentane (302 mg, 2 mmol). The reaction was stirred for 40h at 80 °C. After 48h, the reaction was quenched by addition of 1M HCl (7 ml) and stored at 4 °C for 4 hours. After 4 hours, a large amount of white precipitate was visible in solution. The solution was filtered and the filtrate was washed with H_2O and dried in vacuo to afford pure **RO7-Br** as a white solid (377 mg, 0.82 mmol, 82% yield). **^{1}H NMR** (500 MHz, $CDCl_3$): δ 7.08 (s, 2H), 3.94 (t, J = 6.5 Hz, 4H), 1.85 – 1.75 (m, 4H), 1.52 – 1.43 (m, 4H), 1.41 – 1.26 (m, 12H), 0.89 (t, J = 6.7 Hz, 6H). **^{13}C NMR** (126 MHz, $CDCl_3$): δ 150.08, 118.47, 111.13, 70.31, 31.75, 29.12, 28.97, 25.89, 22.59, 14.08. **HRMS (EI)** calcd for $C_{20}H_{32}O_2Br_2$ [M]$^+$ 462.0769, found 462.0782.

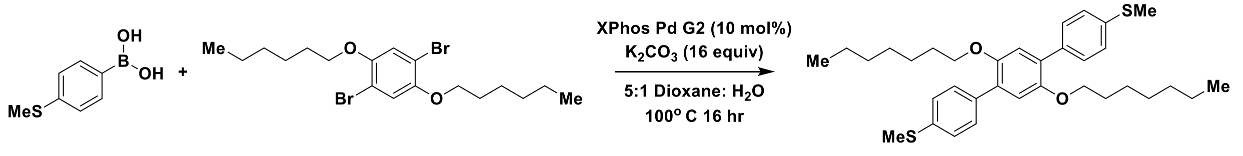

A 40 mL I-Chem vial with Teflon septa equipped with a rare earth Teflon coated stir bar (10 mm diameter) containing **RO7-Br** (232 mg, 0.5 mmol), was charged with 4-(Methylthio)phenylboronic acid (336 mg, 2 mmol), XPhos Pd G2 (78.6 mg, 0.1 mmol, 20 mol%), and K_2CO_3 (1.11 g, 8 mmol) and was subsequently dissolved in 5:1 dioxane:H2O (10 mL, 0.05 M). The reaction was heated to 100 °C and magnetically stirred at 300 rpm for 12 h. The pure compound **RO7** (110 mg, 0.20 mmol, 20% yield) was obtained following purification by flash column chromatography on silica gel (elution: 5 → 25% EtOAc in hexanes) as a white solid.**^{1}H NMR** (500 MHz, $CDCl_3$): δ 7.55 (d, J = 8.1 Hz, 4H), 7.31 (d, J = 8.1 Hz, 4H), 6.96 (s, 2H), 3.91 (t, J = 6.5 Hz, 4H), 2.54 (s, 6H), 1.69 (p, J = 6.7 Hz, 4H), 1.41 – 1.20 (m, 16H), 0.89 (t, J = 6.7 Hz, 6H). **^{13}C NMR** (126 MHz, $CDCl_3$): δ 150.26, 136.98, 135.19, 130.07, 129.87, 126.13, 116.00, 69.59, 31.75, 29.32, 28.91, 26.01, 22.55, 15.88, 14.08. **HRMS (EI)** calcd for $C_{34}H_{46}O_2S_2$ [M]$^+$ 550.2939, found 550.2925.

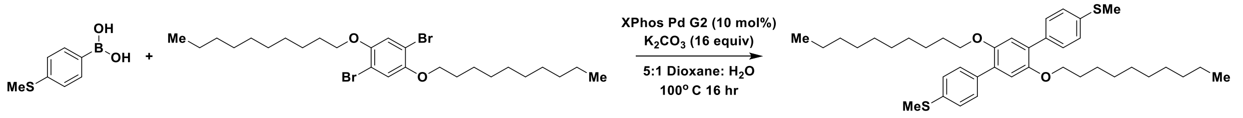

In an unoptimized procedure, a 40 mL I-Chem vial with Teflon septa equipped with a rare earth Teflon coated stir bar (10 mm diameter) containing 1,4-Dibromo-2,5-bis(decyloxy)benzene (54.8 mg, 0.1 mmol), was charged with 4-(Methylthio)phenylboronic acid (100.8 mg, 0.6 mmol), XPhos Pd G2 (7.9 mg, 0.01 mmol, 10 mol%), and K_2CO_3 (249 mg, 1.8 mmol) and was subsequently dissolved in 5:1 dioxane:H2O (10 mL). The reaction was heated to 100 °C and magnetically stirred at 300 rpm for 12 h. The pure compound **RO10** (57 mg, 0.09 mmol, 90% yield) was obtained following purification by flash column chromatography on silica gel (elution: 0 → 10% EtOAc in hexanes) as a white solid. **^{1}H NMR** (500 MHz, $CDCl_3$): δ 7.55 (d, J = 8.1 Hz, 4H), 7.32 (d, J = 8.1 Hz, 4H), 6.97 (s, 2H), 3.91 (t, J = 6.5 Hz, 4H), 2.54 (s, 6H), 1.69 (p, J = 6.7 Hz, 4H), 1.46 – 1.18 (m, 28H), 0.90 (t, J = 6.8 Hz, 6H). **^{13}C NMR** (126 MHz, $CDCl_3$): δ 150.31, 137.01, 135.24, 130.13, 129.87, 126.19, 116.08, 69.65, 31.89, 29.57, 29.52, 29.34, 29.32, 29.27, 26.07, 22.67, 15.91, 14.09. **HRMS (EI)** calcd for $C_{40}H_{58}O_2S_2$ [M]$^+$ 634.3878, found 634.3863.

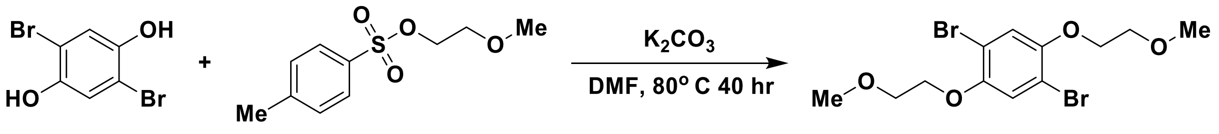

1 equiv 2.3 equiv O2-Br

O2-Br was prepared in analogy to a literature procedure.[6] To an oven dried 40 mL I-Chem vial with septa cap and equipped with a rare earth Teflon coated stir bar (10 mm diameter) was added 2,5-dibromohydroquinone (294.7 mg, 1.1 mmol) and K_2CO_3 (828 mg, 6 mmol), and DMF (5 mL) followed by addition of 2-methoxy-ethyl p-toluenesulfonyloxy ester (590 mg, 2.5 mmol). The reaction was stirred for 40h at 80° C. After 40h, the reaction was quenched by addition of 1M HCl (7 ml) and stored at 4 °C for 4 hours. After 4 hours, a large amount of white precipitate was visible in solution. The solution was filtered and the filtrate was washed with H_2O and dried in vacuo to afford pure **O2-Br** as a white solid (393 mg, 1.02 mmol, 93% yield). **^{1}H NMR** (500 MHz, $CDCl_3$): δ 7.15 (s, 2H), 4.11 (t, J = 4.8 Hz, 4H), 3.77 (t, J = 4.7 Hz, 4H), 3.47 (s, 6H). **^{13}C NMR** (126 MHz, $CDCl_3$): δ 150.37, 119.25, 111.49, 70.84, 70.10, 59.42. **HRMS (ES)** calcd for $C_{12}H_{16}O_4NaBr_2$ $[M + Na]^+$ 404.9313, found 404.9301.

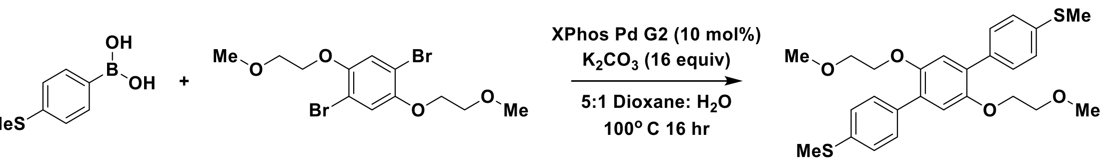

4 equiv 1 equiv O2

In an unoptimized procedure, a 40 mL I-Chem vial with Teflon septa equipped with a rare earth Teflon coated stir bar (10 mm diameter) containing **O2-Br** (192 mg, 0.5 mmol), was charged with 4-(Methylthio)phenylboronic acid (336 mg, 2 mmol), XPhos Pd G2 (40 mg, 0.05 mmol, 10 mol%), and K_2CO_3 (1.93 g, 14 mmol) and was subsequently dissolved in 5:1 dioxane:H2O (10 mL). The reaction was heated to 100 °C and magnetically stirred at 300 rpm for 12 h. The pure compound **O2** (11 mg, 0.02 mmol, 4% yield) was obtained following purification by flash column chromatography on silica gel (elution: 5 ⟶ 25% EtOAc in hexanes) as a white solid. **^{1}H NMR** (500 MHz, $CDCl_3$) δ 7.37 (d, J = 8.3 Hz, 4H), 7.11 (d, J = 8.1 Hz, 4H), 6.80 (s, 2H), 3.90 (t, J = 5.0 Hz, 4H), 3.56 (t, J = 5.0 Hz, 4H), 3.42 (t, J = 5.0, 4.2 Hz, 4H), 3.32 (t, J = 5.1, 4.1 Hz, 4H), 3.17 (s, 6H), 2.35 (s, 6H). **^{13}C NMR** (126 MHz, $CDCl_3$): δ 150.51, 137.21, 134.81, 130.53, 129.84, 126.16, 126.08, 116.96, 71.14, 69.38, 59.13, 15.85. **HRMS (EI)** calcd for $C_{26}H_{30}O_4S_2$ $[M]^+$ 470.1586, found 470.1571.

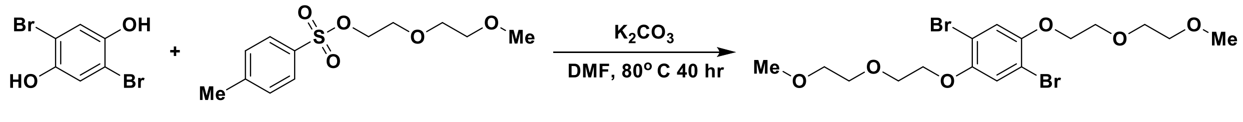

1 equiv 2.2 equiv O3-Br

O3-Br was prepared in analogy to a literature procedure.[6] To an oven dried 40 mL I-Chem vial with septa cap and equipped with a rare earth Teflon coated stir bar (10 mm diameter) was added 2,5-dibromohydroquinone (293 mg, 1.1 mmol) and K_2CO_3 (828 mg, 6 mmol), and DMF (5 mL) followed by addition of toluene-4-sulfonic acid 2-(2-methoxyethoxy)ethyl ester (658 mg, 2.4 mmol). The reaction was stirred for 40h at 80° C. After 40h, the reaction was quenched by addition of 1M HCl (7 ml) and stored at 4 °C for 4 hours. After 4 hours, a large amount of white precipitate was visible in solution. The solution was filtered and the filtrate was washed with H_2O and dried in vacuo to afford pure **O3-Br** as a white solid (386 mg, 0.82 mmol, 82% yield). **^{1}H NMR** (500 MHz, $CDCl_3$): δ 7.15 (s, 2H), 4.13 (t, J = 4.9 Hz, 4H), 3.88 (t, J = 4.8 Hz, 4H), 3.76 (t, J = 4.9, 4.3 Hz, 4H), 3.58 (t, J = 4.9, 4.4 Hz, 4H), 3.40 (s, 6H). **^{13}C NMR** (126

MHz, $CDCl_3$): δ 150.31, 119.15, 111.37, 72.02, 71.00, 70.22, 69.58, 59.10. **HRMS (ES)** calcd for $C_{16}H_{25}O_6Br_2$ $[M + H]^+$ 471.0018, found 471.0017.

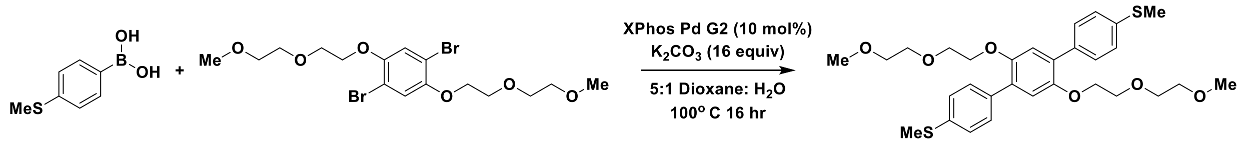

4 equiv 1 equiv O3

In an unoptimized procedure, a 40 mL I-Chem vial with Teflon septa equipped with a rare earth Teflon coated stir bar (10 mm diameter) containing **O3-Br** (236 mg, 0.5 mmol), was charged with 4-(Methylthio)phenylboronic acid (336 mg, 2 mmol), XPhos Pd G2 (40 mg, 0.05 mmol, 10 mol%), and K_2CO_3 (1.93 g, 14 mmol) and was subsequently dissolved in 5:1 dioxane:H_2O (10 mL). The reaction was heated to 100 °C and magnetically stirred at 300 rpm for 12 h. The pure compound **O3** (11 mg, 0.02 mmol, 4% yield) was obtained following purification by flash column chromatography on silica gel (elution: 0 → 5% MeOH in Dichloromethane) as a yellow solid.^{1}**H NMR** (500 MHz, $CDCl_3$): δ 7.37 (d, J = 8.3 Hz, 4H), 7.11 (d, J = 8.1 Hz, 4H), 6.80 (s, 2H), 3.90 (t, J = 5.0 Hz, 4H), 3.56 (t, J = 5.0 Hz, 4H), 3.42 (t, J = 5.0, 4.2 Hz, 4H), 3.32 (t, J = 5.1, 4.1 Hz, 4H), 3.17 (s, 6H), 2.35 (s, 6H). ^{13}**C NMR** (126 MHz, $CDCl_3$): δ 150.34, 137.16, 134.88, 130.35, 129.88, 126.15, 116.53, 71.95, 70.68, 69.77, 69.30, 59.04, 15.87. **HRMS (EI)** calcd for $C_{30}H_{38}O_6S_2$ $[M]^+$ 558.2110, found 558.2100.

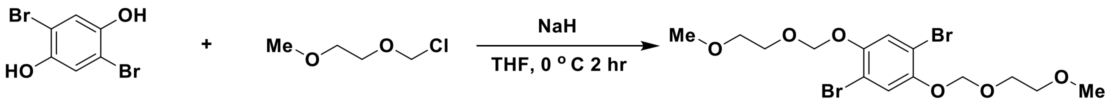

1 equiv 3 equiv MEM-Br

MEM-Br was prepared following a modified literature procedure.[7] A solution of 2,5-dibromohydroquinone (1.34 g, 5 mmol) in THF (10 mL) was added dropwise to a suspension of NaH (rinsed 3 x 5 mL hexanes to remove mineral oil, 300 mg, 12.5 mmol) in THF (7 mL) at 0 °C under a constant flow of nitrogen in an oven dried 40 mL I-Chem vial with septa cap and equipped with a rare earth Teflon coated stir bar (10 mm diameter). After 5 min of stirring, 2-Methoxyethoxymethyl chloride (1.7 mL, 15 mmol) was added slowly, and the reaction mixture was stirred for 2 h at 0 °C. Upon completion, the reaction contents were quenched by the careful addition of water (20 mL) and extracted with EtOAc (3 × 20 mL). The combined organic layers were then washed with 1 M NaOH (2 × 20 mL) and brine (20 mL), dried (Na_2SO_4), and concentrated *in vacuo* to afford **MEM-Br** (2.15 g, 4.83 mmol, 97% yield) as a white powder which did not require further purification. ^{1}**H NMR** (500 MHz, $(CD_3)_2CO$): δ 7.51 (s, 2H), 5.31 (s, 4H), 3.87 – 3.81 (m, 4H), 3.56 – 3.51 (m, 4H), 3.29 (s, 6H). ^{13}**C NMR** (126 MHz, $(CD_3)_2CO$): δ 150.1, 122.2, 112.6, 95.6, 72.2, 68.9, 58.8. **HRMS (EI)** calcd for $C_{14}H_{20}O_6Br_2$ $[M]^+$ 441.9627, found 441.9626.

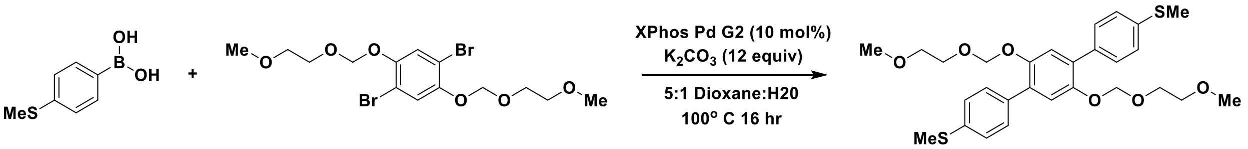

4 equiv 1 equiv MEM

In an unoptimized procedure, a 40 mL I-Chem vial with Teflon septa equipped with a rare earth Teflon coated stir bar (10 mm diameter) containing **MEM-Br** (444 mg, 1 mmol), was charged with 4-(methylthio)phenylboronic acid (672 mg, 4 mmol), XPhos Pd G2 (78 mg, 0.1 mmol, 10 mol%), and K_2CO_3 (1.66 g, 12 mmol) and was subsequently dissolved in 5:1 dioxane:H_2O (20 mL). The reaction was heated to 100 °C and magnetically stirred at 300 rpm for 16 h. The pure compound **MEM** (41 mg, 0.08 mmol, 8% yield) was obtained following purification by flash column chromatography on silica gel (elution: 25 →

50% EtOAc in hexanes) as a white solid. **^{1}H NMR** (500 MHz, $CDCl_3$): δ 7.50 (d, J = 8.2 Hz, 4H), 7.30 (d, J = 8.2 Hz, 4H), 7.22 (s, 2H), 5.14 (s, 4H), 3.73 – 3.68 (m, 4H), 3.50 – 3.46 (m, 4H), 3.34 (s, 6H), 2.53 (s, 6H). **^{13}C NMR** (126 MHz, $CDCl_3$): δ 149.4, 137.4, 134.7, 131.4, 129.9, 126.2, 118.9, 94.9, 71.5, 67.7, 59.0, 15.8. **HRMS (EI)** calcd for $C_{28}H_{34}O_6S_2$ $[M]^+$ 530.1797, found 530.1801.

S.3 Additional experimental data and density functional theory simulations

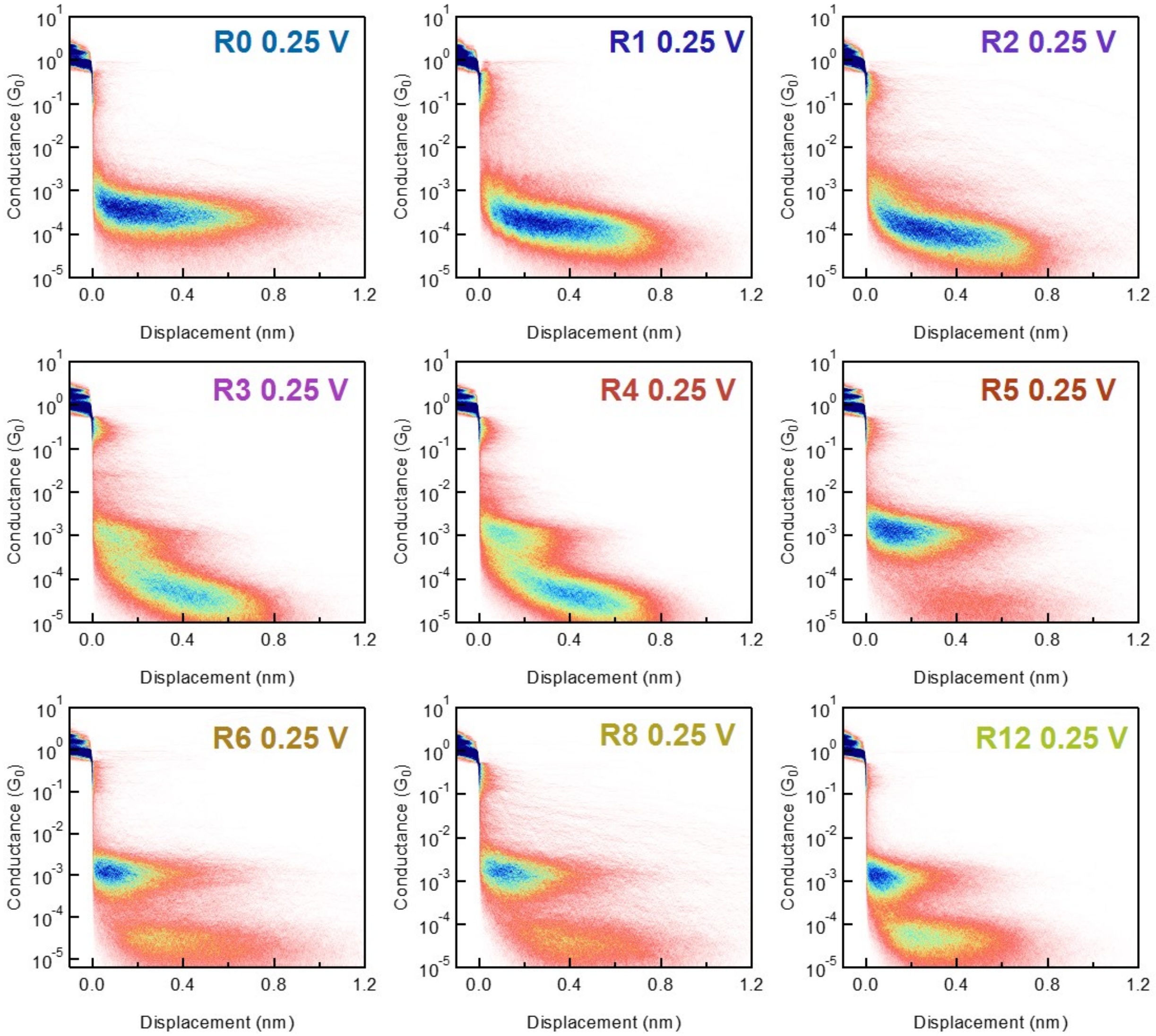

Supplementary Fig. 1 | 2D conductance histograms for **Rn** at an applied bias of 0.25 V.

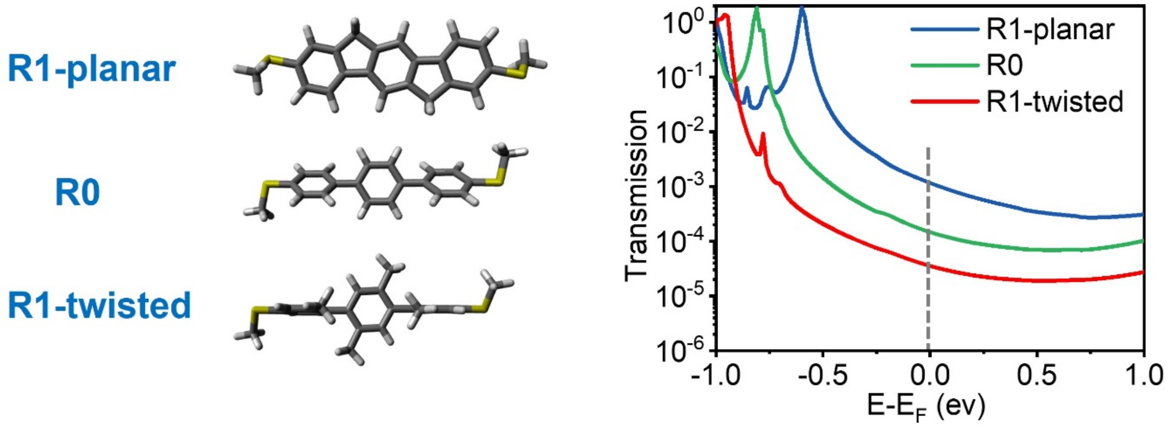

Supplementary Fig. 2 | Transmission spectra from NEGF-DFT simulations for terphenyl derivatives with different conformations.

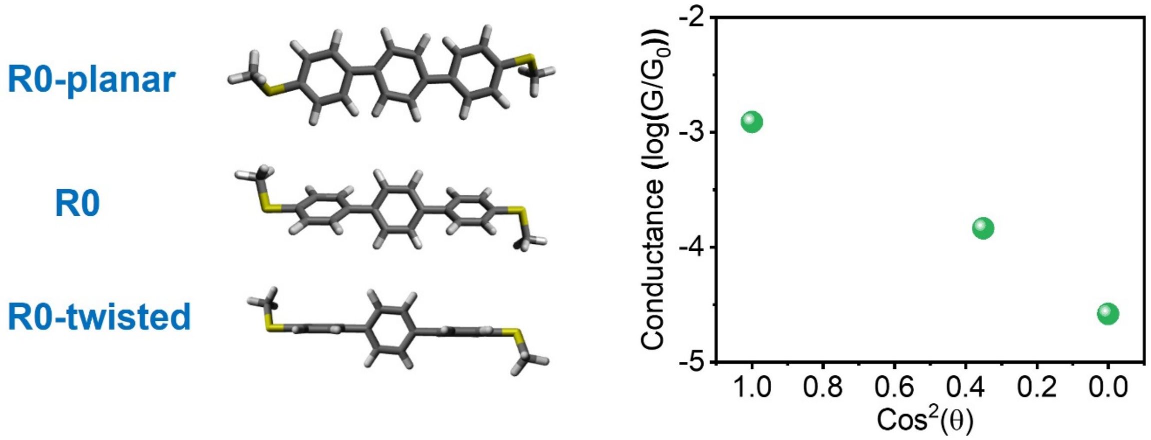

Supplementary Fig. 3 | Peak molecular conductance values for **R0** with different dihedral angles from DFT simulations. These results still agree with the values in **Figure 3b**. Therefore, we can exclude the effect of side chain on the simulated conductance and further prove the role of backbone conformation on molecular charge transport.

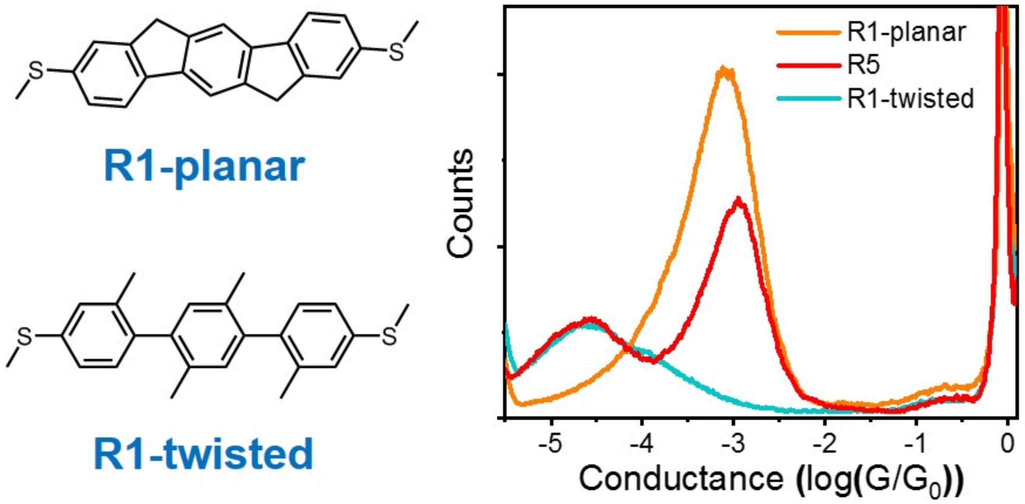

Supplementary Fig. 4 | 1D conductance histograms for **R1-planar** and **R1-twisted** at 0.25 V.

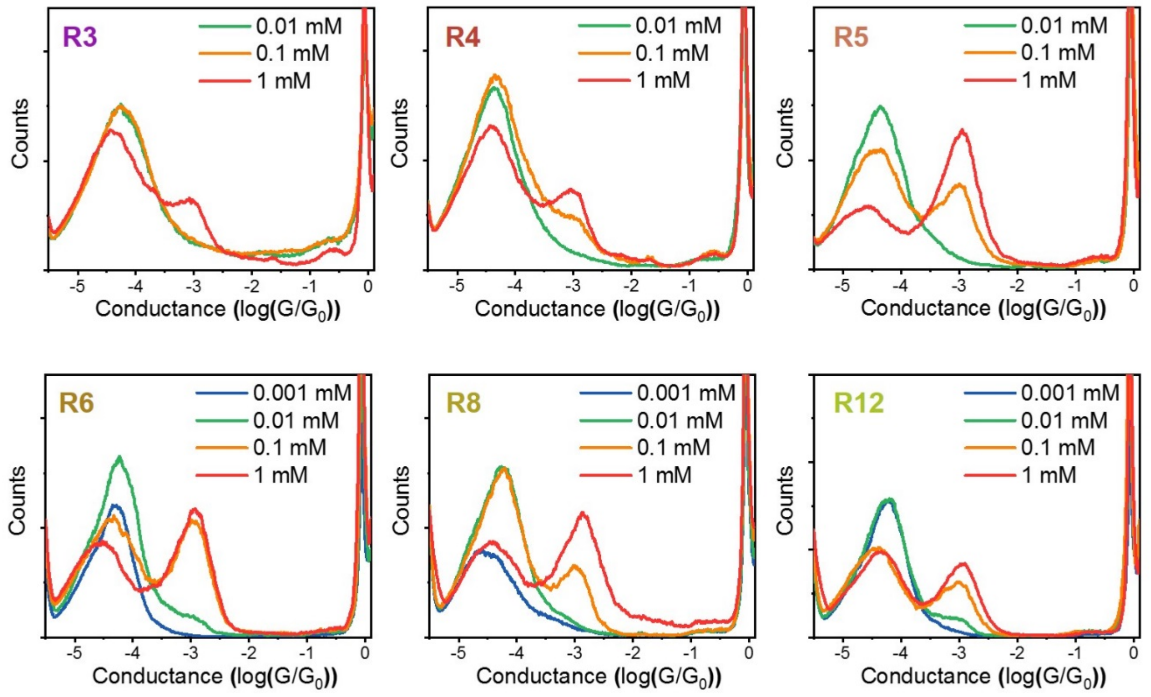

Supplementary Fig. 5 | Concentration dependent study of **R3**, **R4**, **R5**, **R6, R8**, and **R12** (0.25 V).

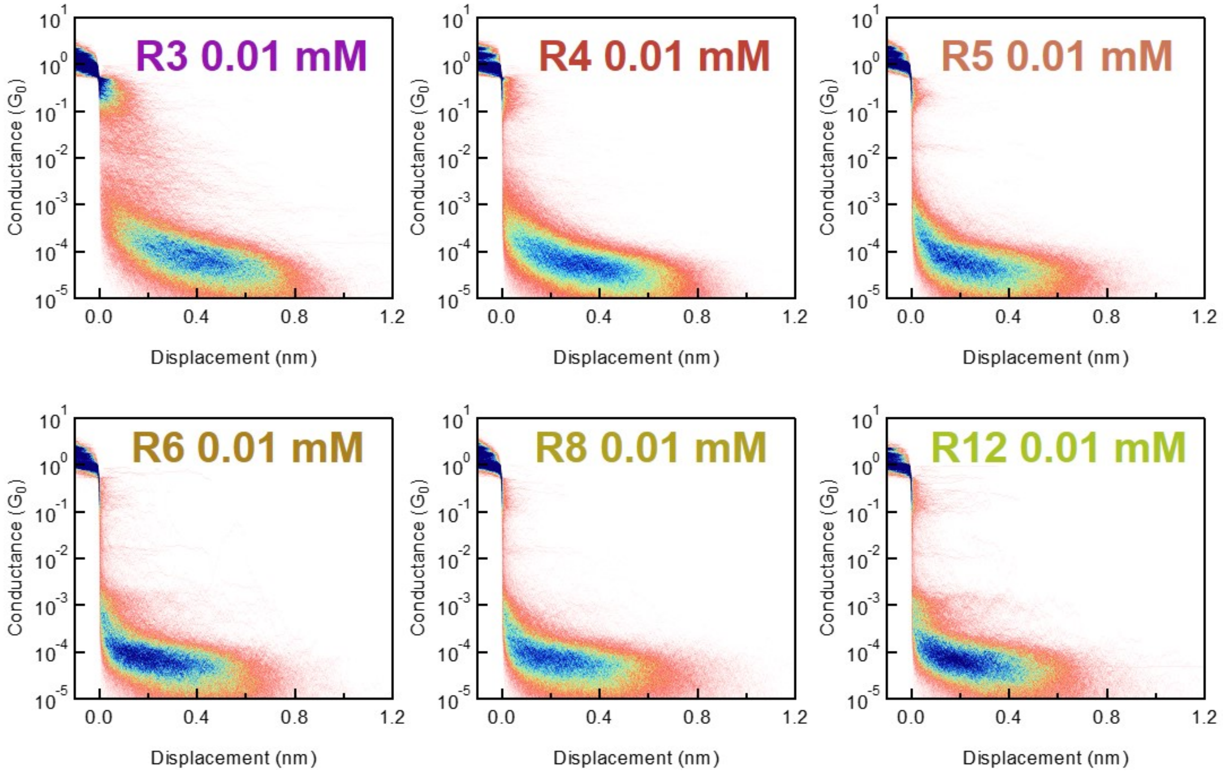

Supplementary Fig. 6 | 2D conductance histograms of 0.01 mM **R3**, **R4**, **R5**, **R6, R8**, and **R12** which does not exhibit the high G state (0.25 V).

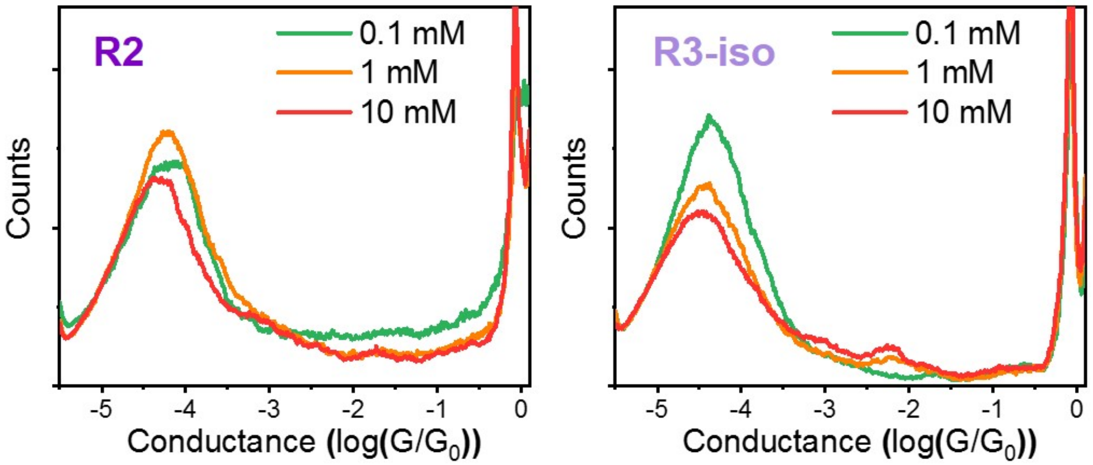

Supplementary Fig. 7 | Concentration dependent study of **R2** and **R3-iPr** (isopropyl side chain) (0.25 V).

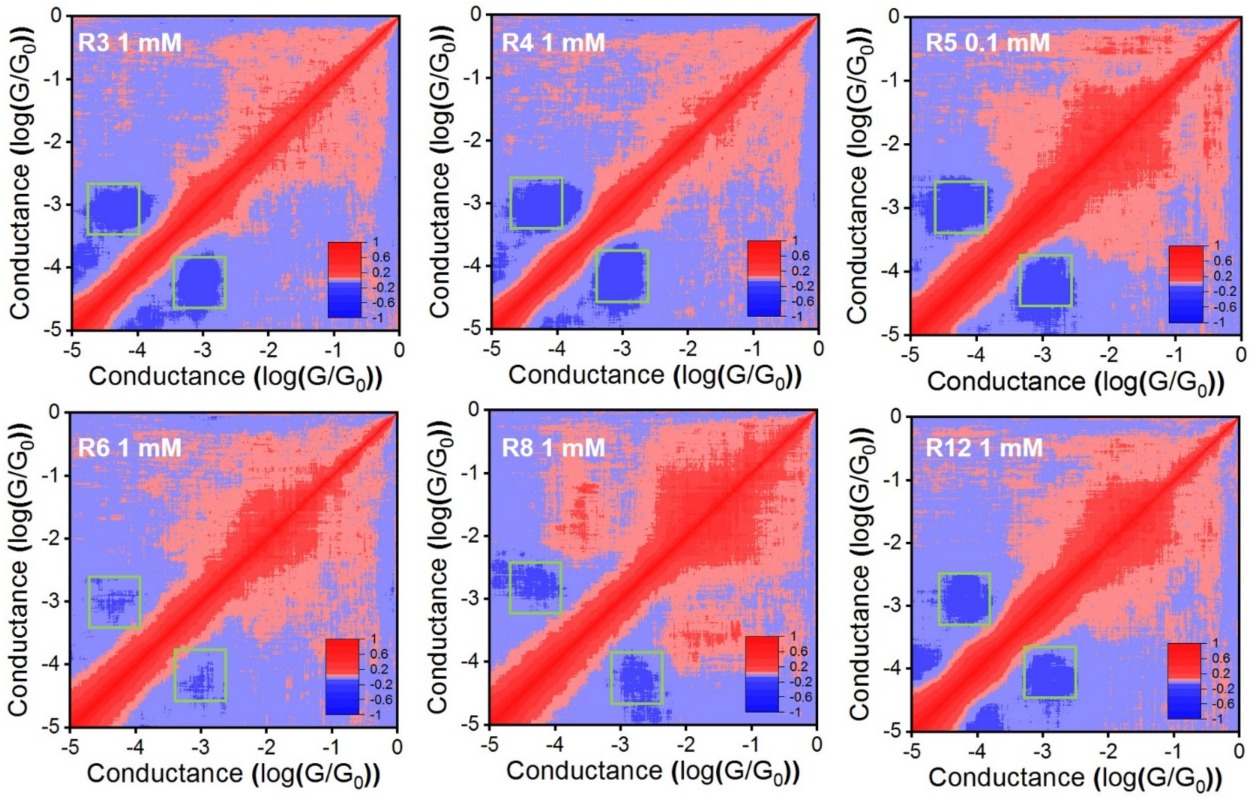

Supplementary Fig. 8 | 2D correlation of **R3**, **R4**, **R5**, **R6, R8**, and **R12** (0.25 V).

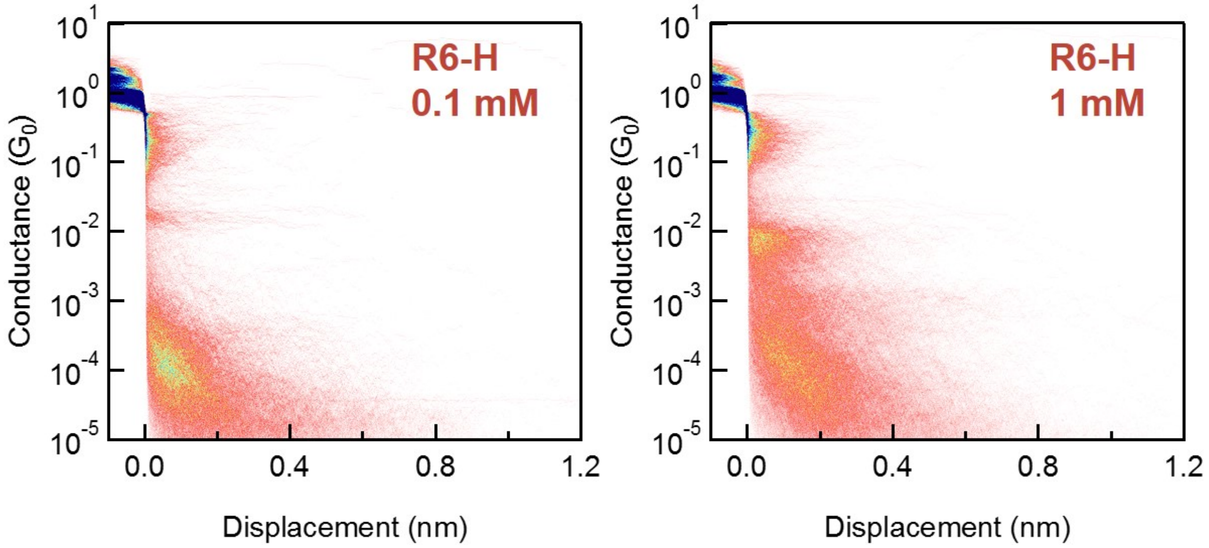

Supplementary Fig. 9 | 2D conductance histograms for **R6-H** at 0.1 mM and 1 mM concentration at 0.25 V.

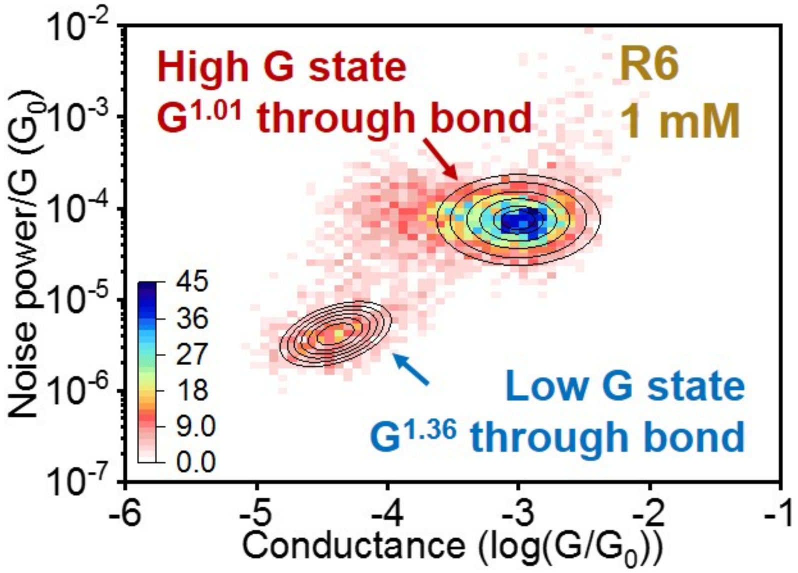

Supplementary Fig. 10 | Flicker noise analysis of 1 mM **R6** which shows the high G state is through bond.

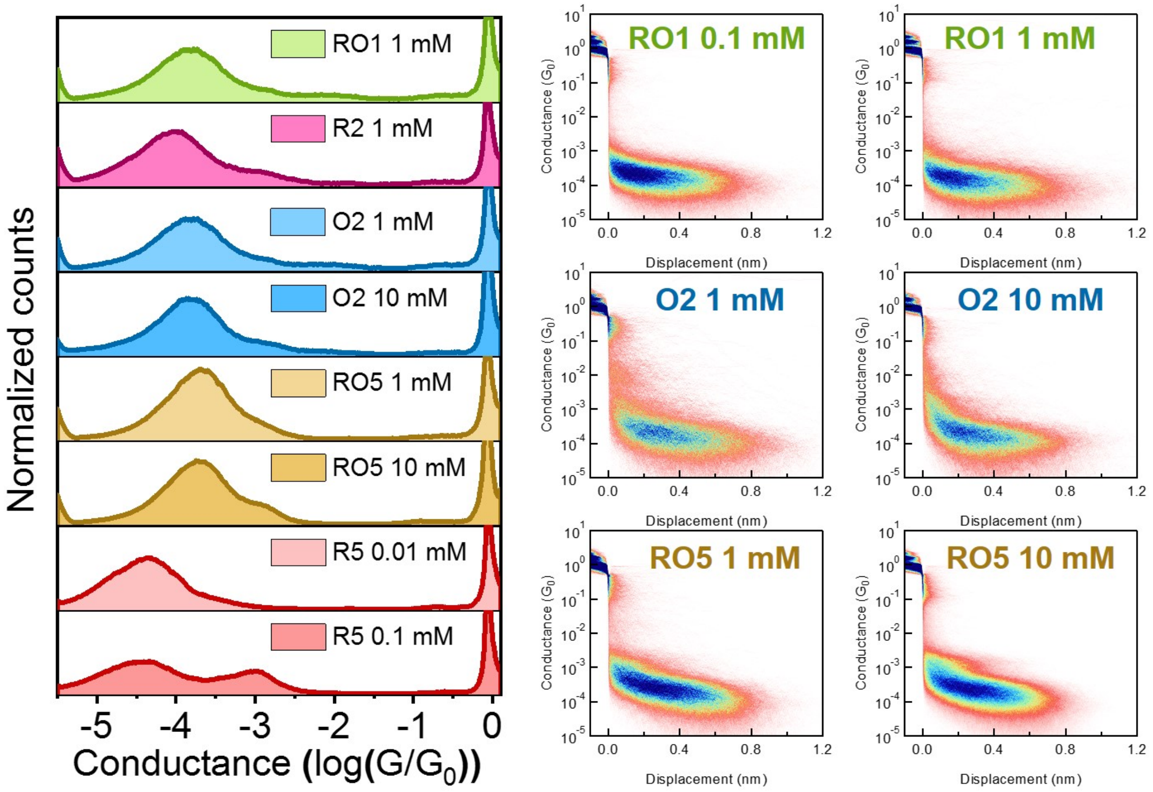

Supplementary Fig. 11 | Single molecule characterizations of **RO1**, **O2**, and **R5** at 0.25 V.

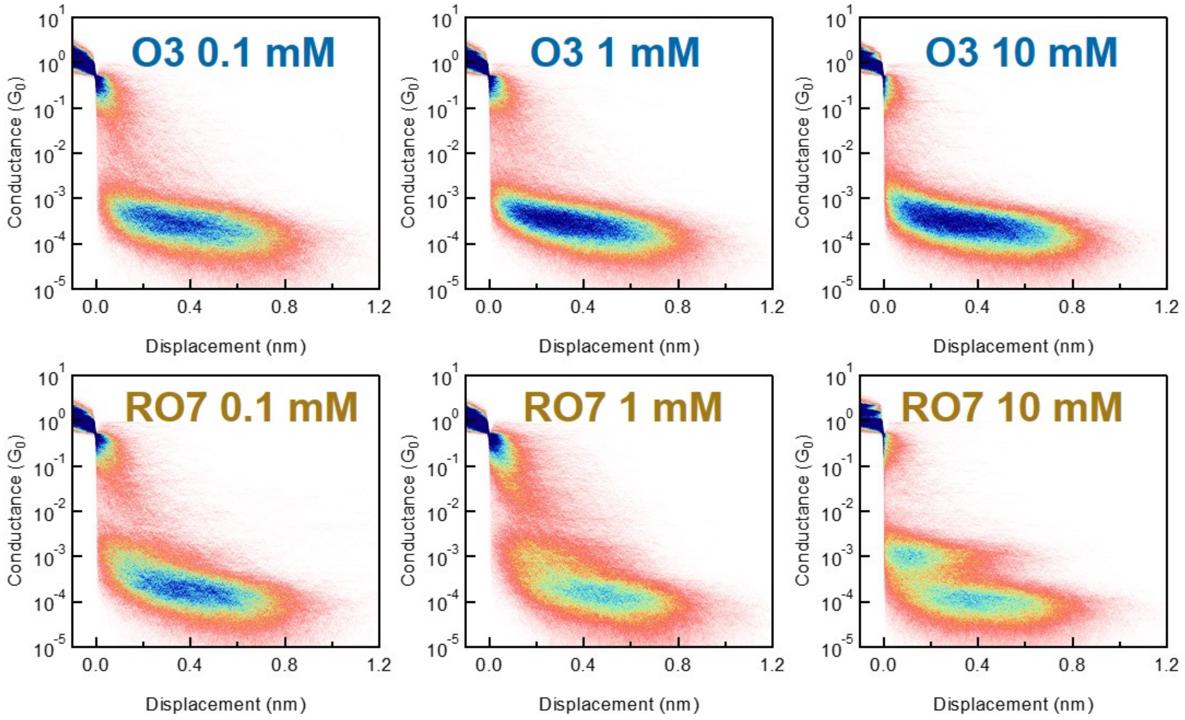

Supplementary Fig. 12 | 2D conductance histograms for **O3** and **RO7** at 0.25 V.

	LUMO (eV)	HOMO (eV)	$Cos^2(\theta)$
RO1	-1.09	-5.39	0.556
R2	-0.74	-5.50	0.001
O2	-1.11	-5.41	0.250
RO5	-1.00	-5.40	0.299
R5	-0.69	-5.57	0.004
O3	-1.11	-5.42	0.280
RO7	-0.99	-5.30	0.291
R8	-0.77	-5.48	0.003

Supplementary Table 1. Molecular properties of terphenyl derivatives with different side chains.

S.4 NMR and UV-vis Dilution Experiment

For the NMR dilution experiment, solutions of **R6** were prepared at concentrations of 10 mM, 1 mM, and 0.1 mM in C_6D_6. No shifting of proton signals corresponding to **R6** upfield or downfield was observed in the ^1H spectra (500 MHz, CB500 instrument with cryoprobe) at these critical concentrations, suggesting no in-solution aggregation phenomena visible on the NMR timescale.

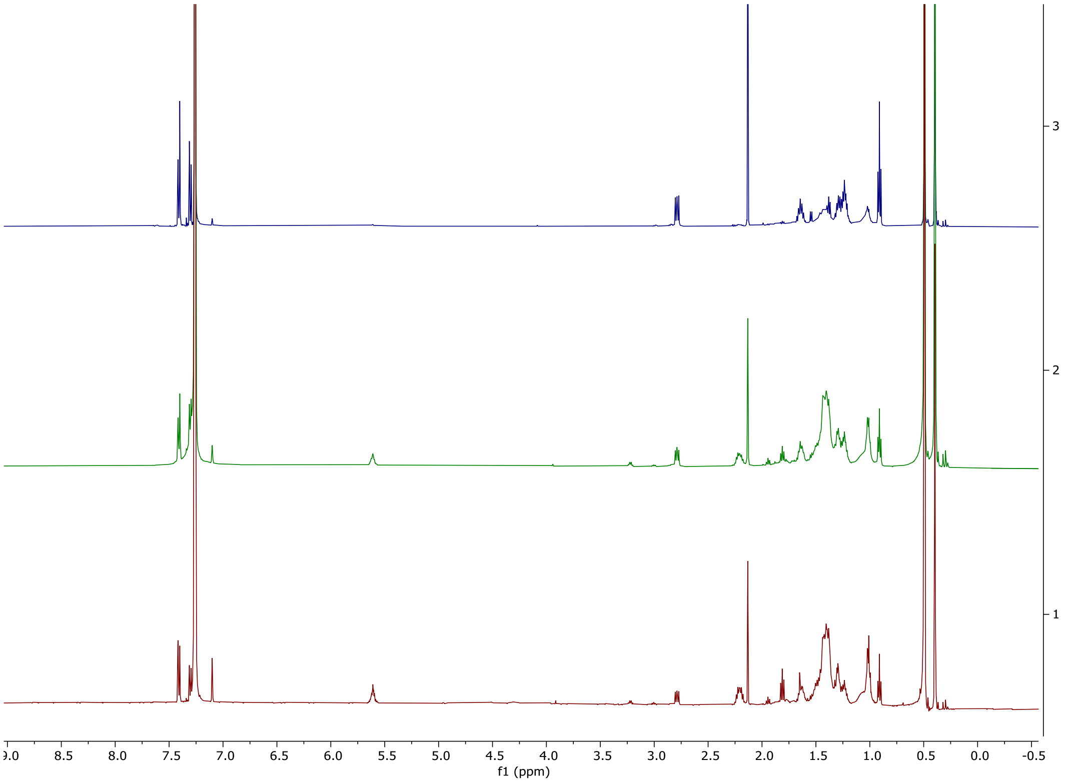

Supplementary Fig. 13 | R6 ^1H spectra (500 MHz, C_6D_6) at 10mM (top), 1 mM (middle), and 0.1 mM (bottom)

For UV-Vis dilution experiments, we studied R6 at 0.1 mM, 0.05 mM, and 0.01 mM in 1,2,4-trichlorobenzene using a Nanodrop UV-Vis instrument with a quartz cuvette (10 mm path length). In general, concentrations larger than 0.5 mM saturated the UV/Vis detector and did not provide accurate UV/Vis measurements. From these data, we observe no clear differences in the UV/Vis spectra between alkyl chain containing terphenyl molecules that display the high conductance mode in the STM-BJ experiments and those that do not. In particular, we observe no clear aggregation-related red-shifting between 0.01 mM and 0.1 mM, and there is no apparent spectral shifting occurs above the background absorption of the solvent. However, we note that the spectral cutoff (background absorption) for 1,2,4-trichlorobenzene (TCB), the solvent used in this experiment and the STM-BJ experiments, is quite high (~304 nm) and may obscure absorbance features of the terphenyl molecules below this wavelength. We believe that these results are consistent with the NMR dilution experiments, which provides evidence against in-solution aggregation driving conductance behavior.

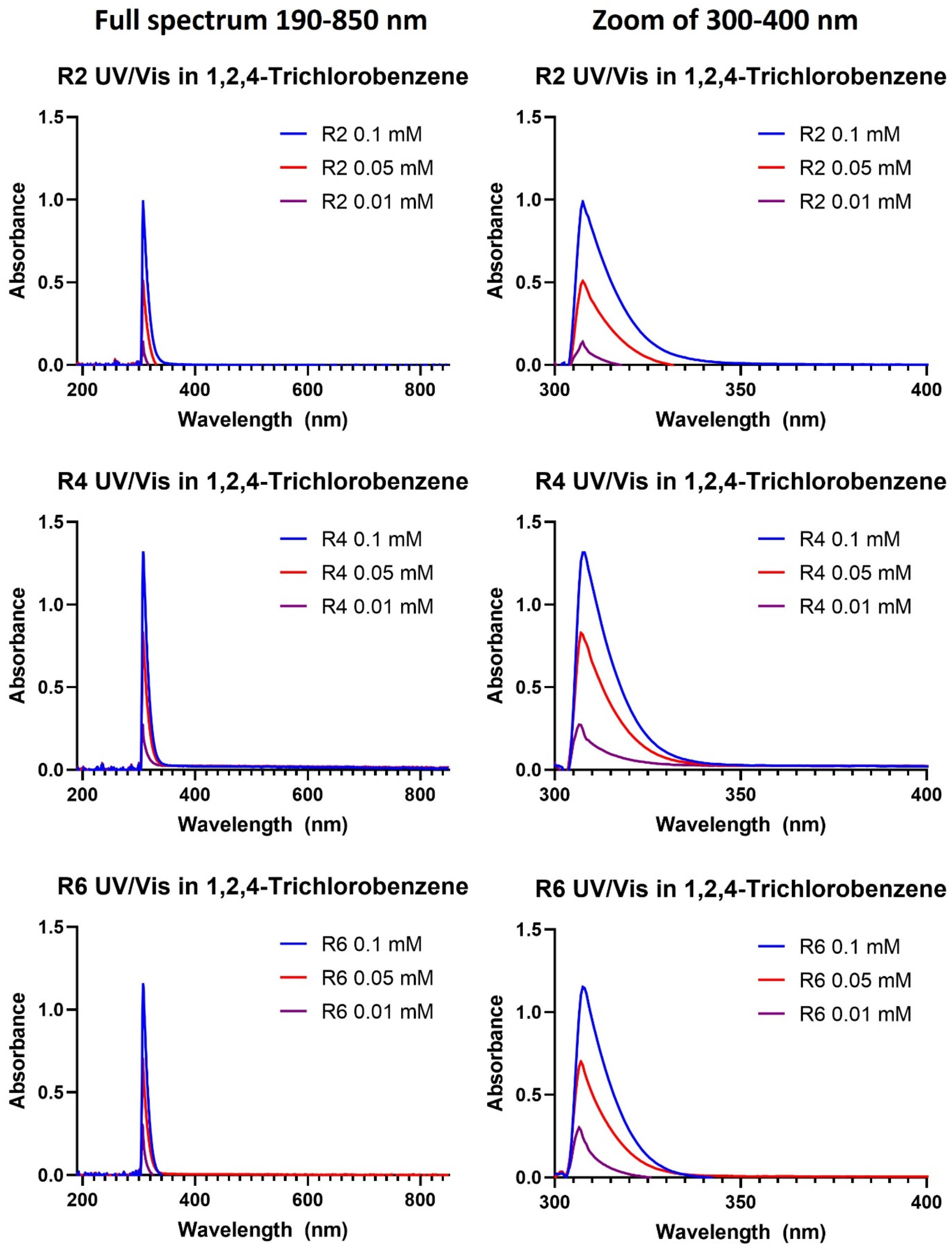

Supplementary Fig. 14 | UV-vis spectra for **R2**, **R4**, and **R6** at 0.01 mM, 0.05 mM, and 0.1 mM.

S.5 Analytical kinetic model

We developed an analytical model for the adsorption of terphenyl molecules onto the gold surface based on the Langmuir adsorption model. In keeping with the Langmuir model we make several assumptions about the system.

1. Flat, uniform surface. We assume that the gold surface contains a fixed number of sites and adsorption on all sites is energetically equivalent.
2. Mono-layer coverage. We assume each site can hold, at most, one molecule.
3. No surface interaction. We assume surface bound molecules do not interact with other surface bound molecules.
4. No surface diffusion. We assume that adsorbed molecules are fixed to their original adsorption site and do not freely diffuse on the surface.
5. Fast solution diffusion. We assume that rate of diffusion in solution is fast compared to the rate of adsorption onto the surface. Thus, we take the solution concentration to be spatially uniform.
6. Constant Solution concentration. We assume that the solution concentration of molecules is much greater than the number of surface sites. Thus, we take the solution concentration to be uniform in time.

We consider a flat, gold surface with N total sites. We take as our species of interest the terphenyl molecule species A. Species A can be adsorbed onto the gold surface in two separate configurations. First, we have the standing up, or low conductance configuration A^*. We also have the lying down, or high conductance state $A^\dagger$.

We model the transition between these three states as a reversable two-step adsorption process. In the first step a molecule in solution interacts with an empty surface site, S to form an adsorbed molecule in the standing up state A^*. This step proceeds with forward constant k_1 and reverse constant k_{-1}.

$$A + S \rightleftharpoons A^* \tag{1}$$

For the second step, we consider an adsorbed molecule A^* which interacts with a molecule in solution to planarize and adopt a lying down confirmation. This is modeled as a second order reversible reaction with forward constant k_2 and reverse constant k_{-2}.

$$A + A^* \rightleftharpoons A^\dagger + A \tag{2}$$

Combining these, we can formulate differential equations describing the time evolution of the surface concentrations of vacant sites [S], sites occupied by adsorbed, standing up molecule [A^*], and sites occupied by adsorbed, lying down molecule [$A^\dagger$].

$$\frac{d[S]}{dt} = -k_1[S]C_A + k_{-1}[A^*] \tag{3}$$

$$\frac{d[A^*]}{dt} = k_1[S]C_A - k_{-1}[A^*] - k_2[A^*]C_A + k_{-2}[A^\dagger] \tag{4}$$

$$\frac{d[A^\dagger]}{dt} = k_2[A^*]C_A - k_{-2}[A^\dagger] \tag{5}$$

In our experiments, we add our sample solution to a freshly cleaned gold surface with no molecules adsorbed. This yields the following initial conditions.

$$[S](t = 0) = N \quad (6)$$

$$[A^*](t = 0) = 0 \quad (7)$$

$$[A^\dagger](t = 0) = 0 \quad (8)$$

This system of differential equations was solved numerically using the Scipy.optimize package in Python. Solution was iterated over values of dimensionless solution concentration C_A to yield plots of surface coverage of A* and $A^\dagger$ vs. C_A (**Supplementary Fig. 14**). This process was repeated for varying values of $K_2 = (k_2/k_{-2})$ for fixed $K_1 = (k_1/k_{-1})$ in order to analyze the effect of changing the relative adsorption energy between the two states. Using this model, we can see that, at low concentration, the standing up state dominates while the fraction of molecules in the lying down state is negligible. However, as solution concentration increases past a critical value, the ratio of molecules in the standing up state rapidly increases and eventually becomes the dominant state at ultrahigh concentrations. This is consistent with experimental results seen in concentration dependent conductance studies of **R3-R12**

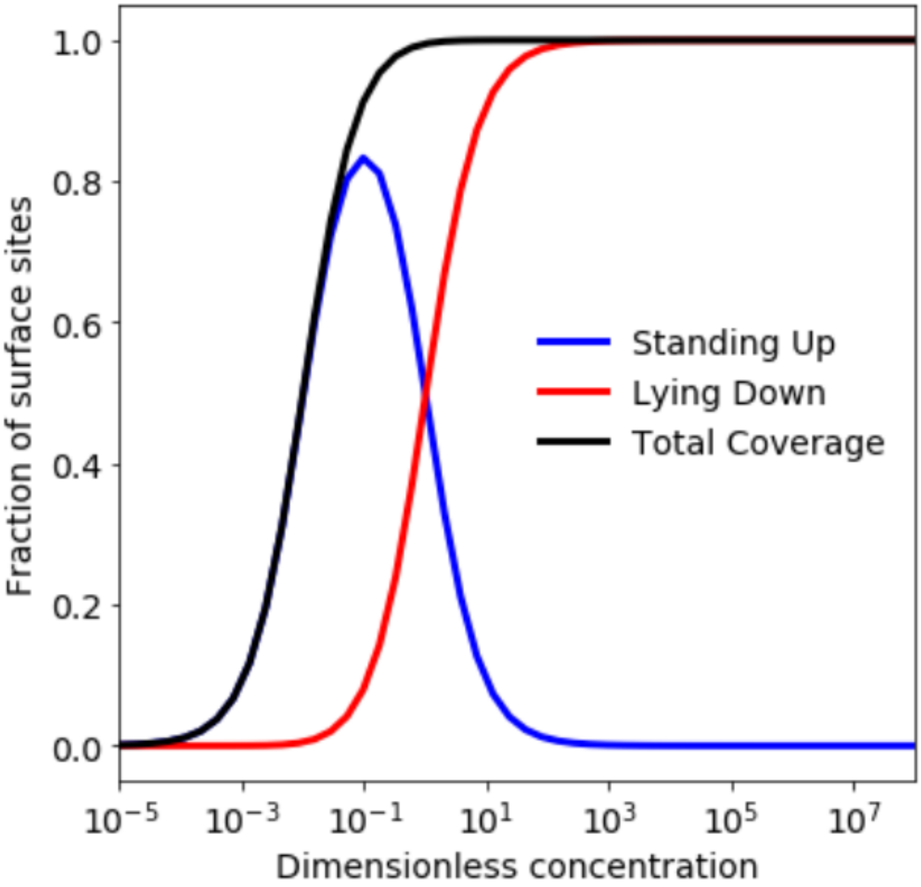

Supplementary Fig. 15 | Plot of surface coverage as a function of solution concentration from analytical model for representative values of $K_1 = 100$ and $K_2 = 1$

S.6 NMR Spectra

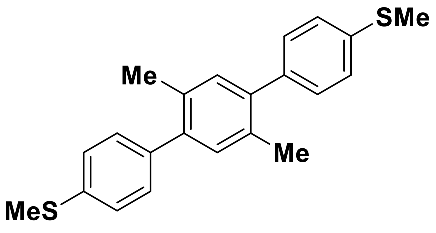

R1

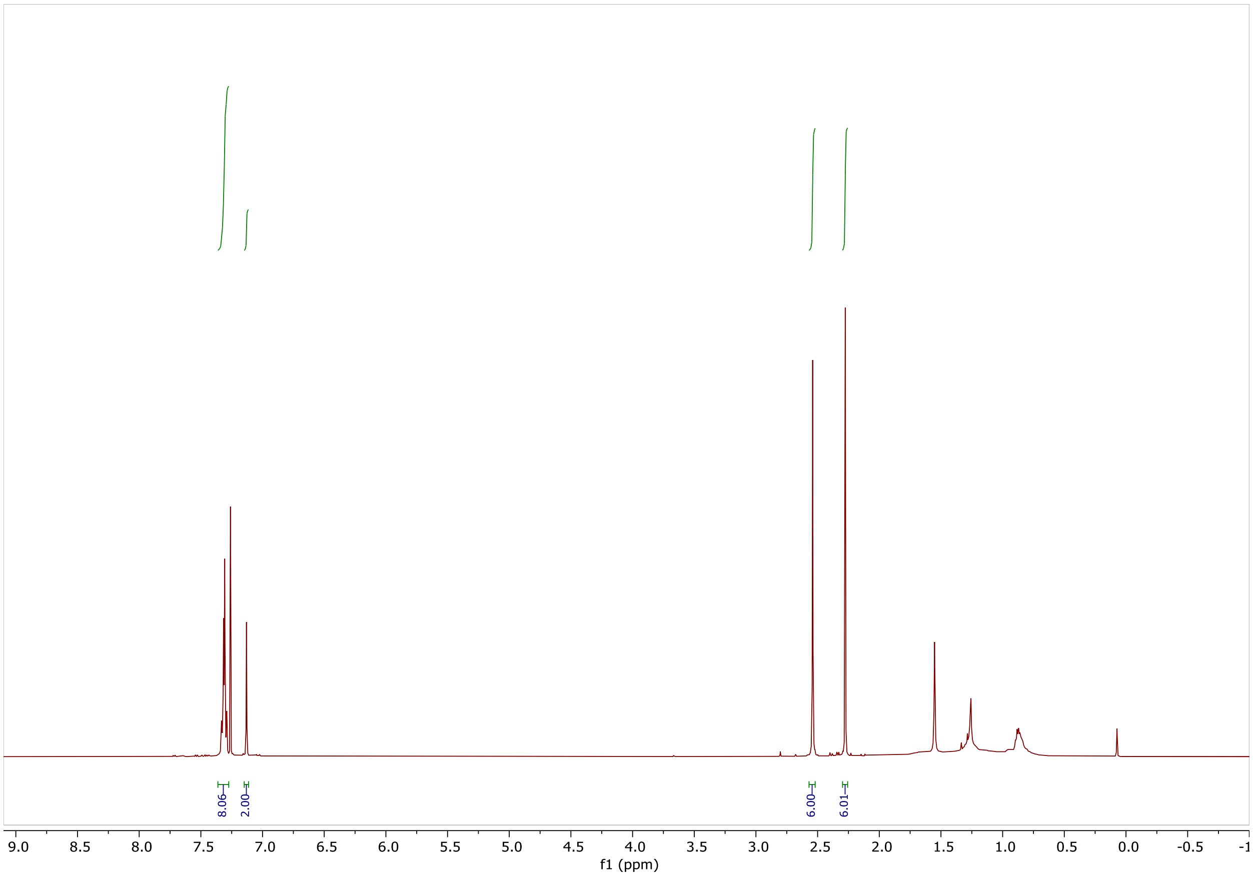

Supplementary Figure 16. ^{1}H-NMR spectrum (500 MHz, $CDCl_3$) for **R1**.

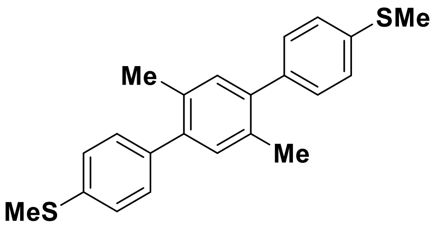

R1

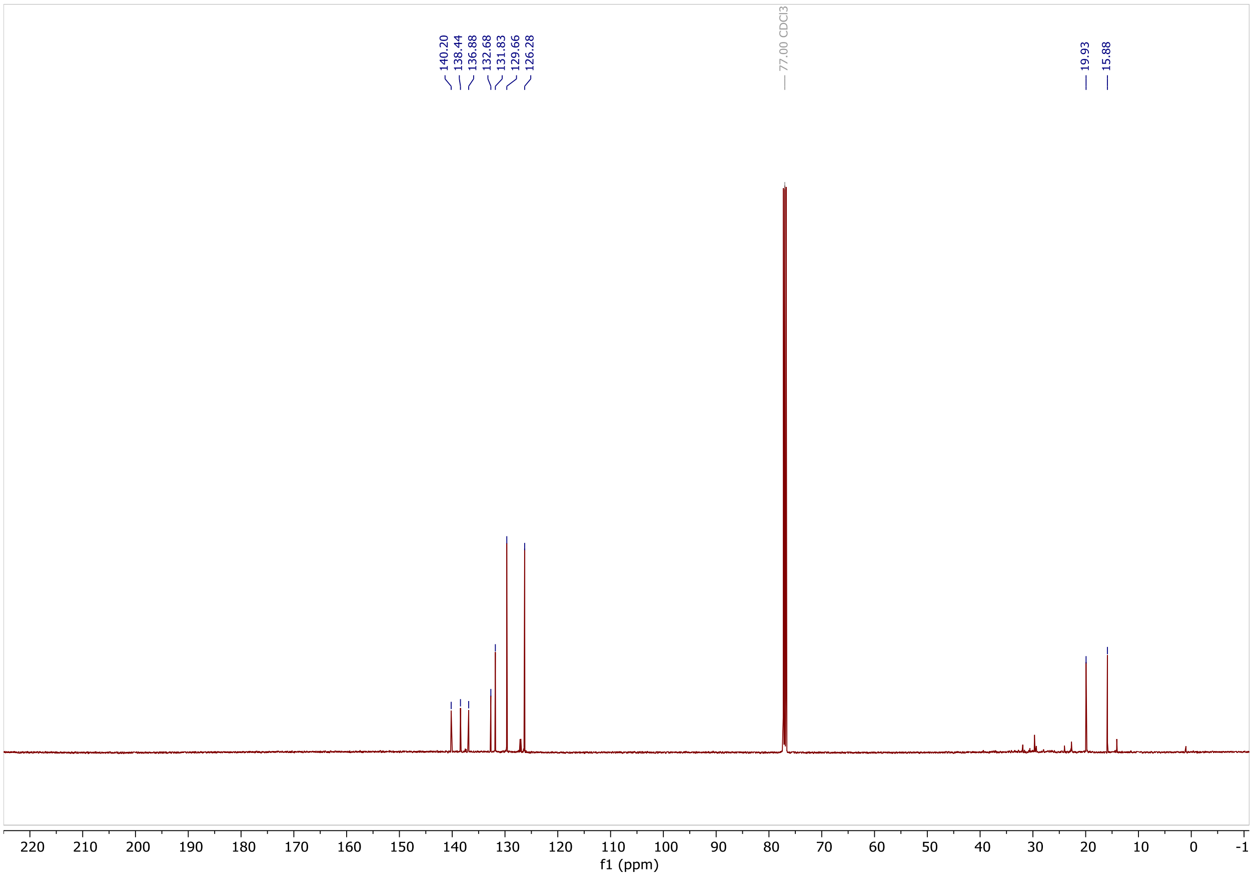

Supplementary Figure 17. ^{13}C-NMR spectrum (126 MHz, $CDCl_3$) for **R1**.

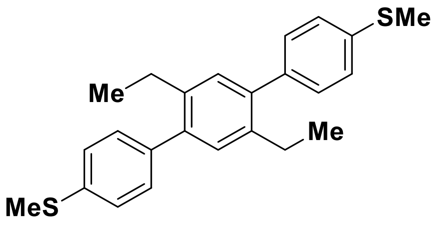

R2

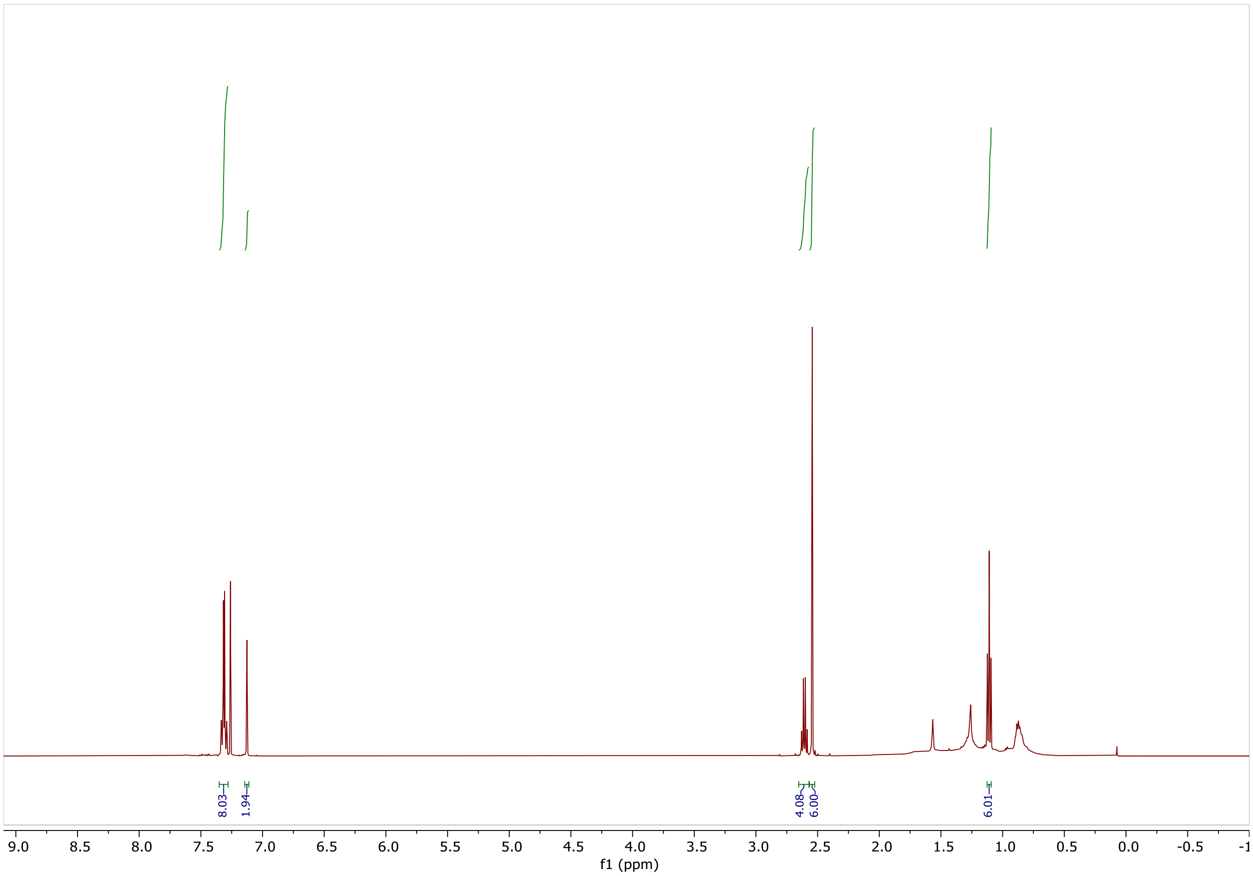

Supplementary Figure 18. ^{1}H-NMR spectrum (500 MHz, $CDCl_3$) for **R2**.

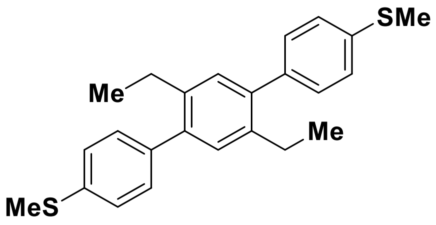

R2

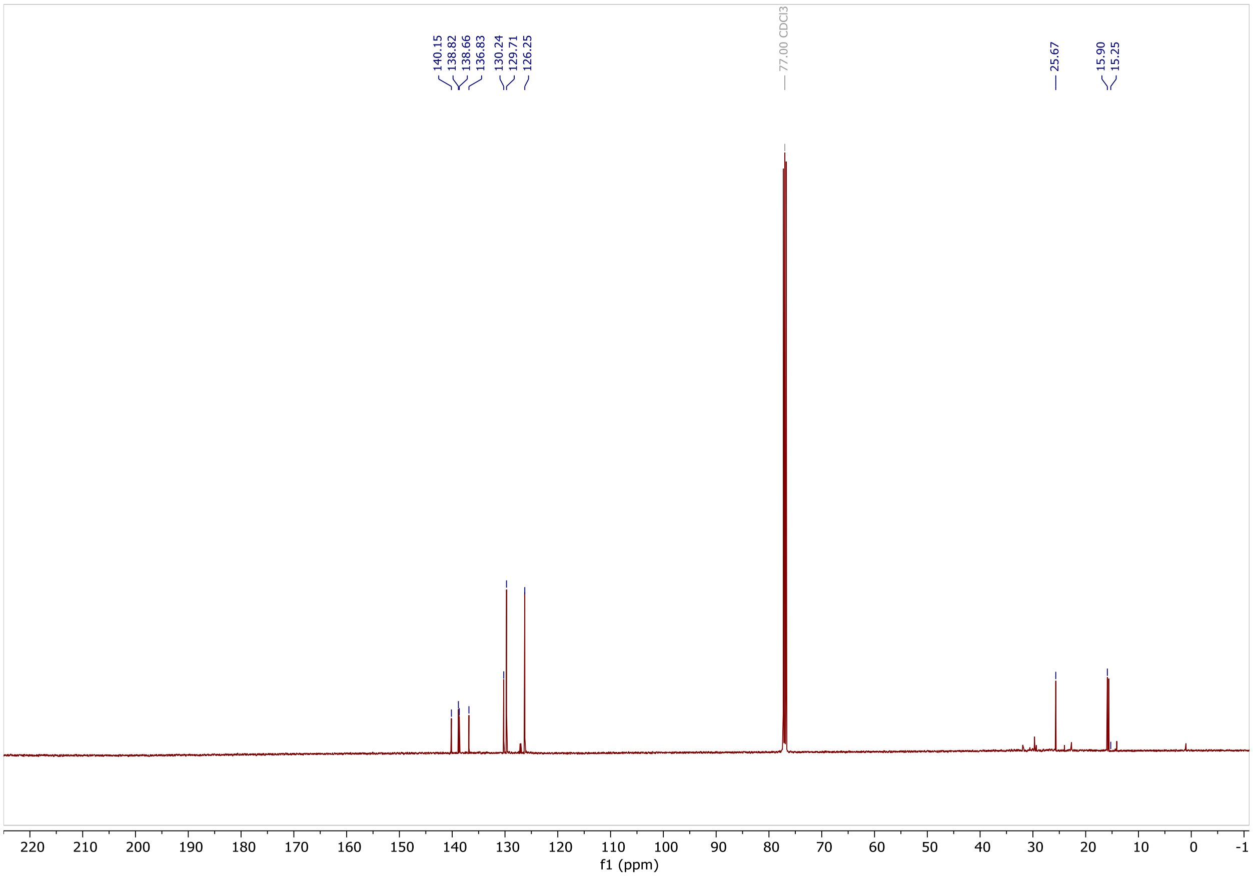

Supplementary Figure 19. ^{13}C-NMR spectrum (500 MHz, $CDCl_3$) for **R2**.

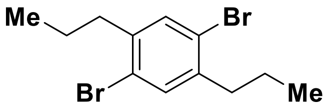

SI-1

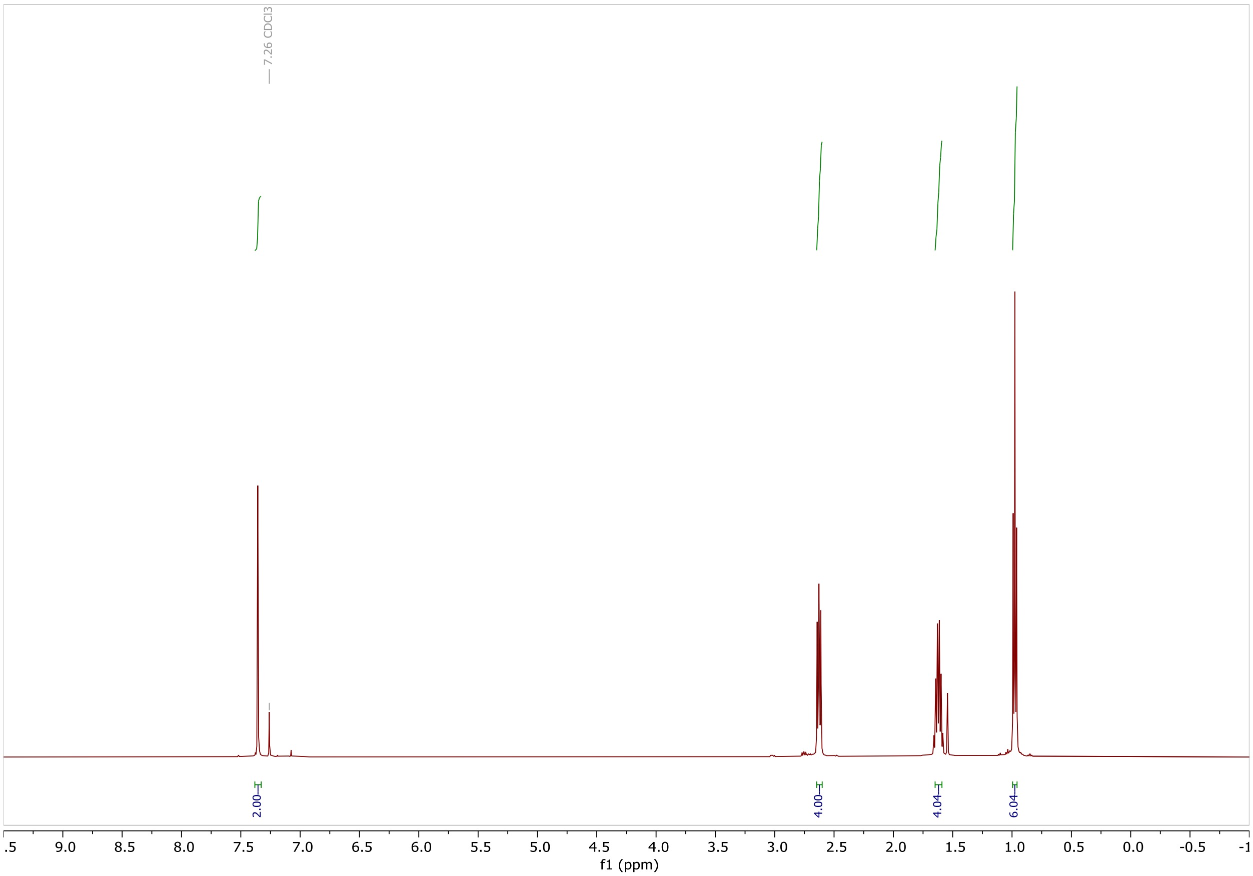

Supplementary Figure 20. ^{1}H-NMR spectrum (500 MHz, $CDCl_3$) for **SI-1**.

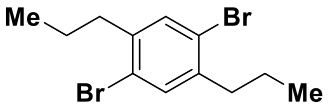

SI-1

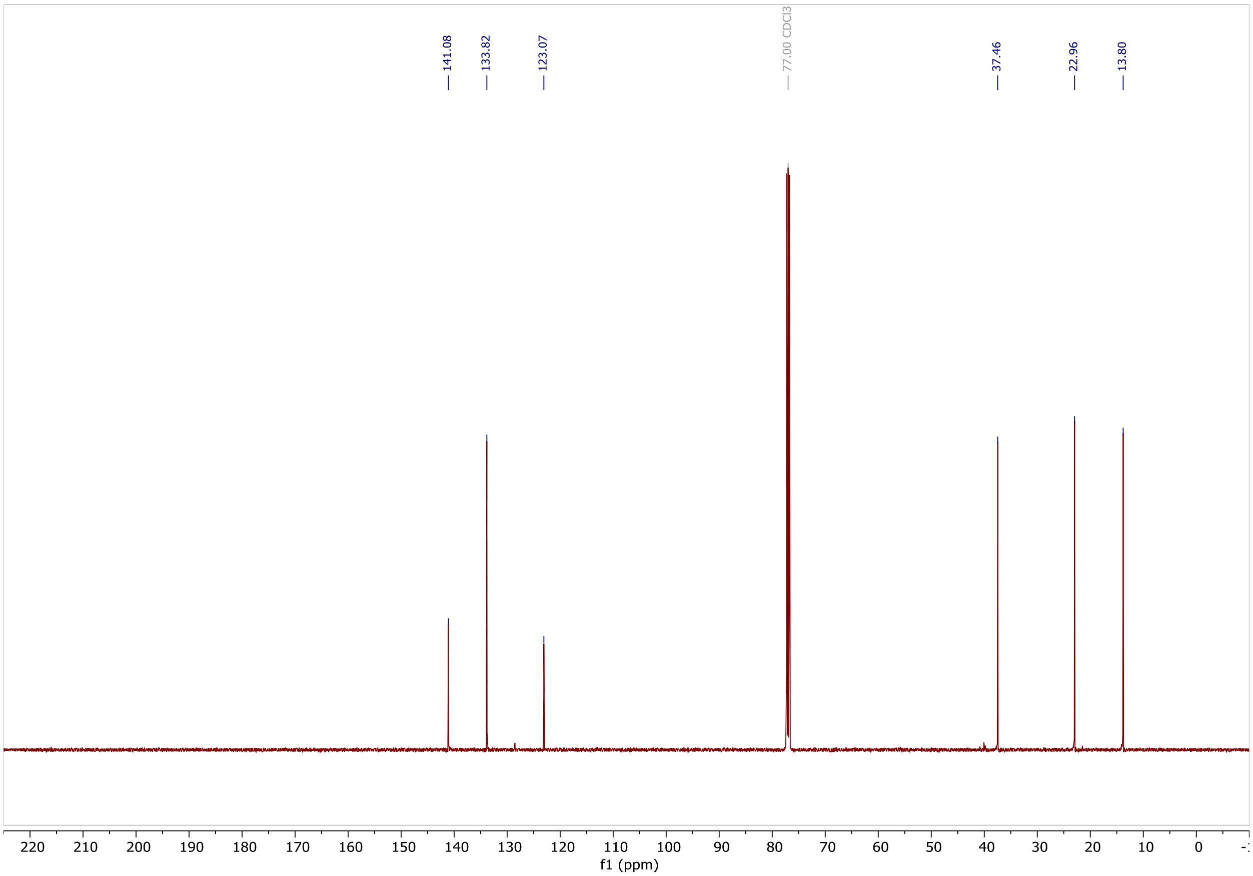

Supplementary Figure 21. ^{13}C-NMR spectrum (126 MHz, $CDCl_3$) for **SI-1**.

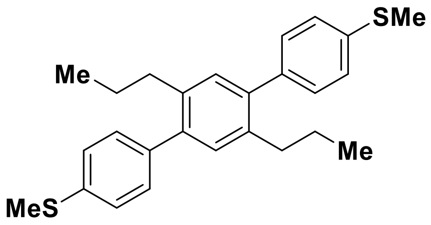

R3

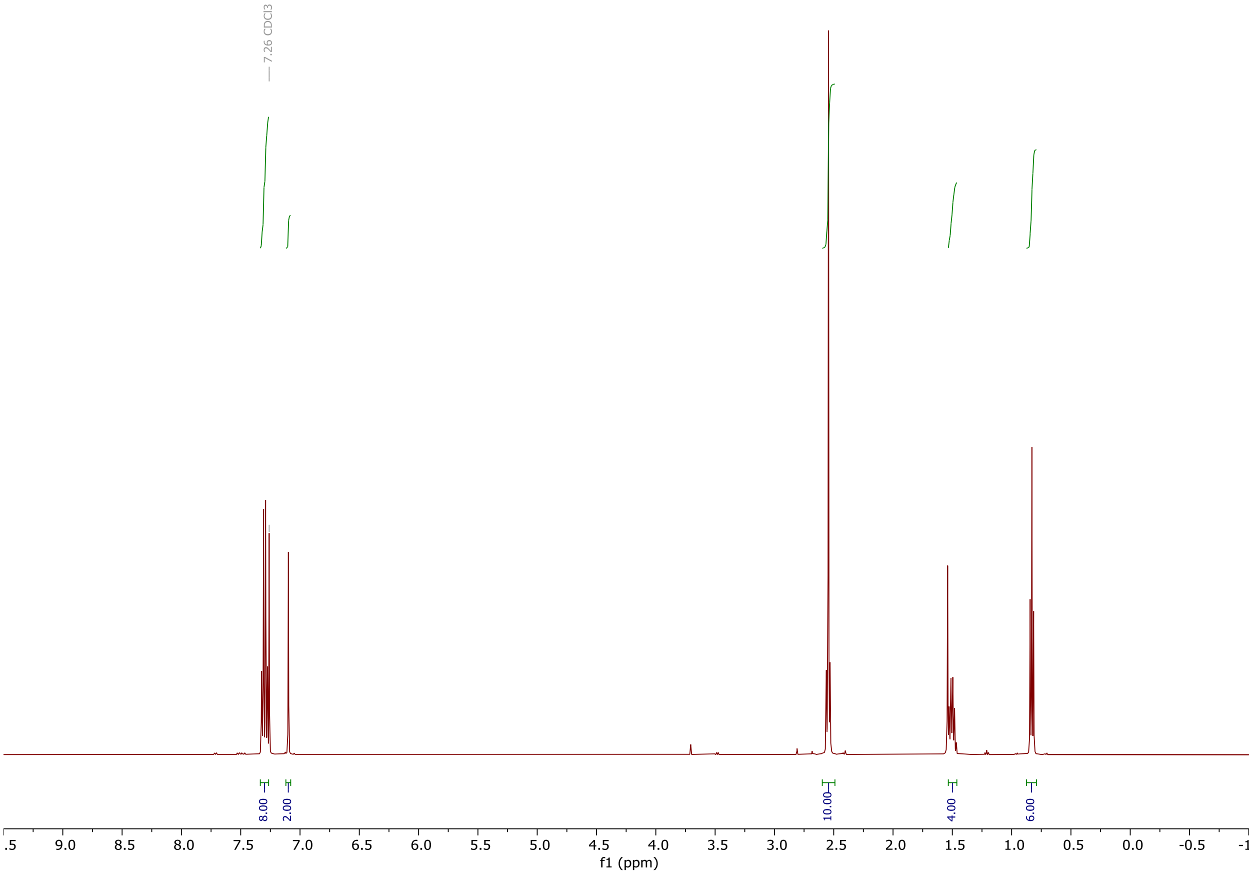

Supplementary Figure 22. ^{1}H-NMR spectrum (500 MHz, $CDCl_3$) for **R3**.

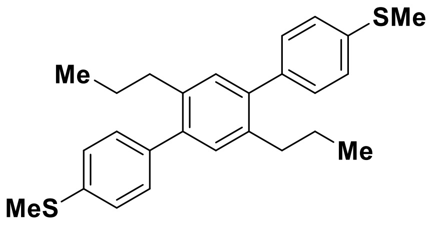

R3

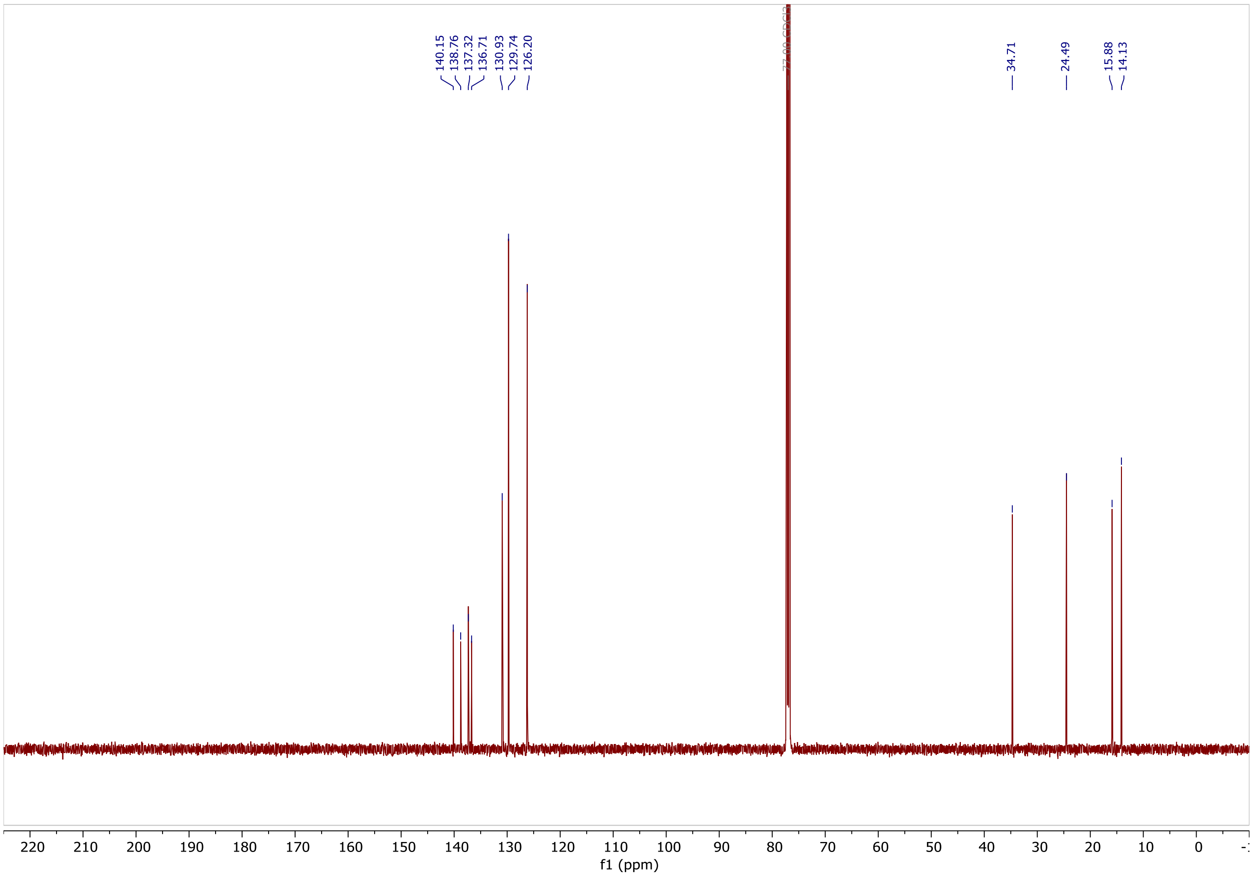

Supplementary Figure 23. ^{13}C-NMR spectrum (126 MHz, $CDCl_3$) for **R3**.

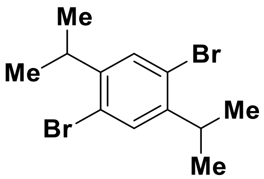

SI-2

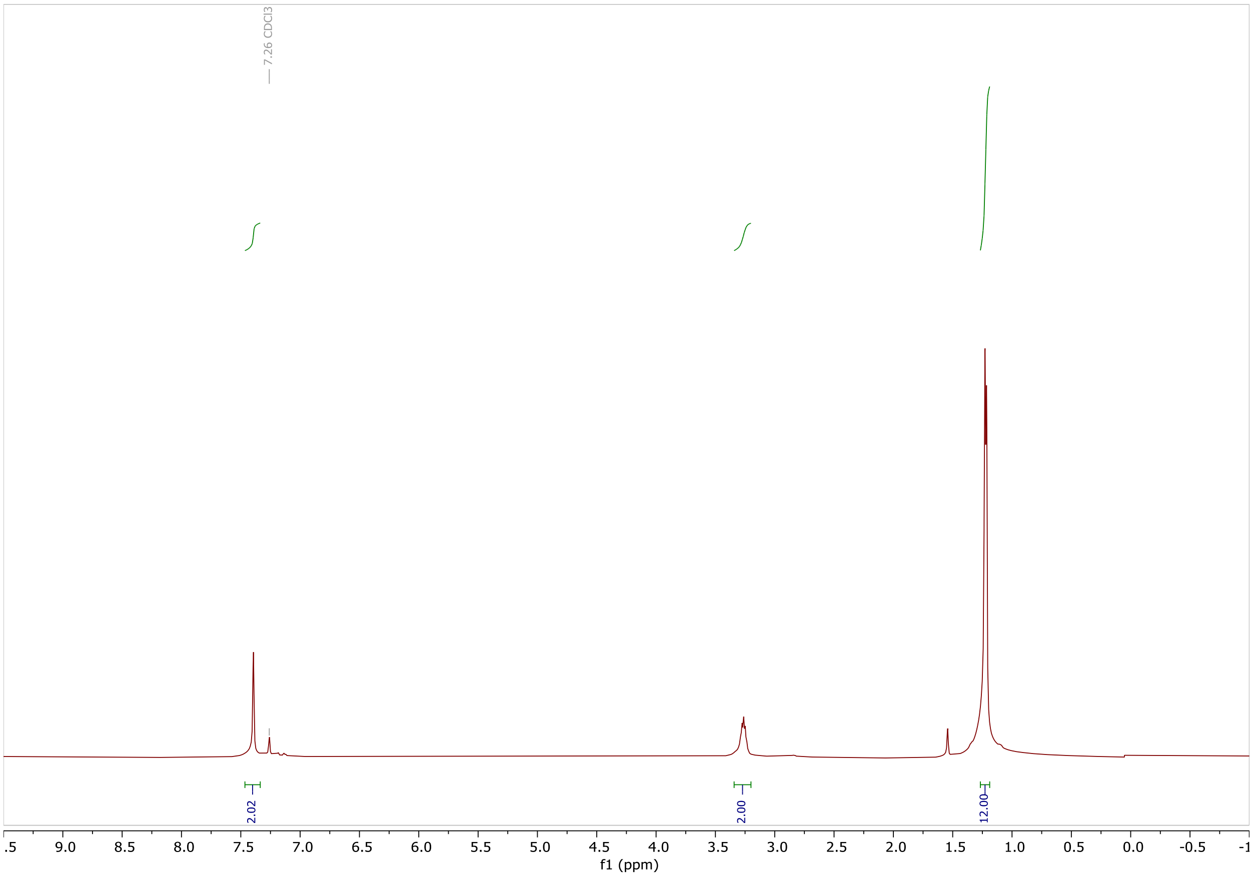

Supplementary Figure 24. ^{1}H-NMR spectrum (500 MHz, $CDCl_3$) for **SI-2**.

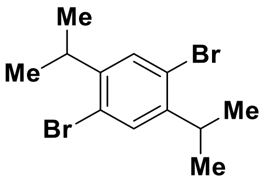

SI-2

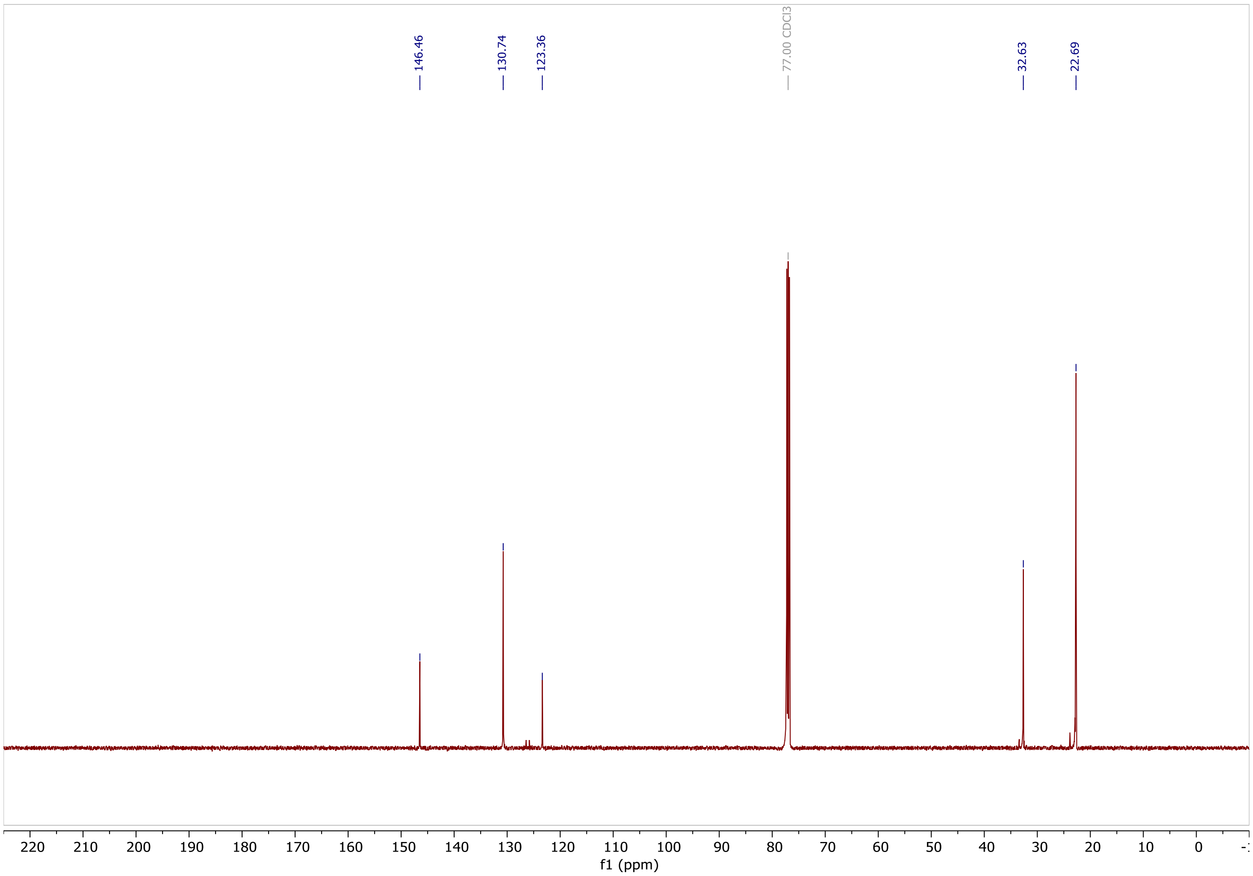

Supplementary Figure 25. ^{13}C-NMR spectrum (126 MHz, $CDCl_3$) for **SI-2**.

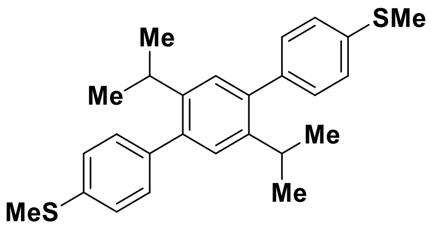

R3-iPr

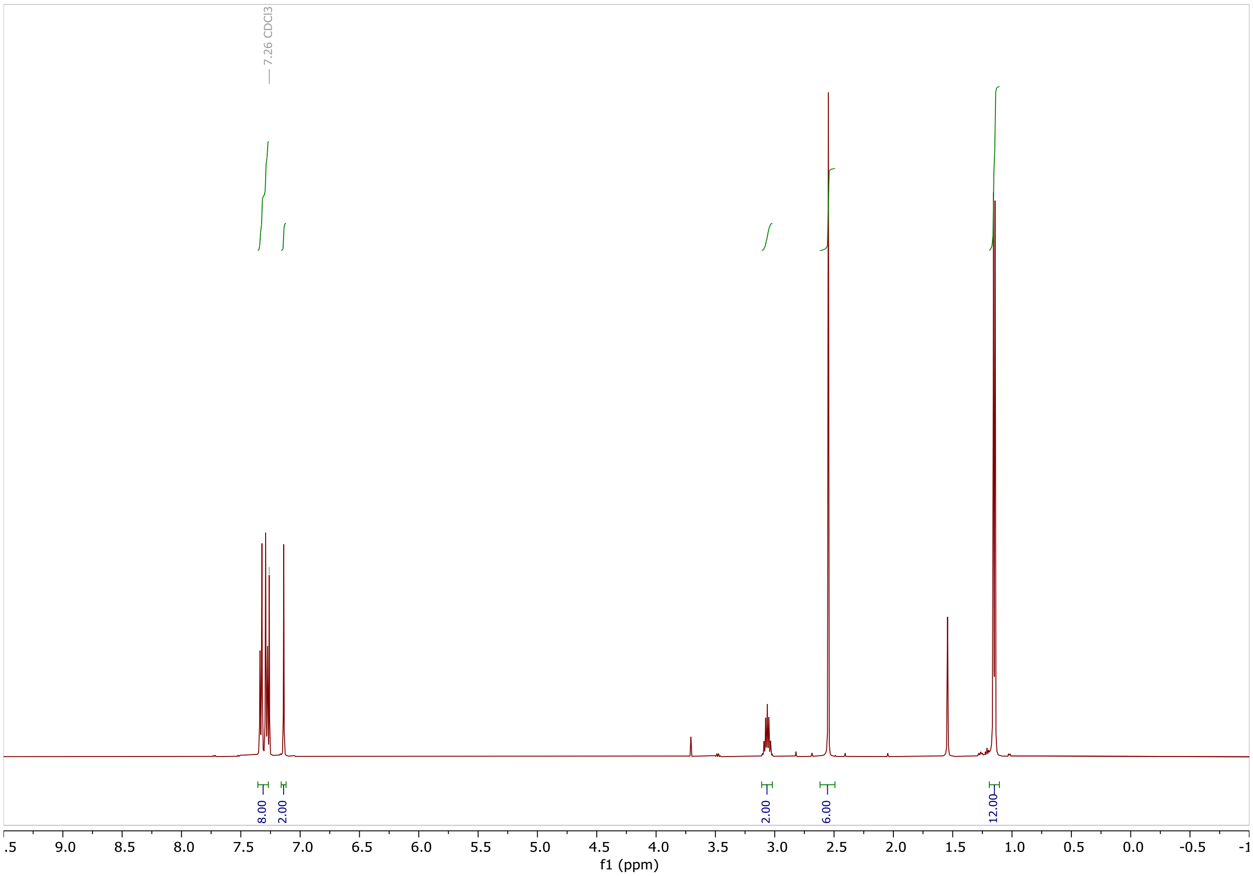

Supplementary Figure 26. ^{1}H-NMR spectrum (500 MHz, $CDCl_3$) for **R3-*i*Pr**.

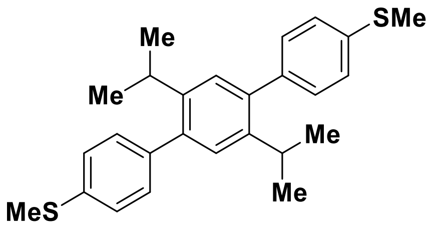

R3-iPr

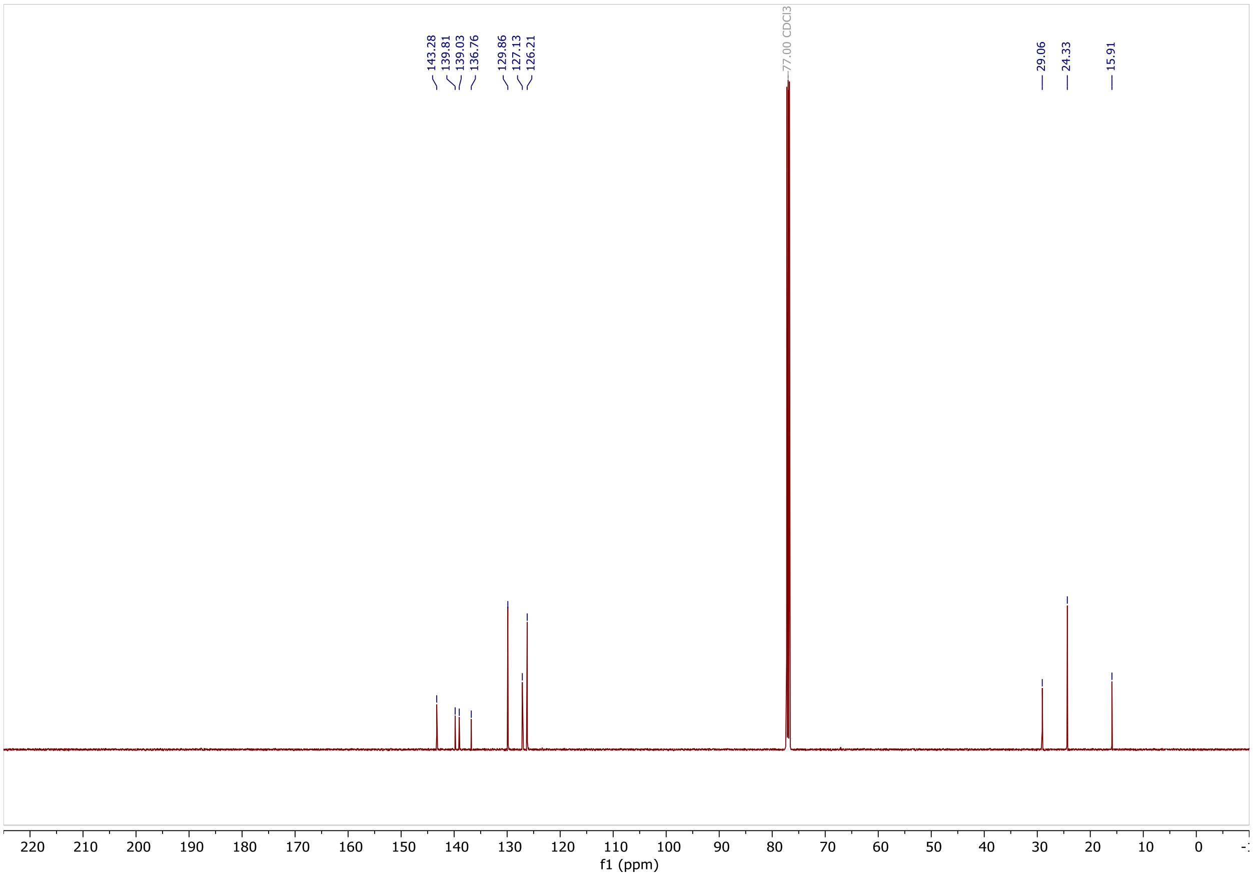

Supplementary Figure 27. ^{13}C-NMR spectrum (126 MHz, $CDCl_3$) for **R3-*i*Pr**.

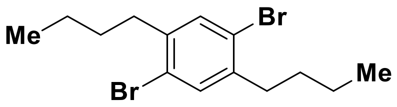

SI-3

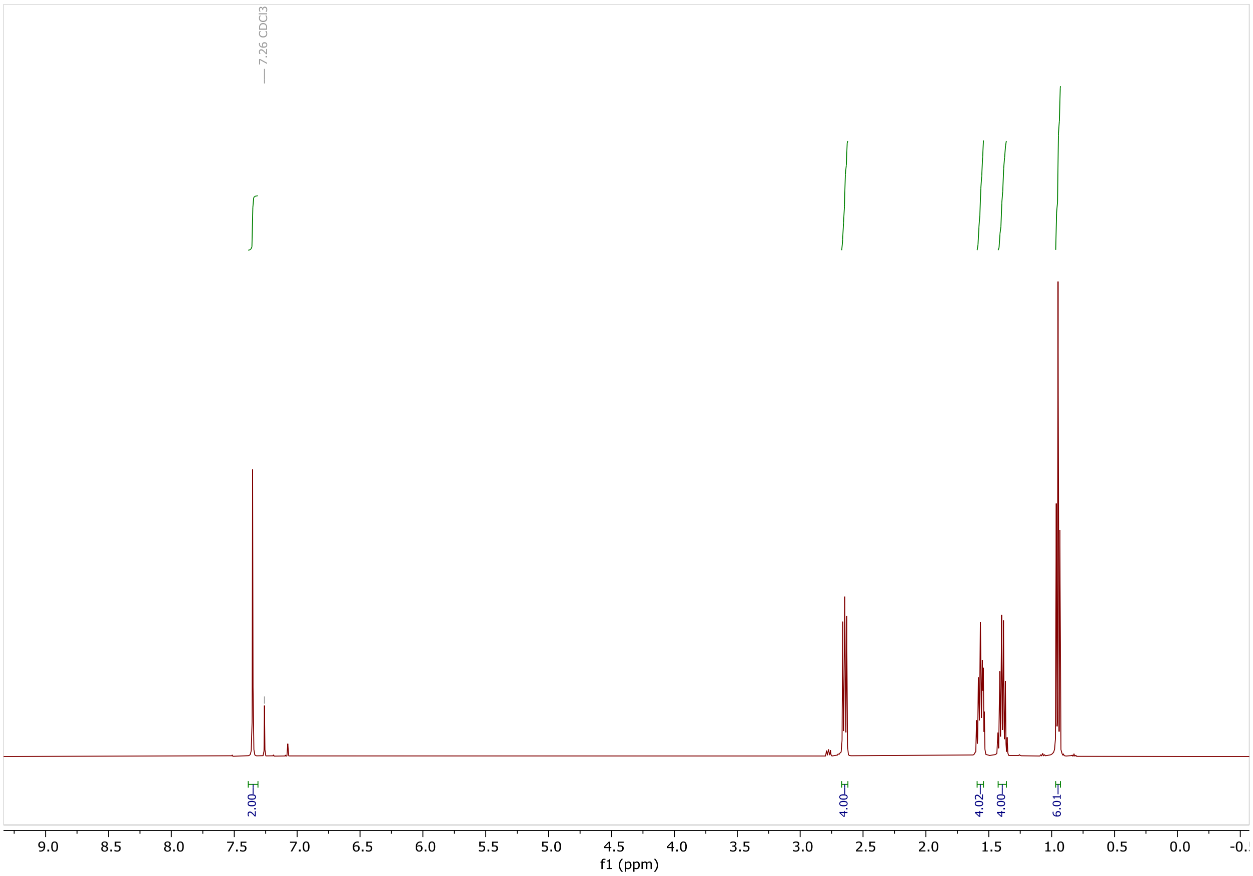

Supplementary Figure 28. ^{1}H-NMR spectrum (500 MHz, $CDCl_3$) for **SI-3**.

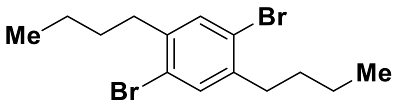

SI-3

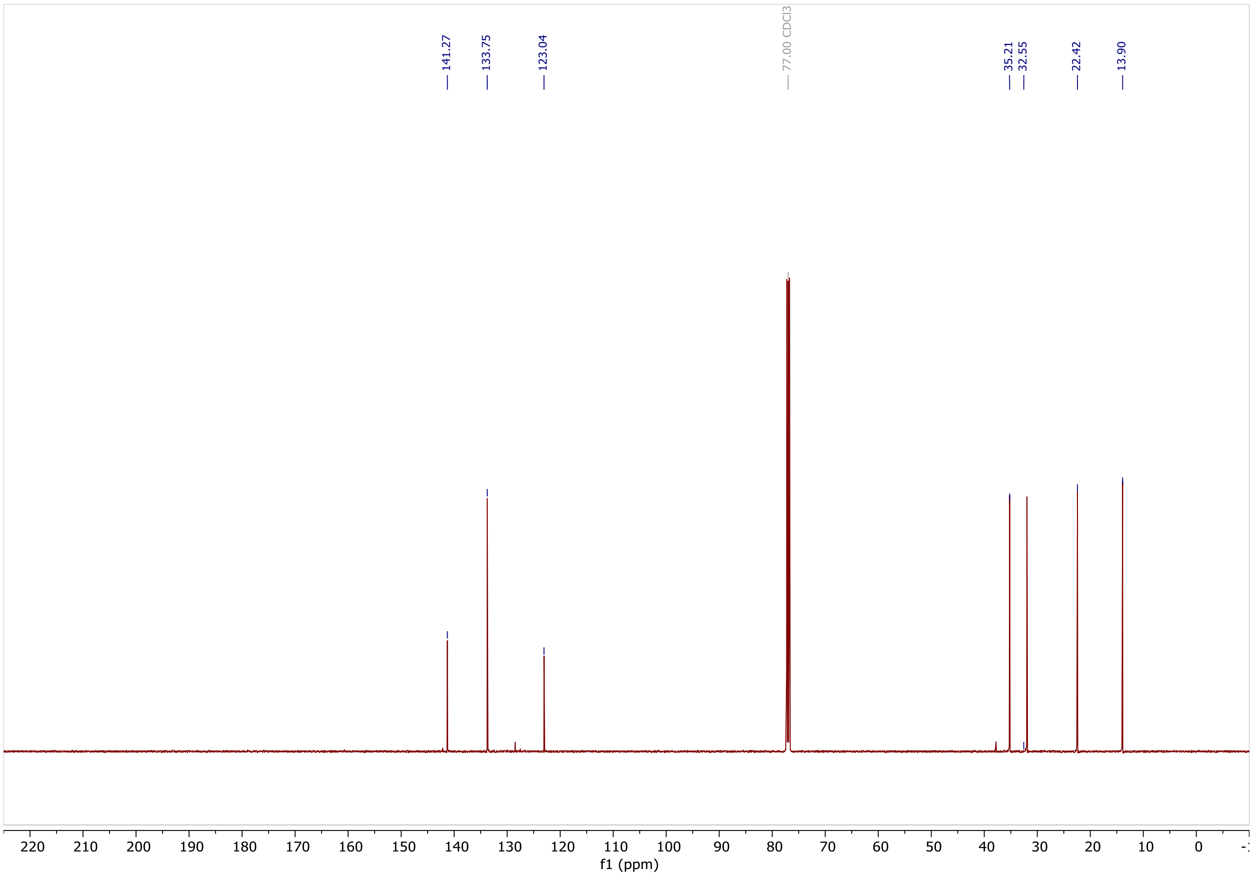

Supplementary Figure 29. ^{13}C-NMR spectrum (126 MHz, $CDCl_3$) for **SI-3**.

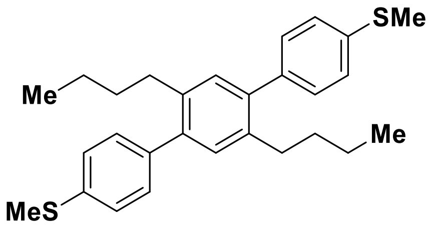

R4

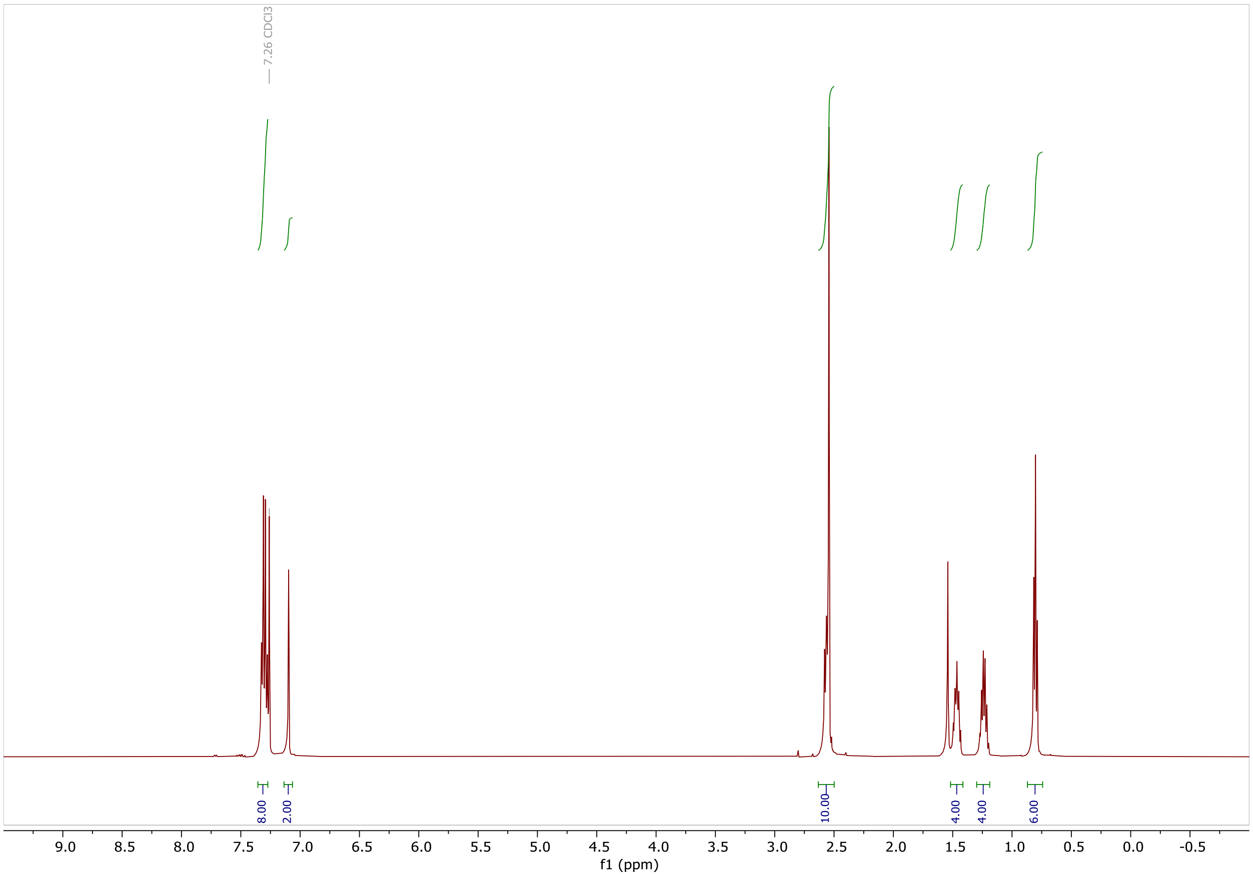

Supplementary Figure 30. ^{1}H-NMR spectrum (500 MHz, $CDCl_3$) for **R4**.

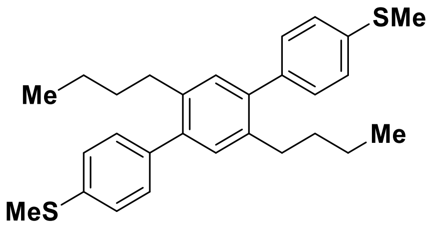

R4

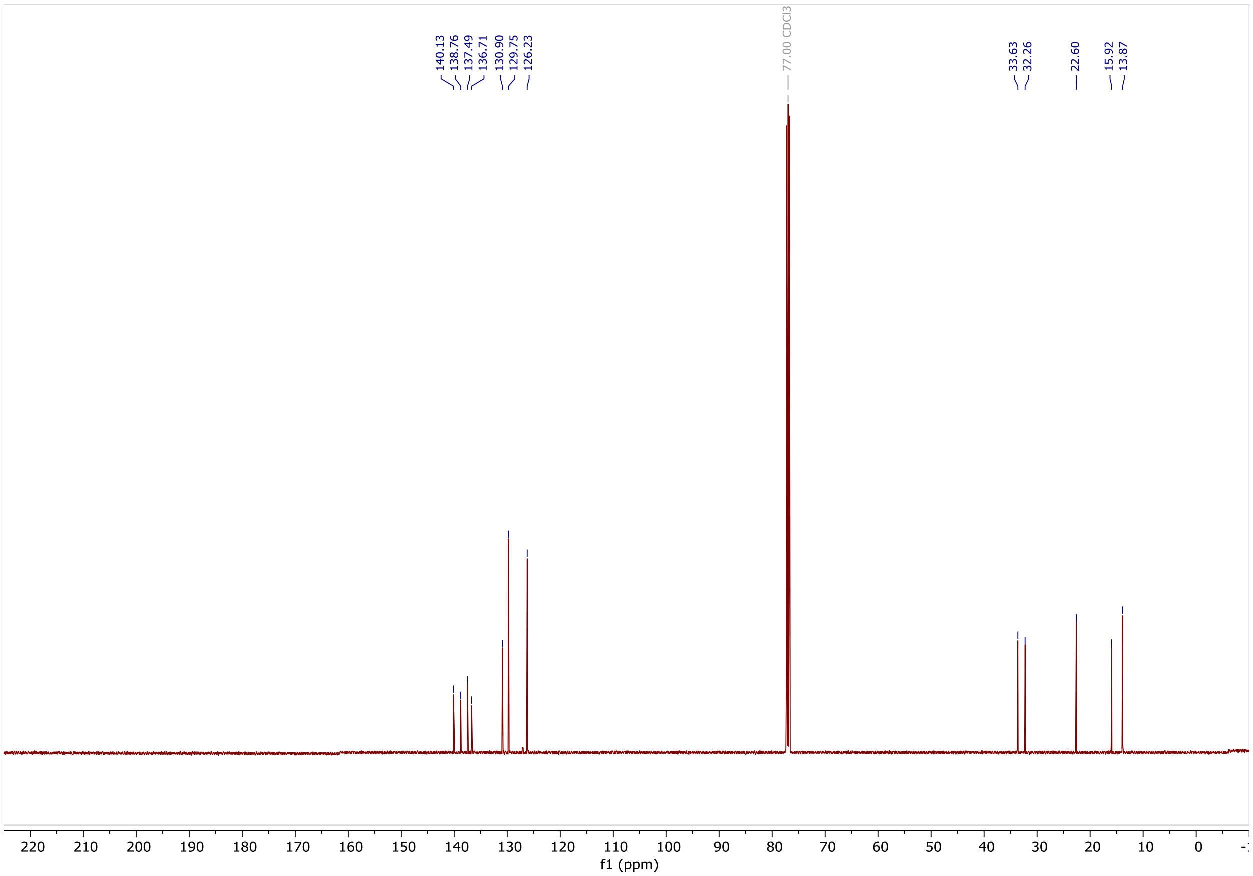

Supplementary Figure 31. ^{13}C-NMR spectrum (126 MHz, $CDCl_3$) for **R4**.

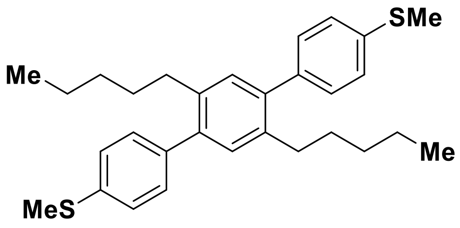

R5

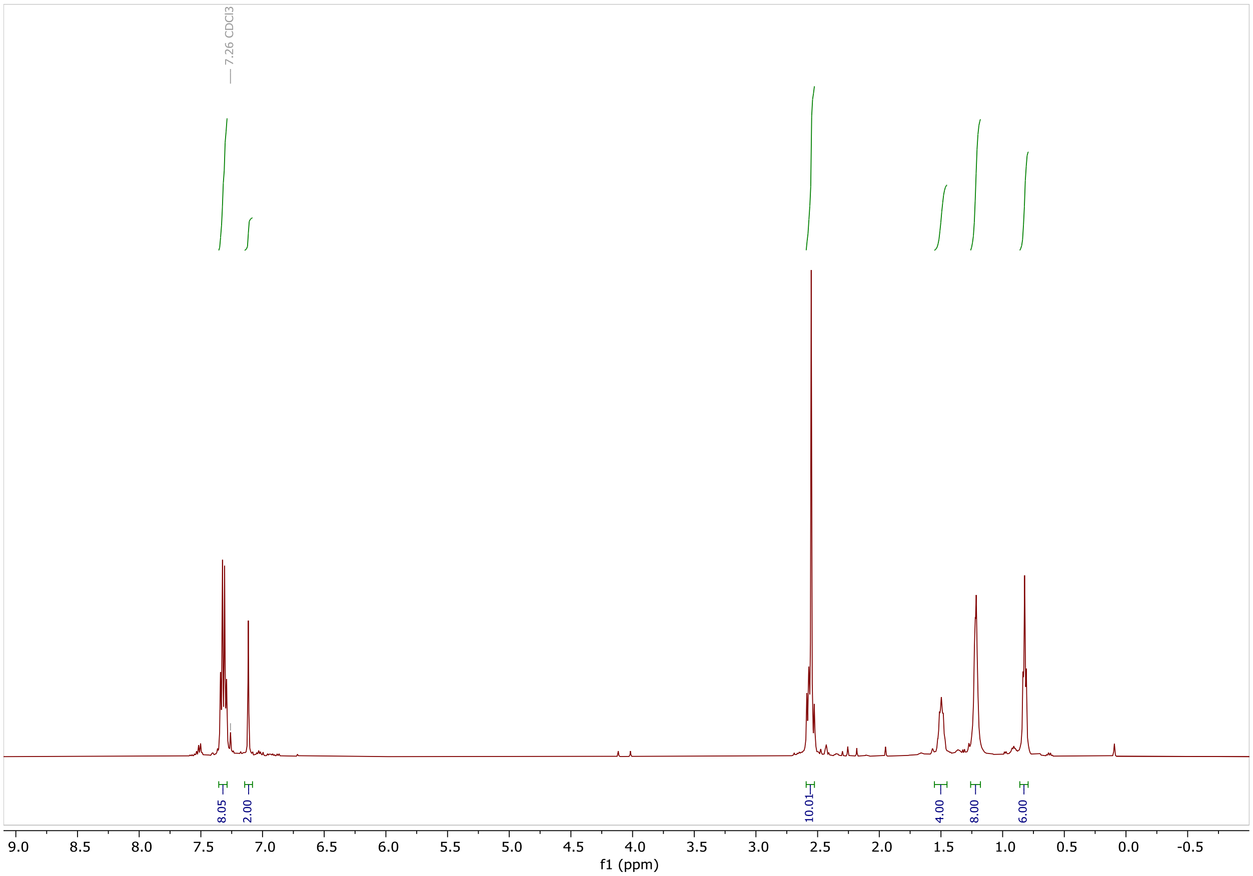

Supplementary Figure 32. ^{1}H-NMR spectrum (500 MHz, $CDCl_3$) for **R5**.

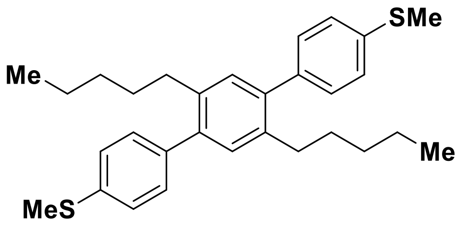

R5

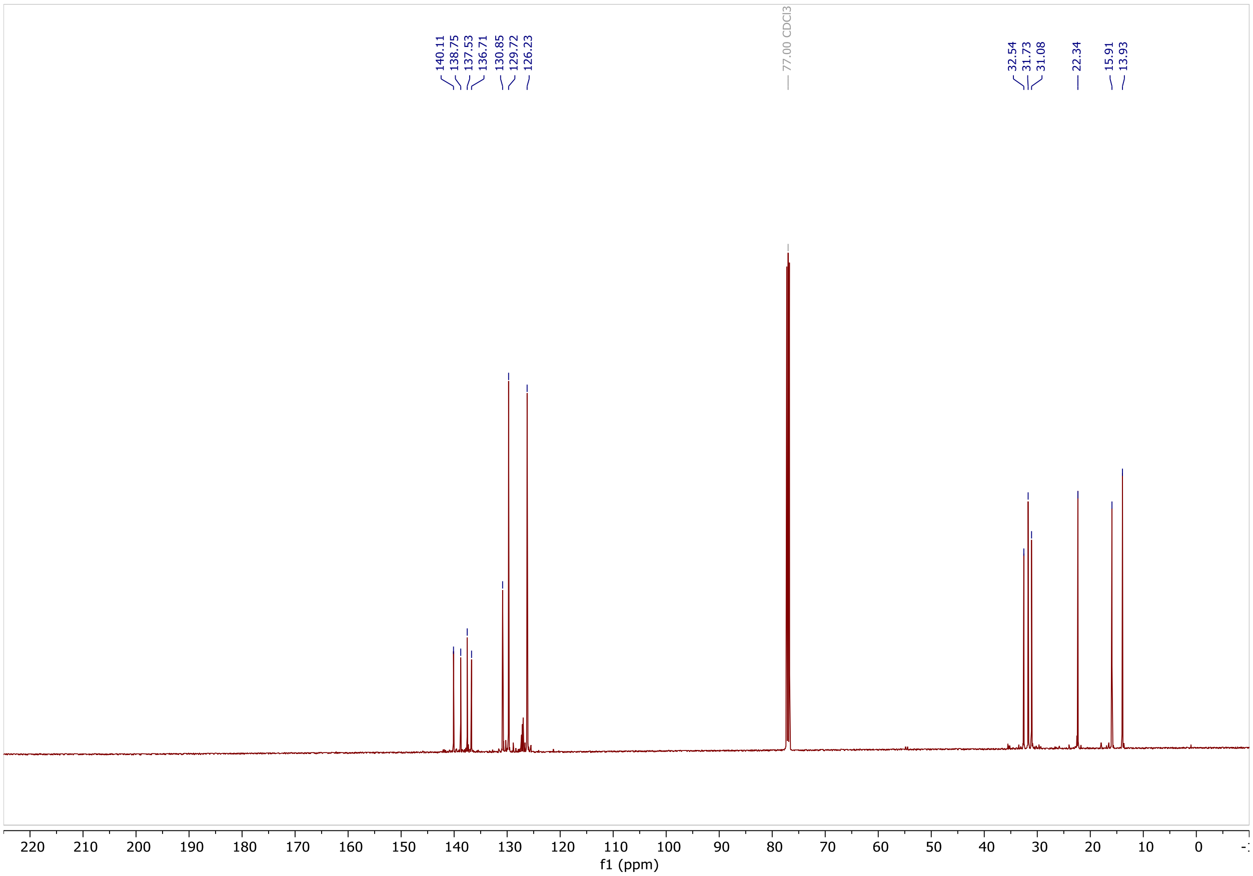

Supplementary Figure 33. ^{13}C-NMR spectrum (126 MHz, $CDCl_3$) for **R5**.

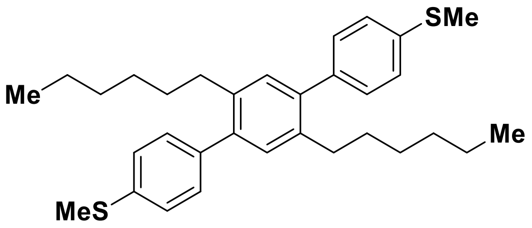

R6

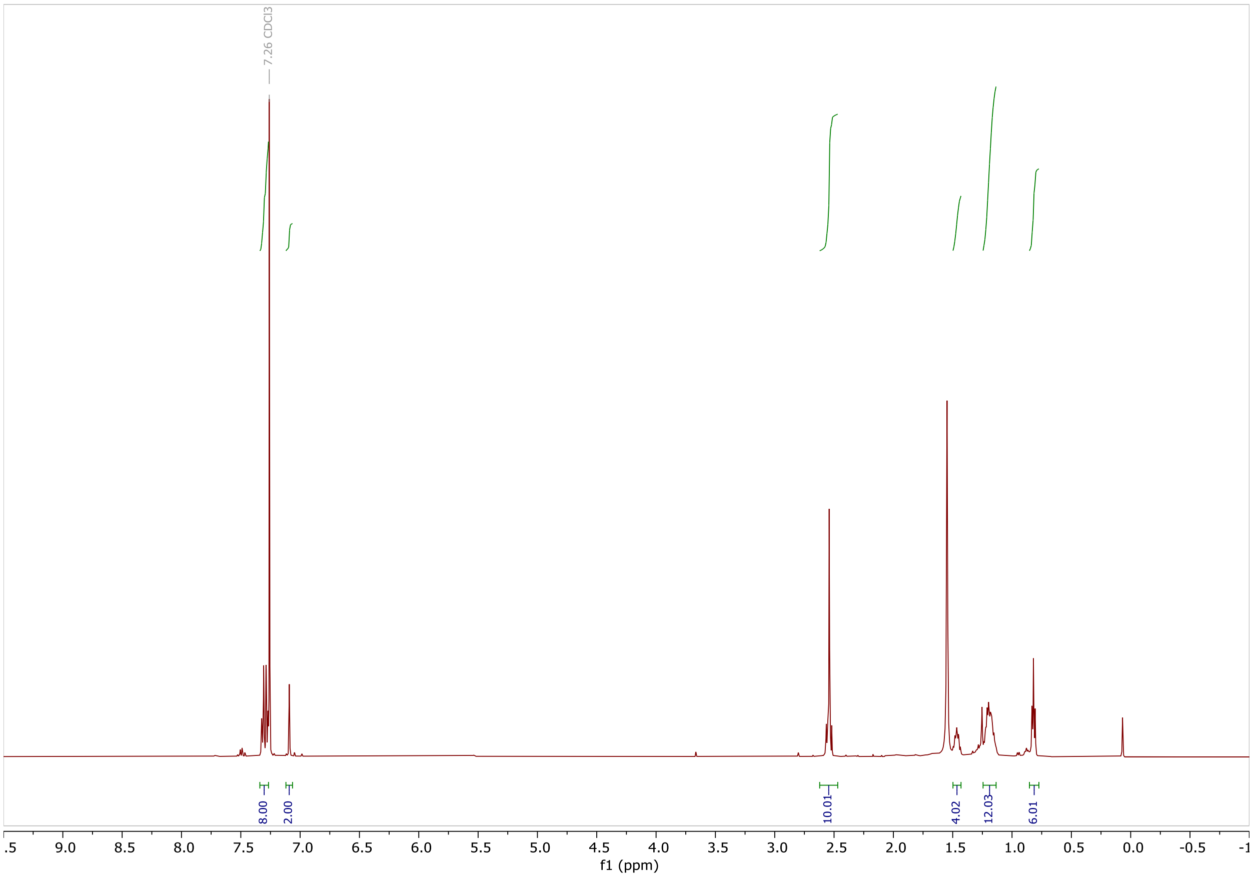

Supplementary Figure 34. ^{1}H-NMR spectrum (500 MHz, $CDCl_3$) for **R6**.

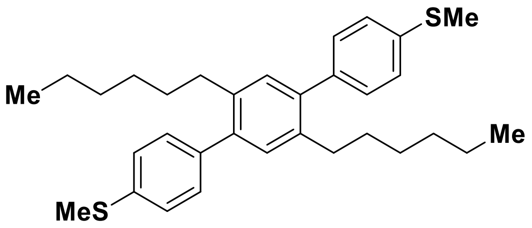

R6

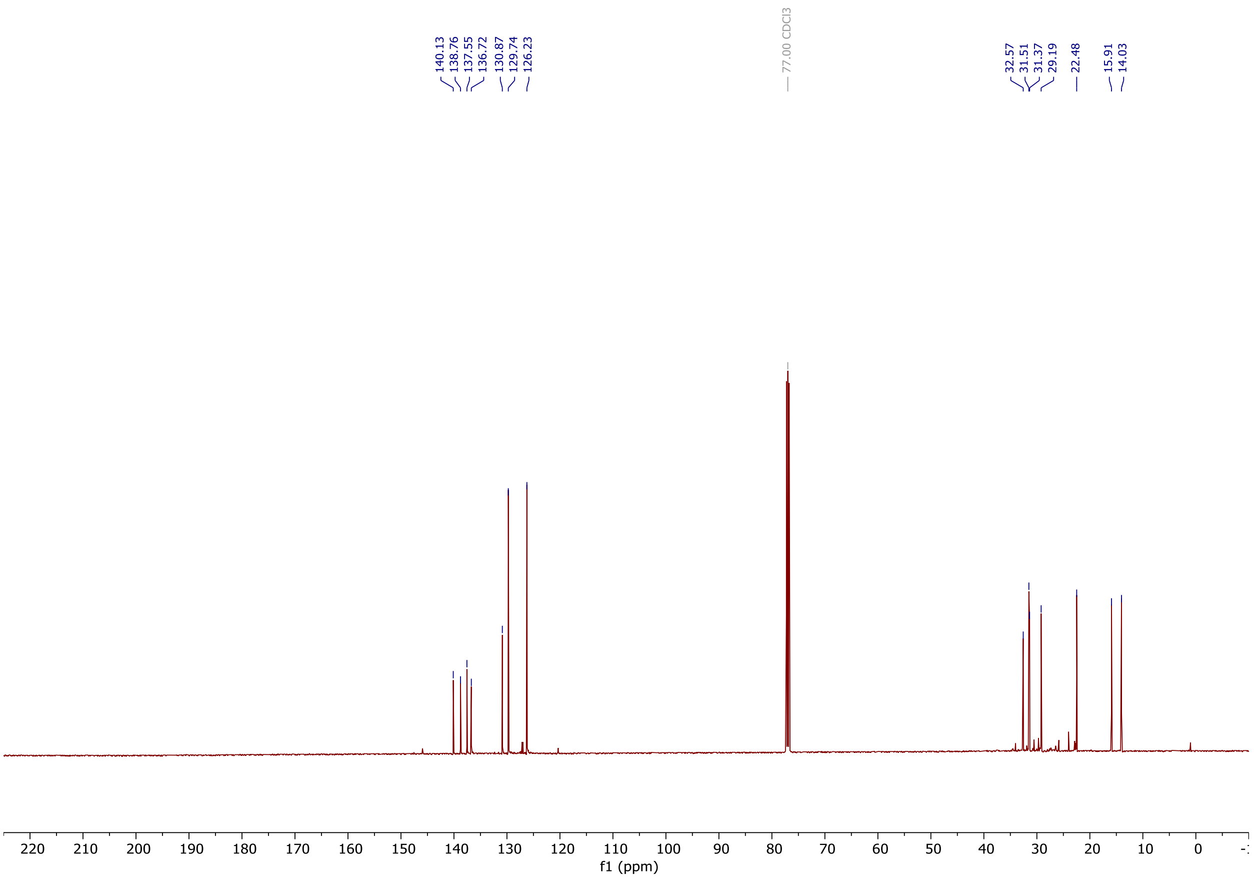

Supplementary Figure 35. ^{13}C-NMR spectrum (126 MHz, $CDCl_3$) for **R6**.

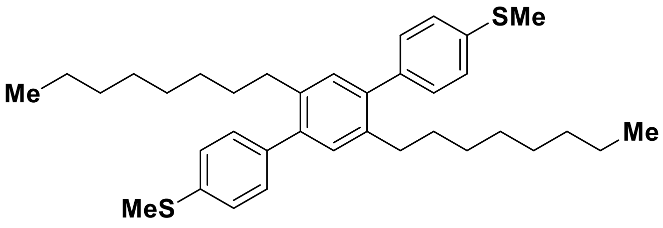

R8

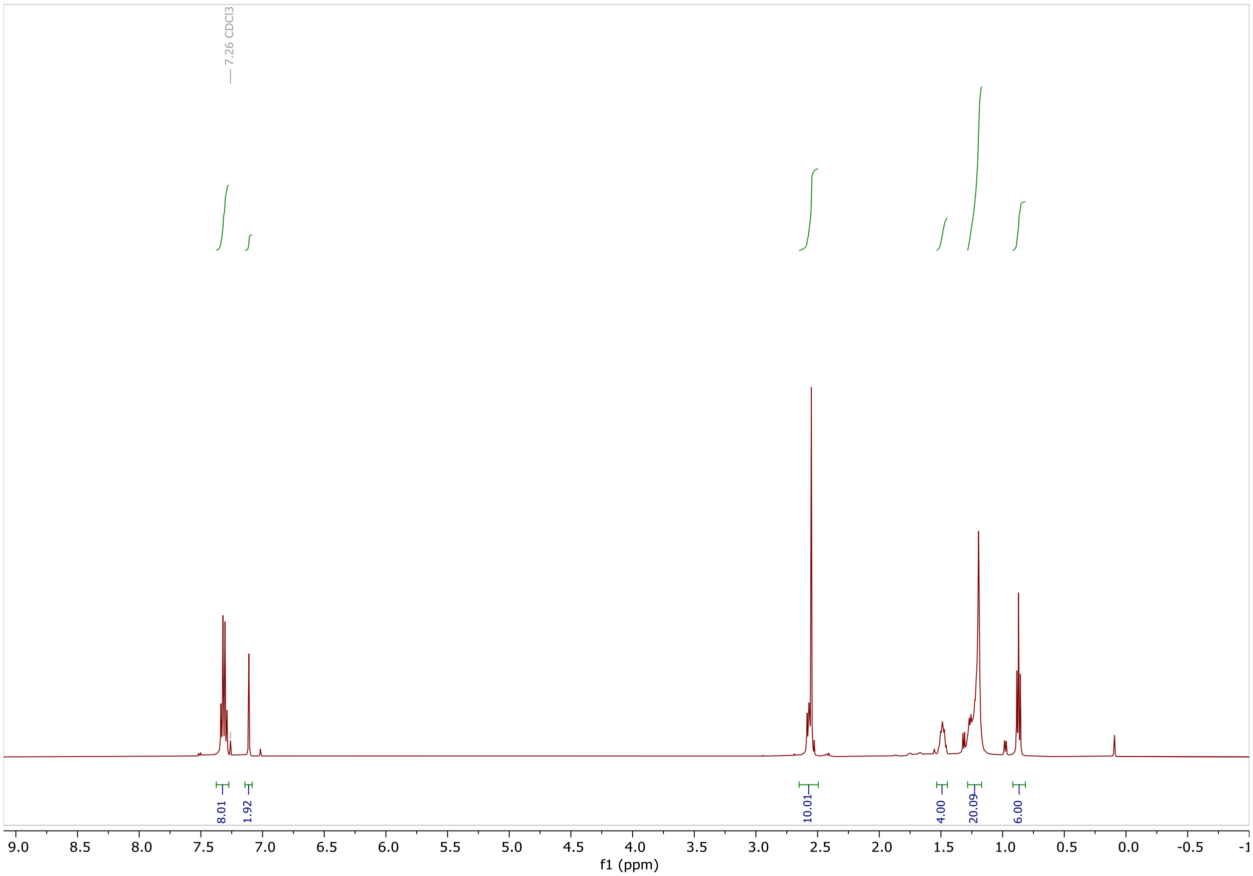

Supplementary Figure 36. ^{1}H-NMR spectrum (500 MHz, $CDCl_3$) for **R8**.

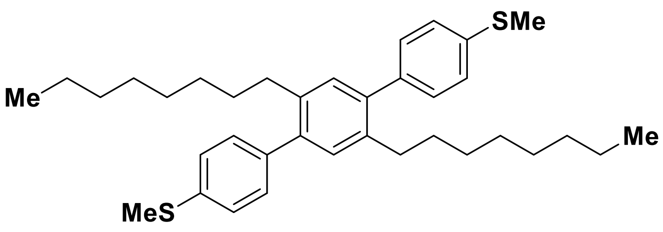

R8

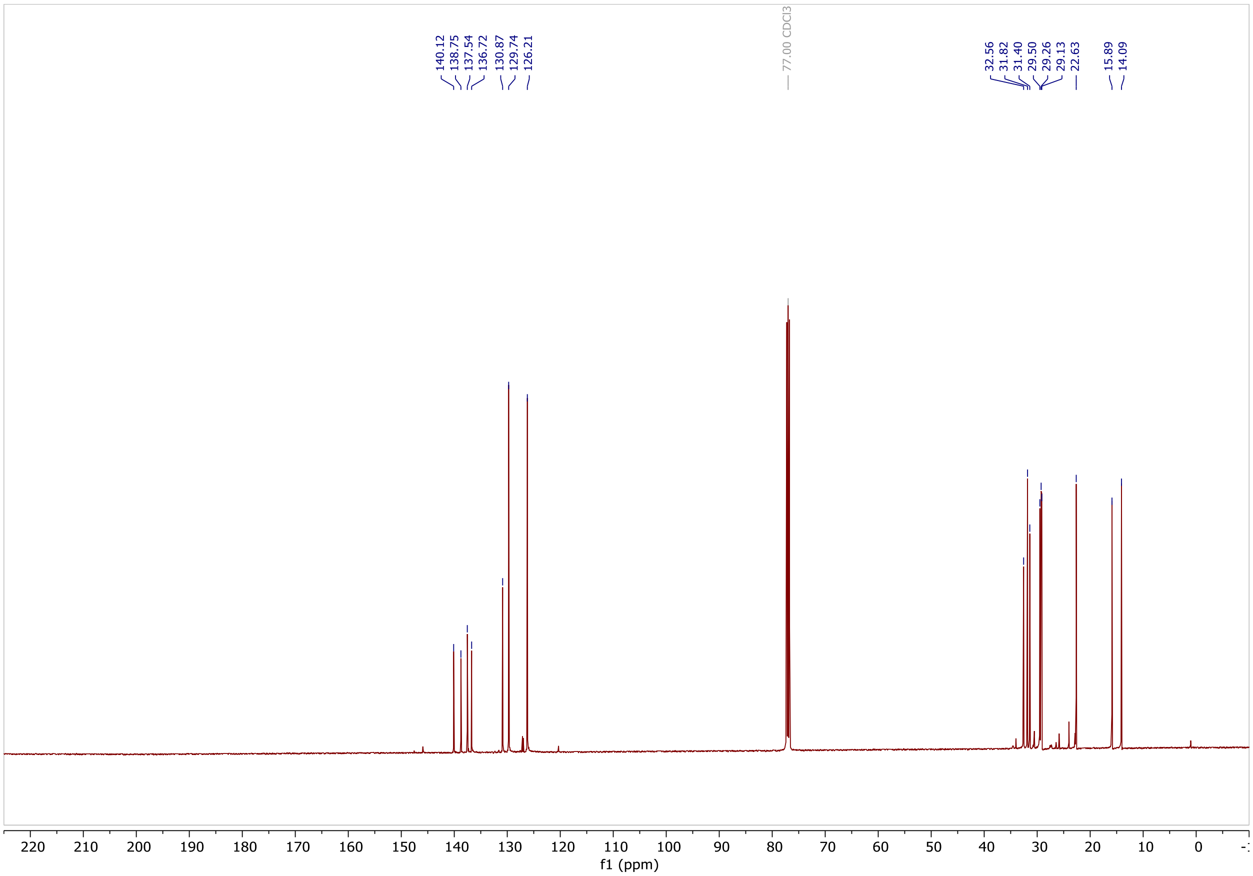

Supplementary Figure 37. ^{13}C-NMR spectrum (126 MHz, $CDCl_3$) for **R8**.

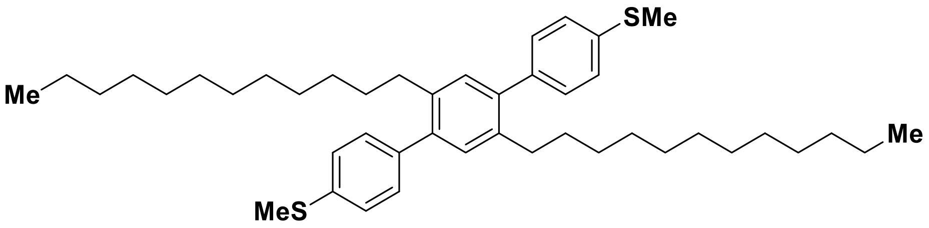

R12

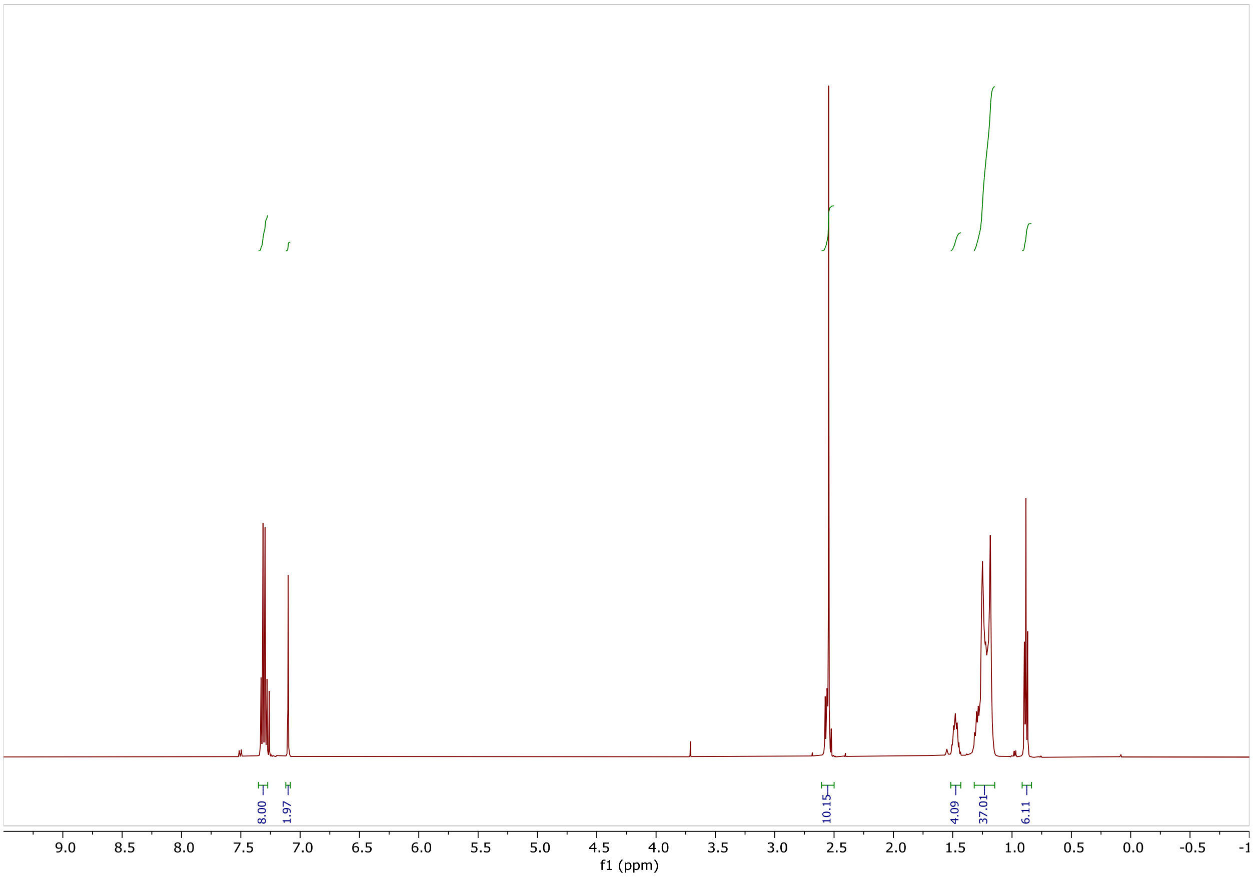

Supplementary Figure 38. ^{1}H-NMR spectrum (500 MHz, $CDCl_3$) for **R12**.

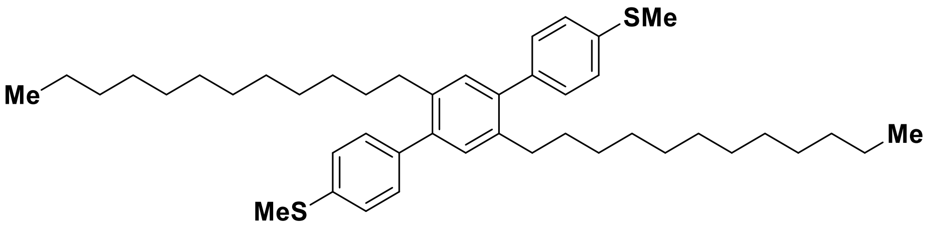

R12

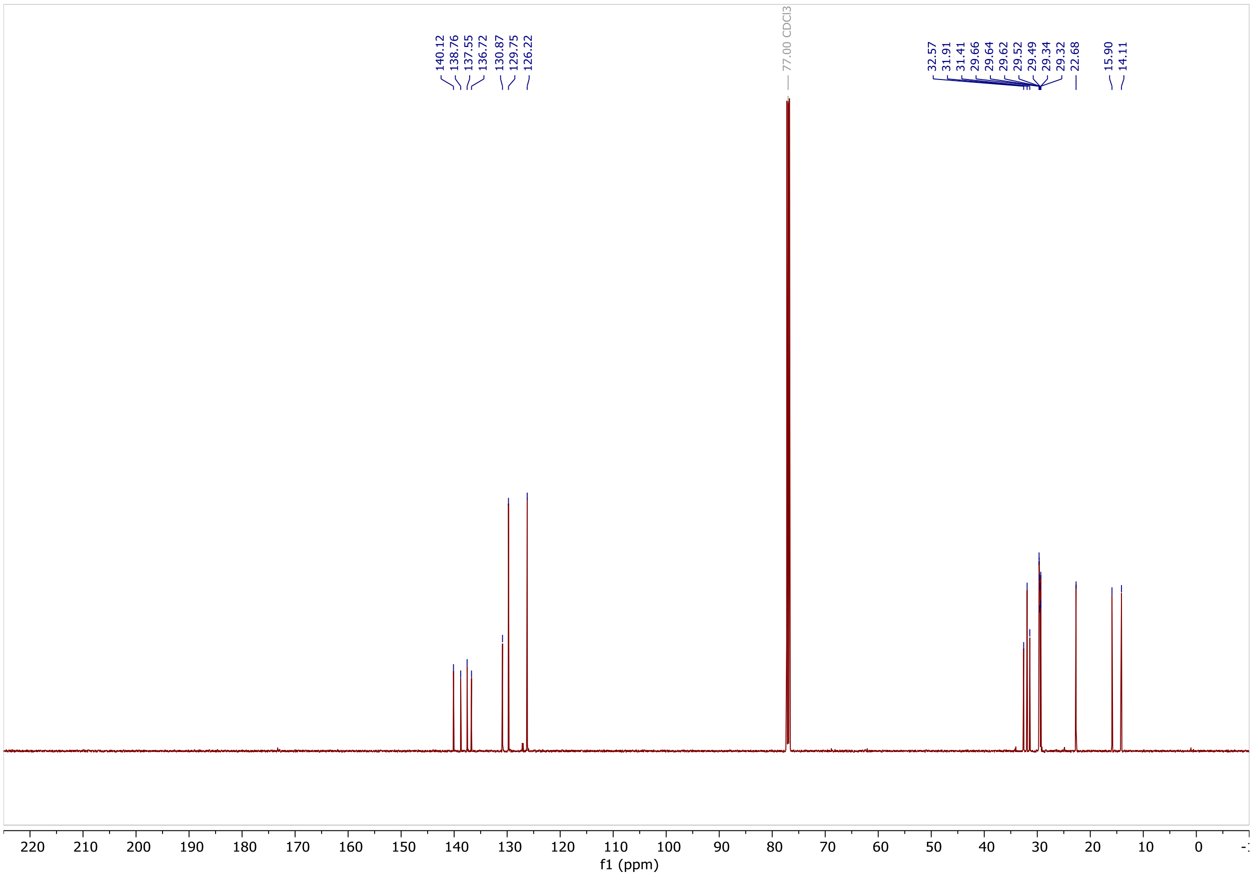

Supplementary Figure 39. ^{13}C-NMR spectrum (126 MHz, $CDCl_3$) for **R12**.

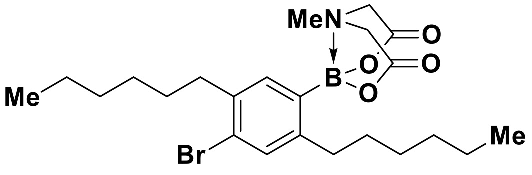

SI-6

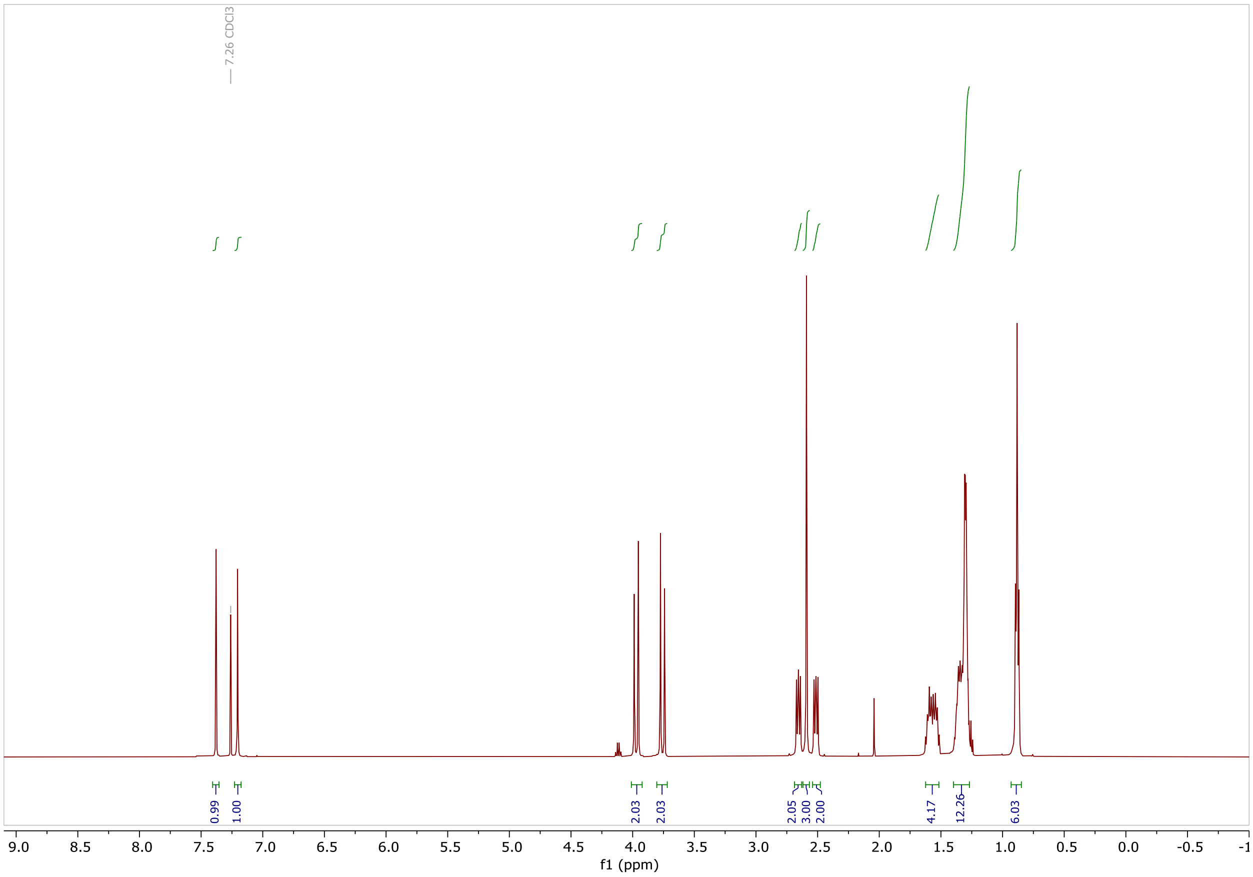

Supplementary Figure 40. ^{1}H-NMR spectrum (500 MHz, $CDCl_3$) for **SI-6**.

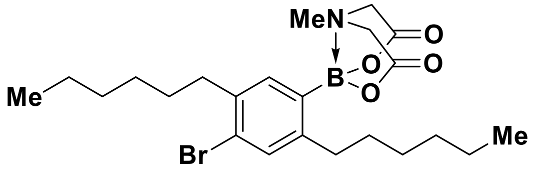

SI-6

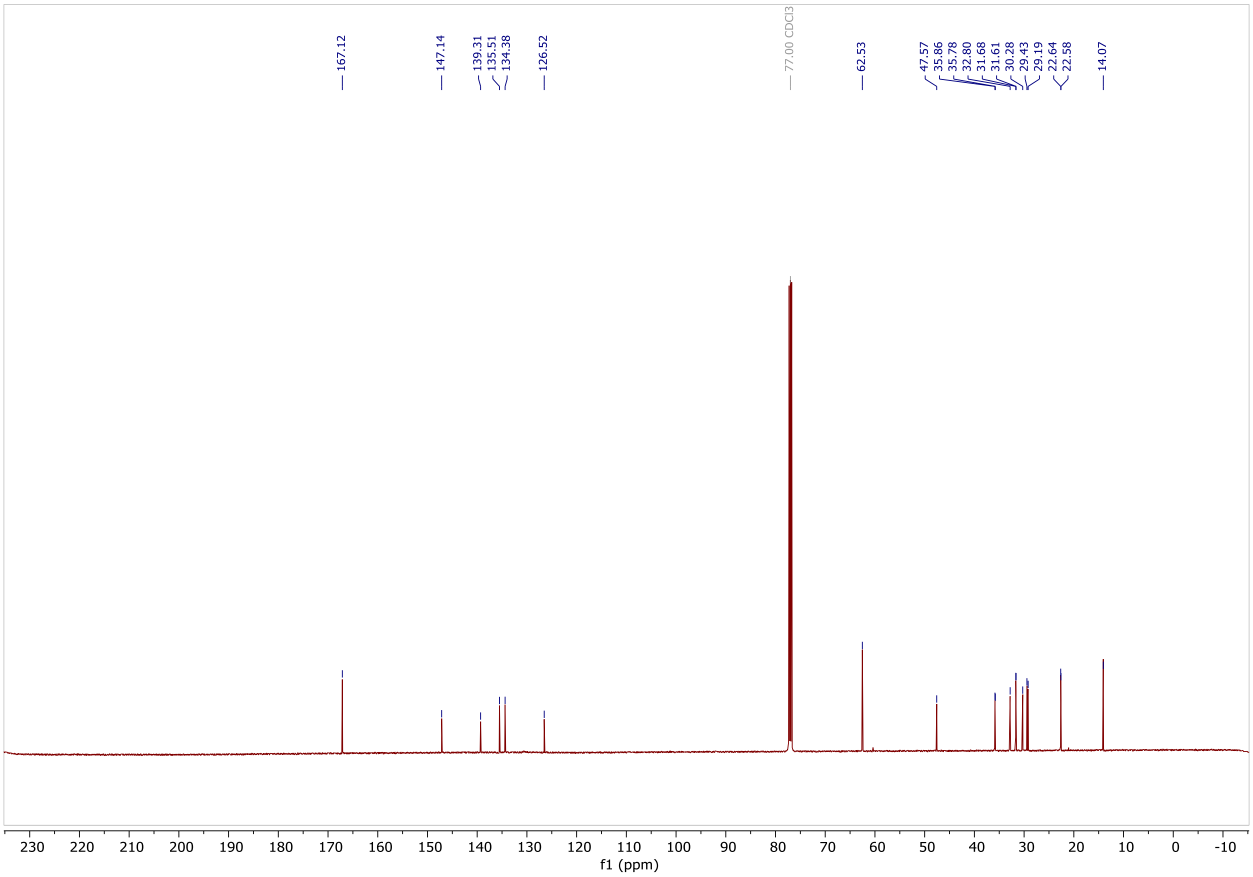

Supplementary Figure 41. ^{13}C-NMR spectrum (126 MHz, $CDCl_3$) for **SI-6**.

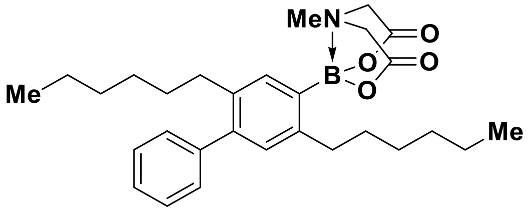

SI-7

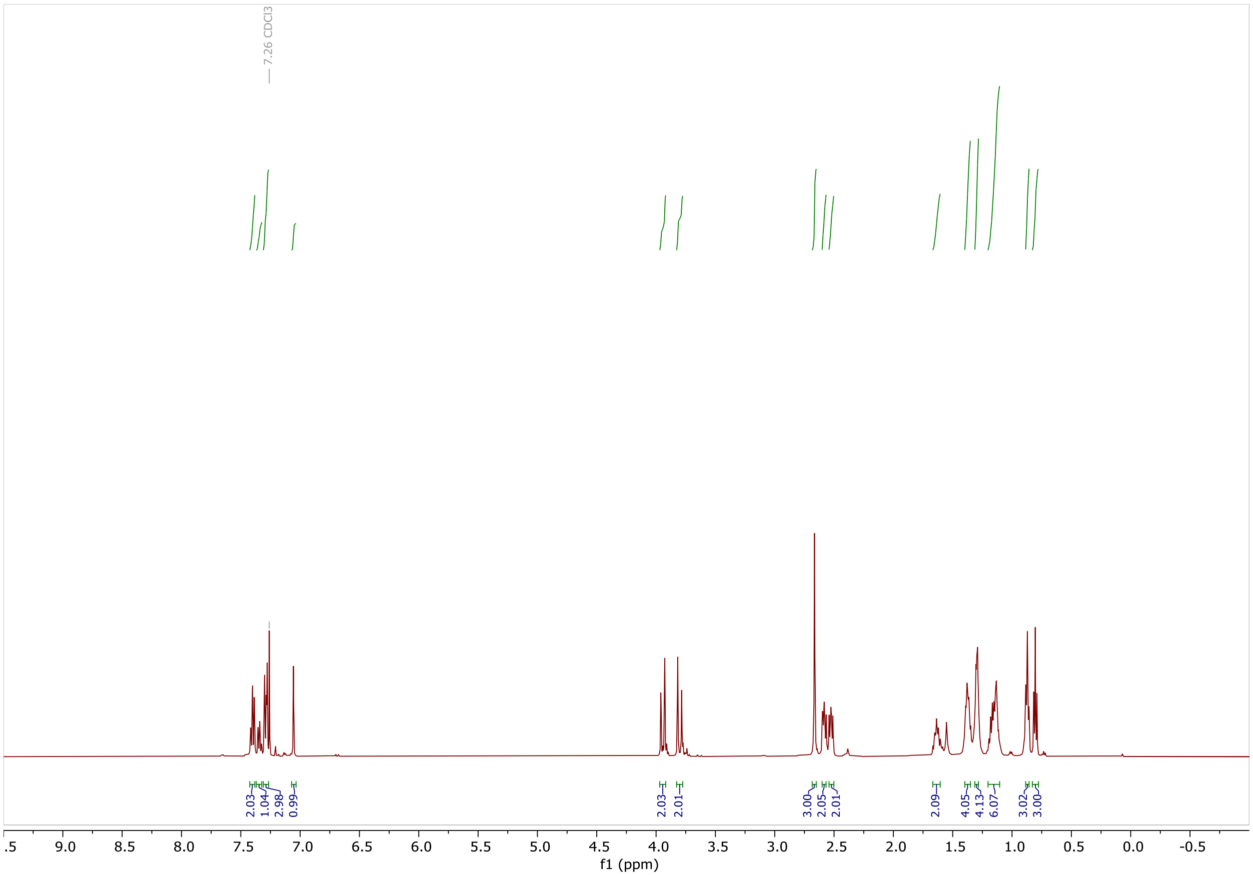

Supplementary Figure 42. ^{1}H-NMR spectrum (500 MHz, $CDCl_3$) for **SI-7**.

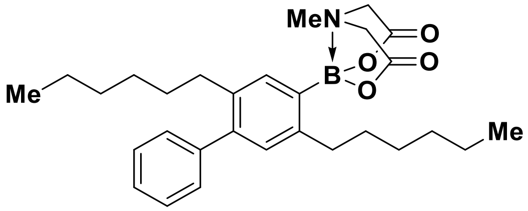

SI-7

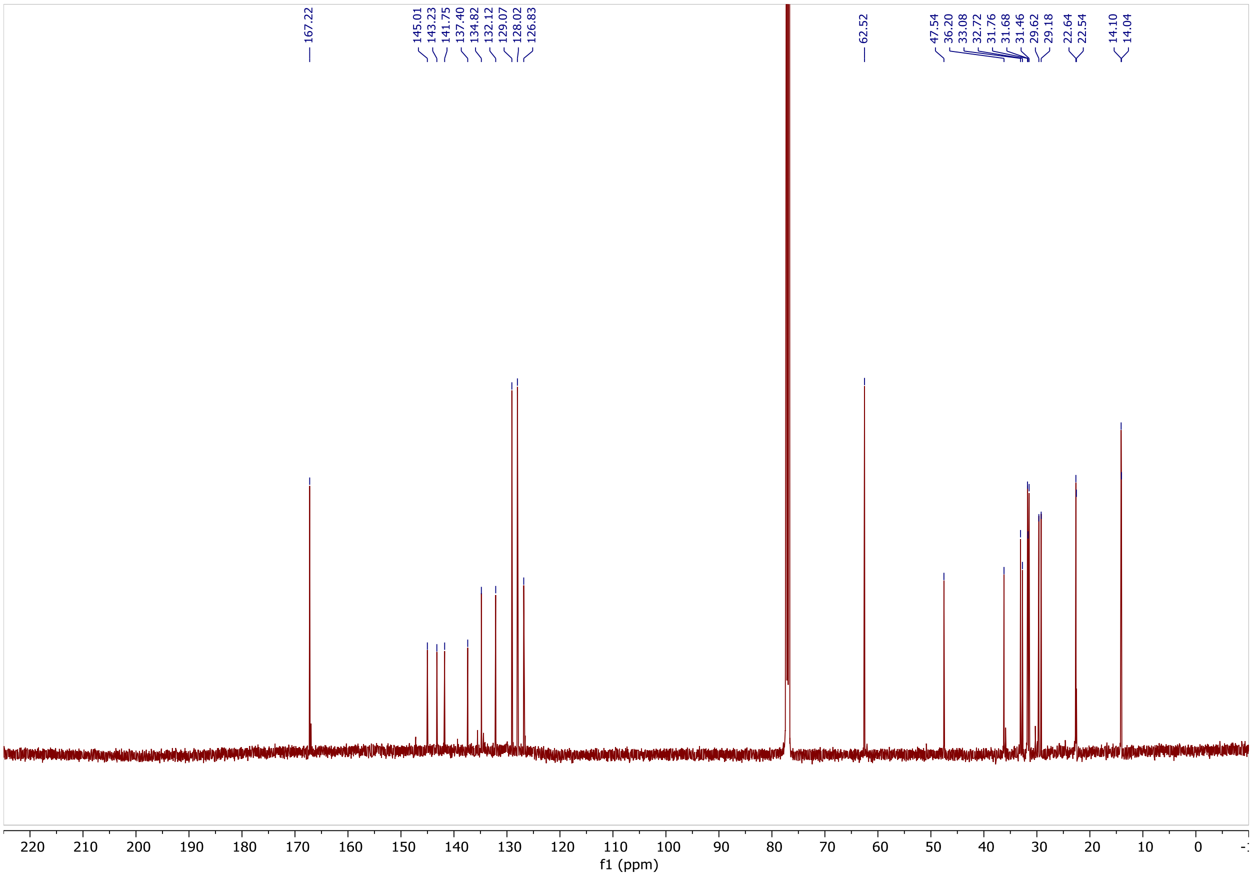

Supplementary Figure 43. ^{13}C-NMR spectrum (126 MHz, $CDCl_3$) for **SI-7**.

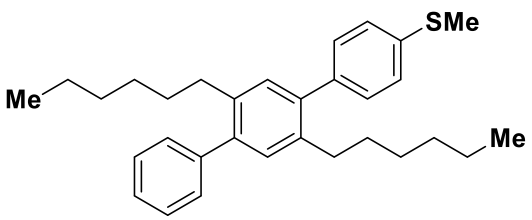

R6-H

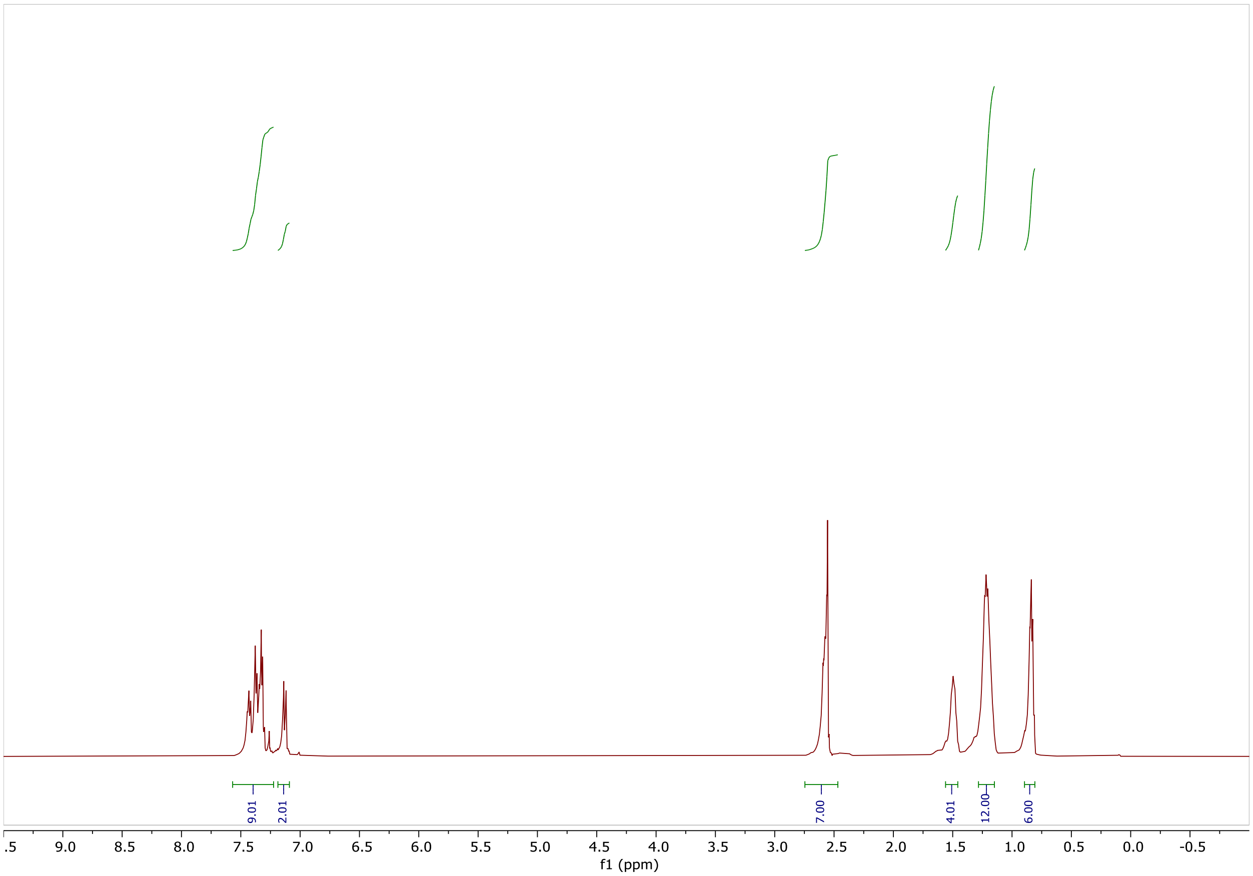

Supplementary Figure 44. ^{1}H-NMR spectrum (500 MHz, $CDCl_3$) for **R6-H**.

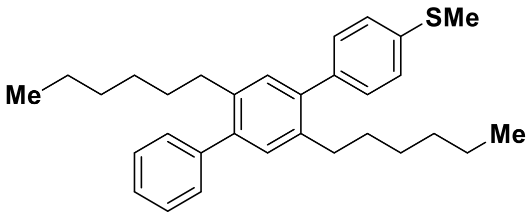

R6-H

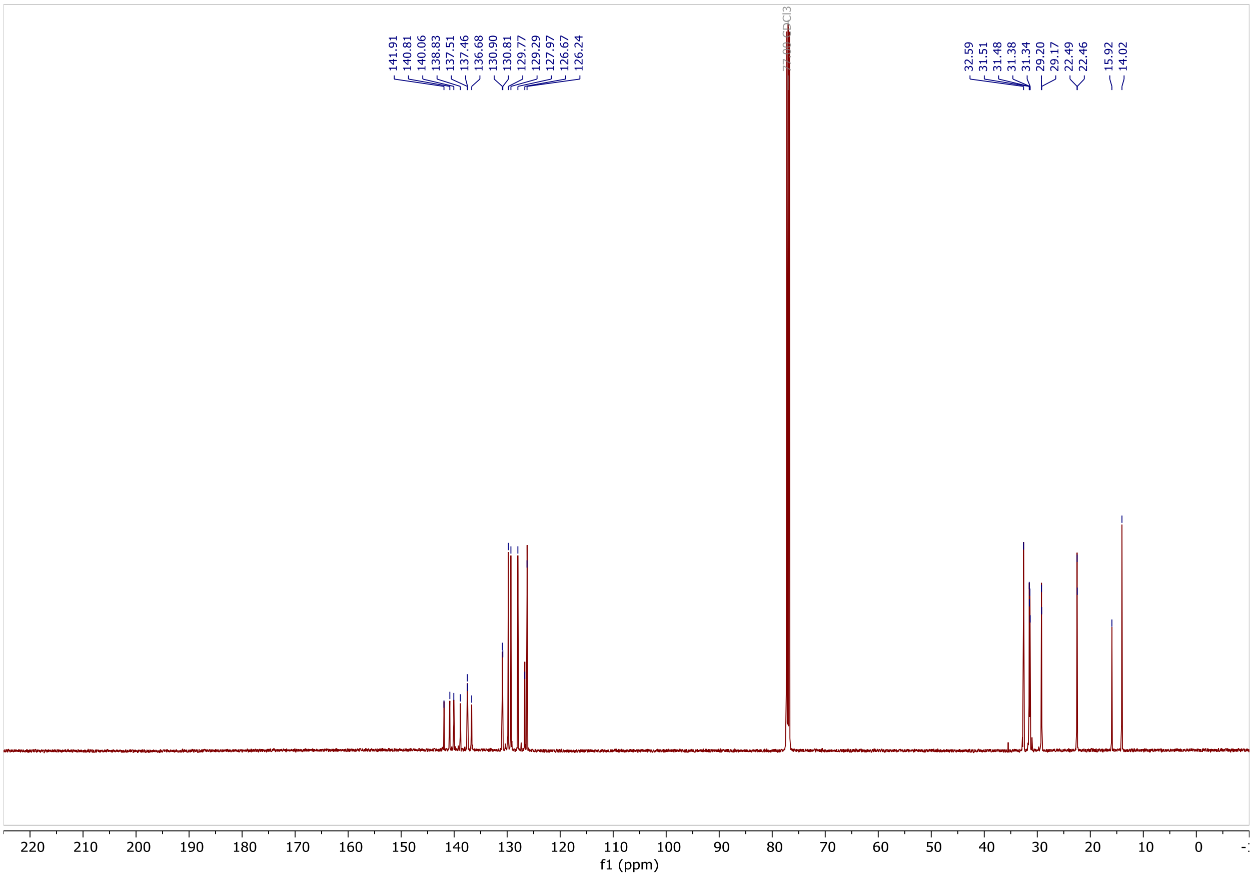

Supplementary Figure 45. ^{13}C-NMR spectrum (126 MHz, $CDCl_3$) for **R6-H**.

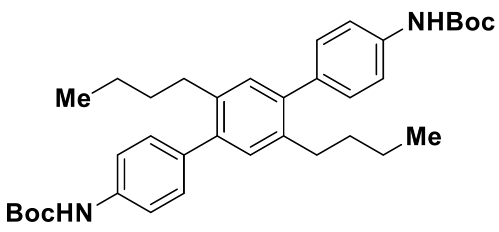

R4-NHBoc

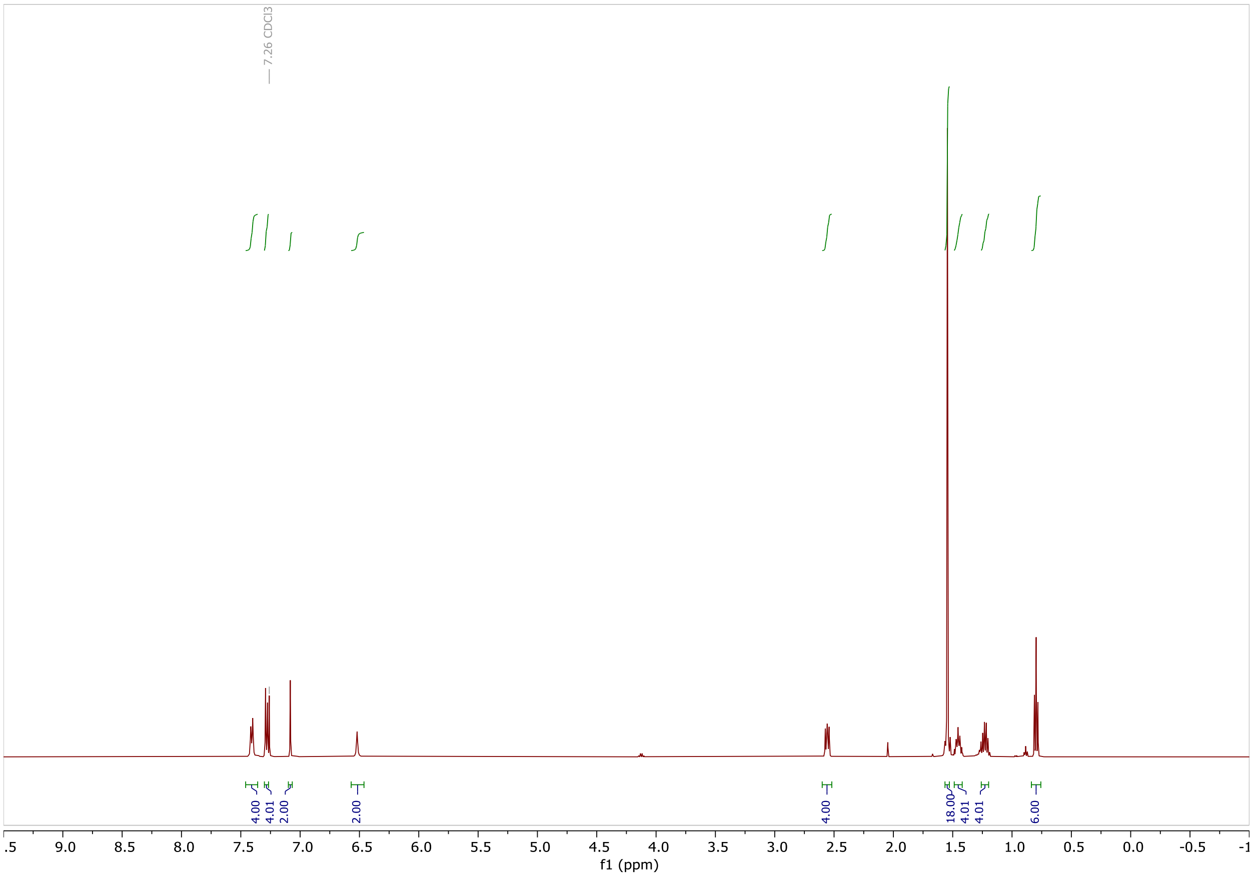

Supplementary Figure 46. ^{1}H-NMR spectrum (500 MHz, $CDCl_3$) for **R4-NHBoc**.

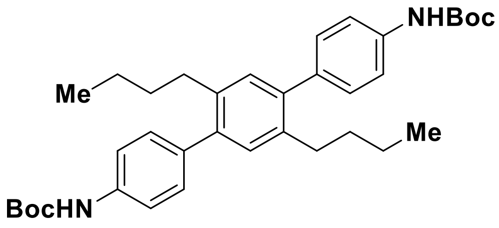

R4-NHBoc

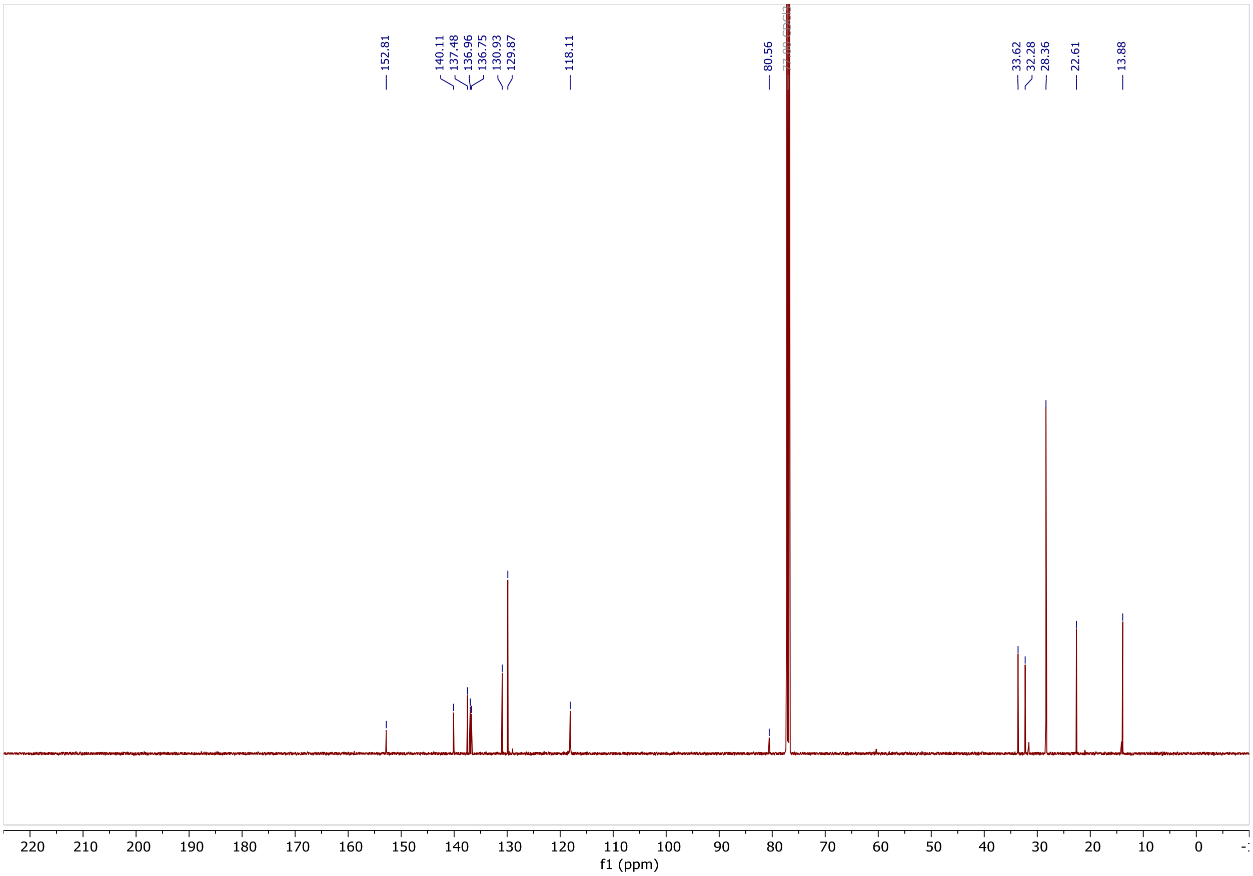

Supplementary Figure 47. ^{13}C-NMR spectrum (126 MHz, $CDCl_3$) for **R4-NHBoc**.

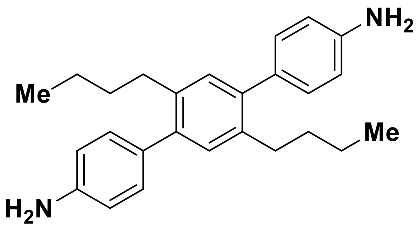

R4-N

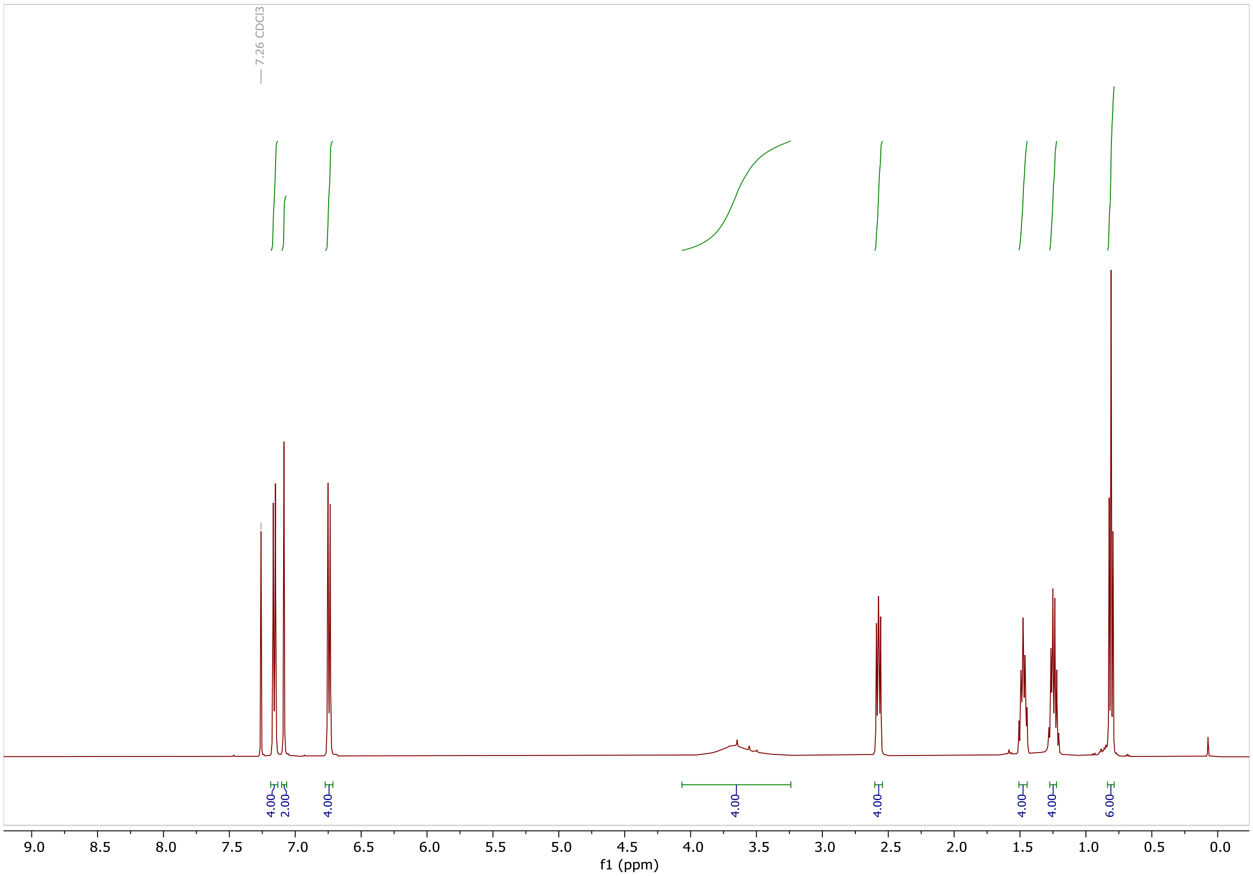

Supplementary Figure 48. ^{1}H-NMR spectrum (500 MHz, $CDCl_3$) for **R4-N**.

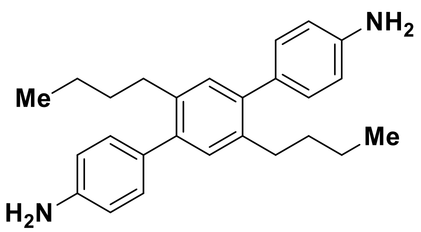

R4-N

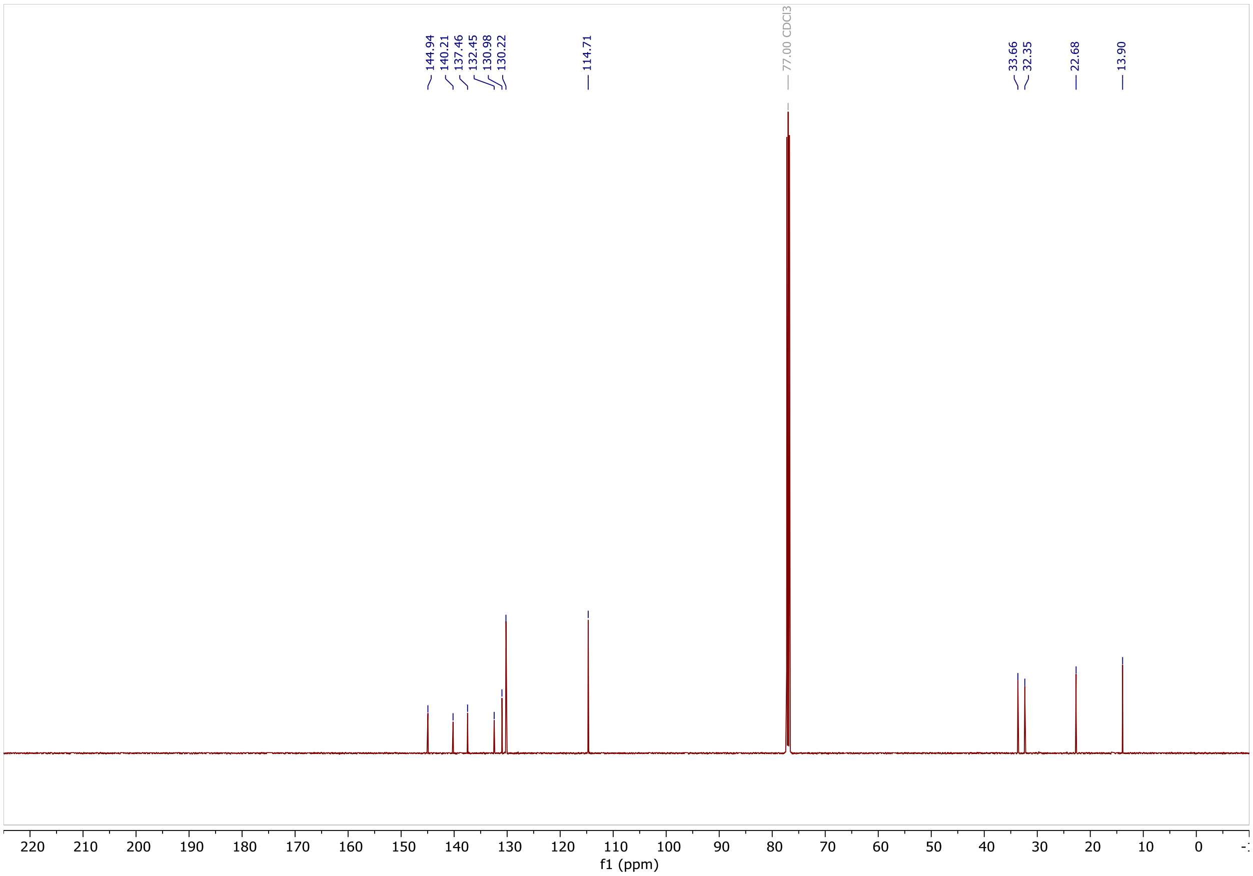

Supplementary Figure 49. ^{13}C-NMR spectrum (126 MHz, $CDCl_3$) for **R4-N**.

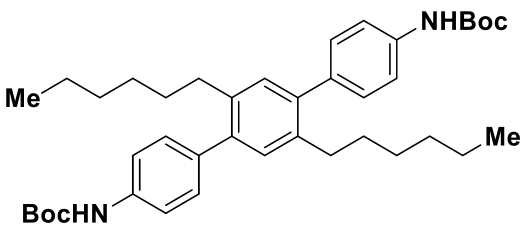

R6-NHBoc

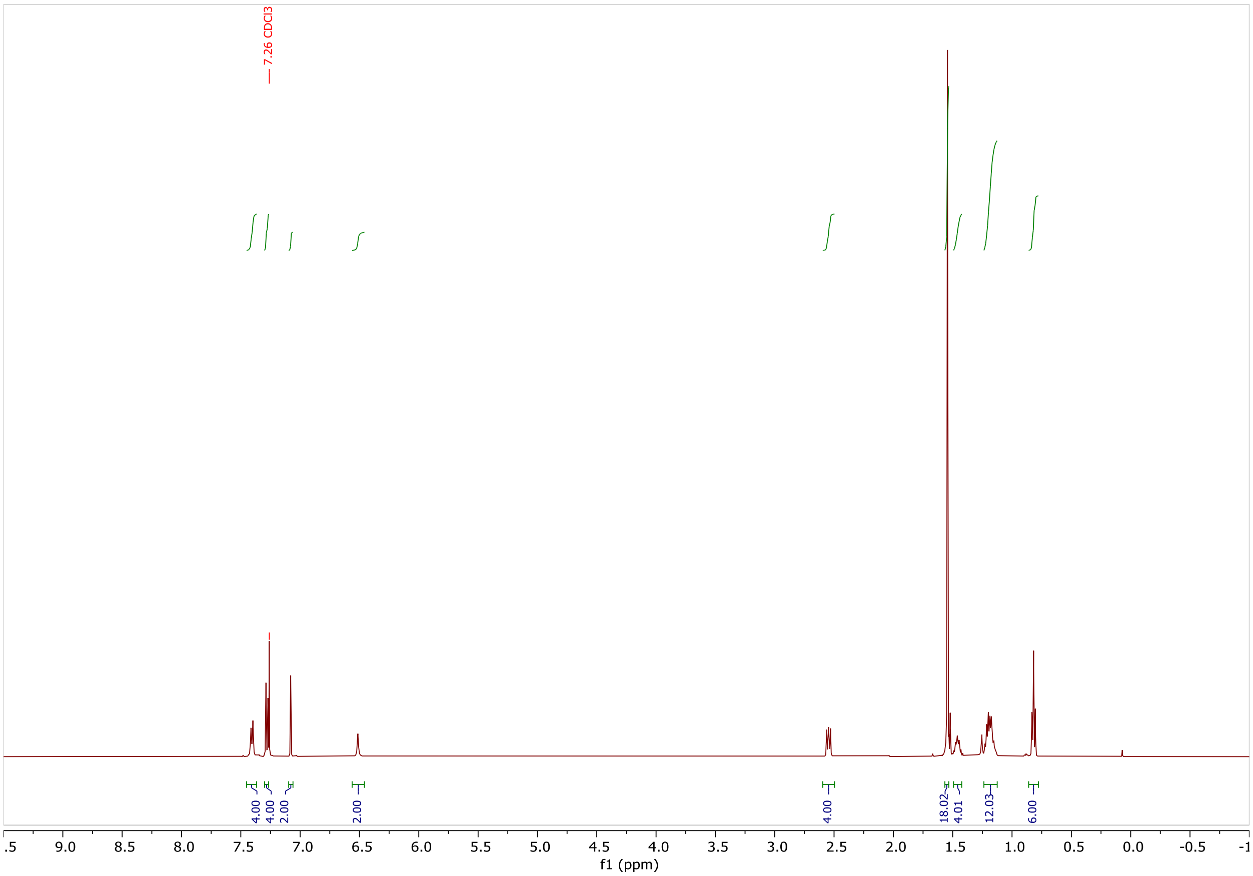

Supplementary Figure 50. ^{1}H-NMR spectrum (500 MHz, $CDCl_3$) for **R6-NHBoc.**

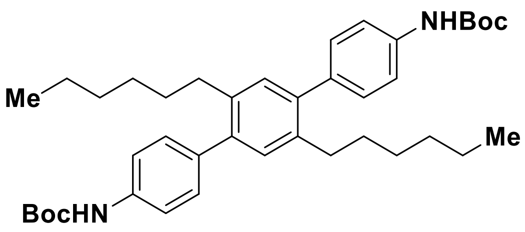

R6-NHBoc

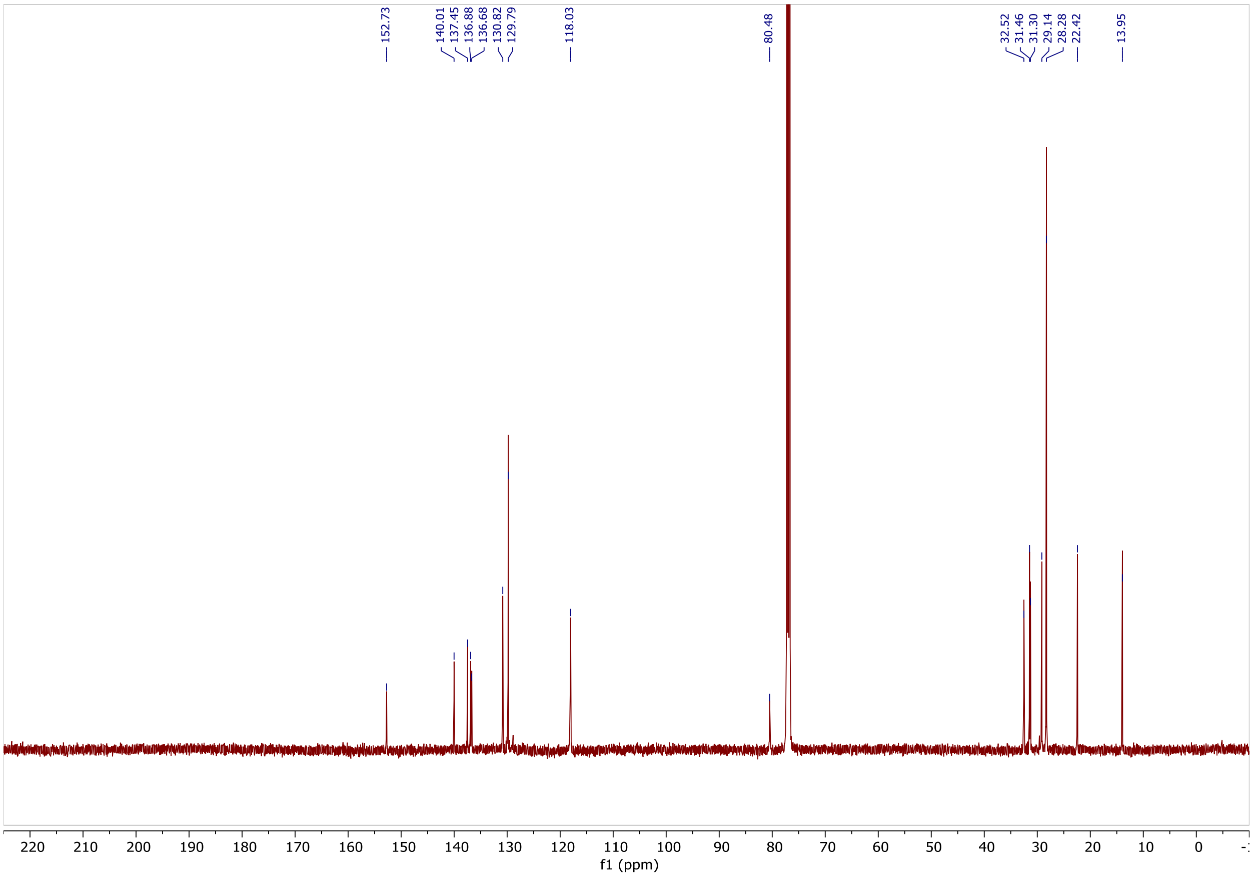

Supplementary Figure 51. ^{13}C-NMR spectrum (126 MHz, $CDCl_3$) for **R6-NHBoc**.

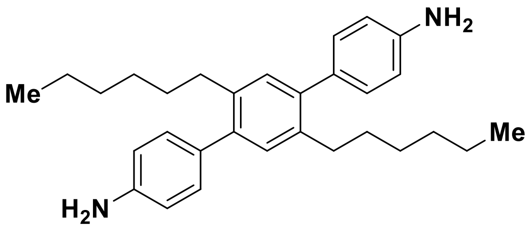

R6-N

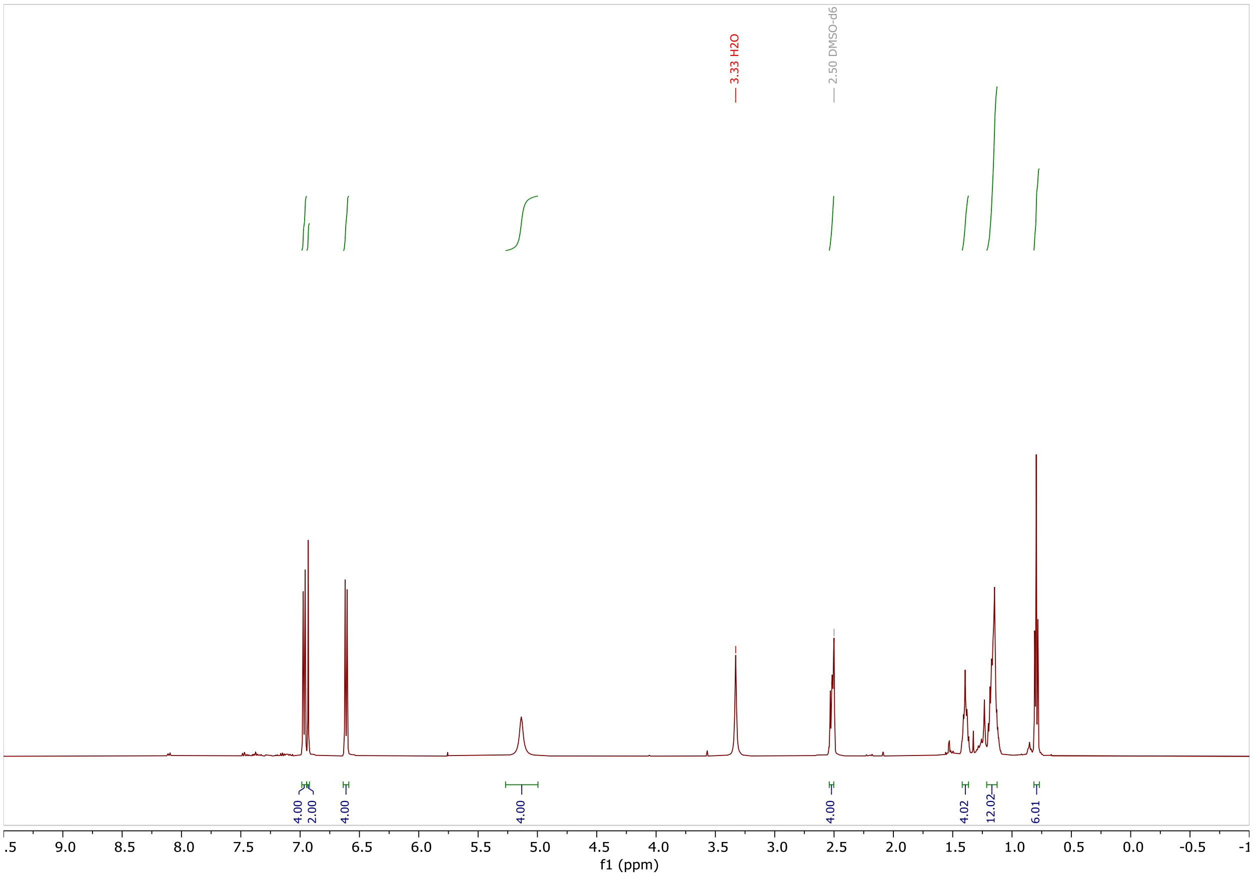

Supplementary Figure 52. ^{1}H-NMR spectrum (500 MHz, DMSO-*d*6) for **R6-N**.

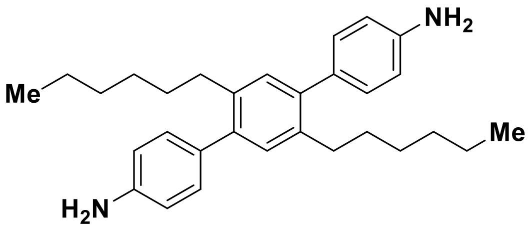

R6-N

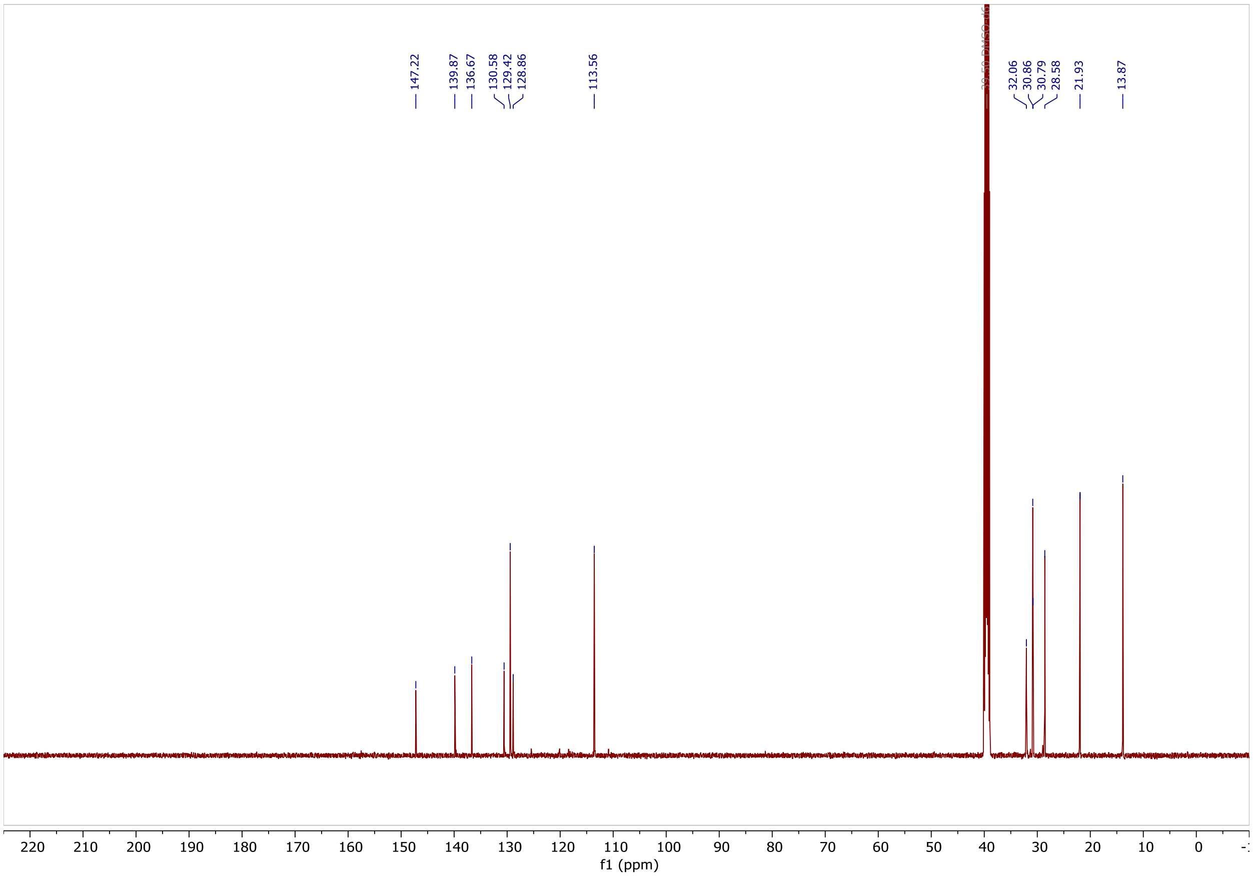

Supplementary Figure 53. ^{13}C-NMR spectrum (126 MHz, DMSO-*d*6) for **R6-N**.

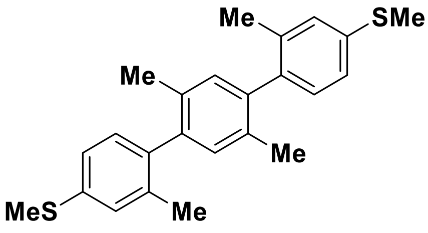

R1-twisted

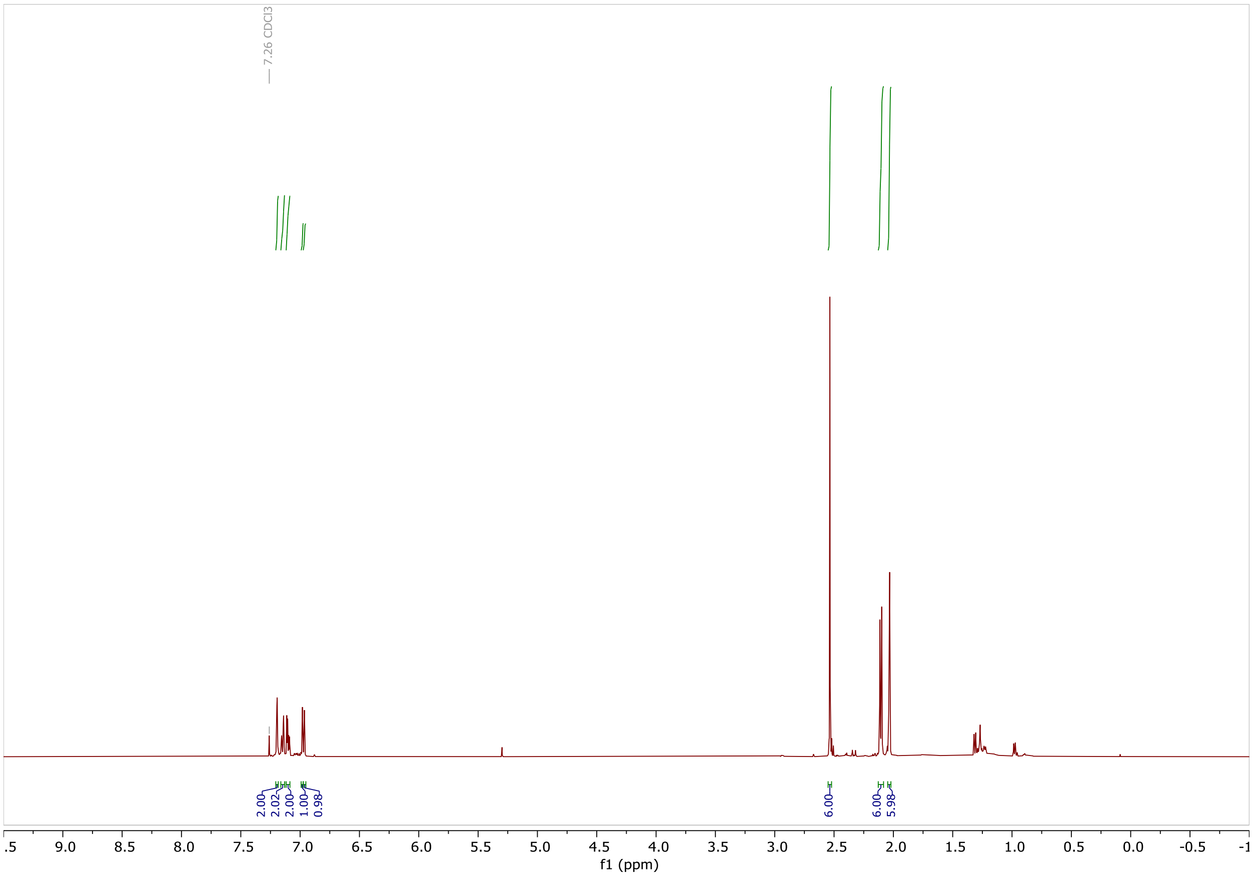

Supplementary Figure 54. ^{1}H-NMR spectrum (500 MHz, $CDCl_3$) for **R1-twisted**.

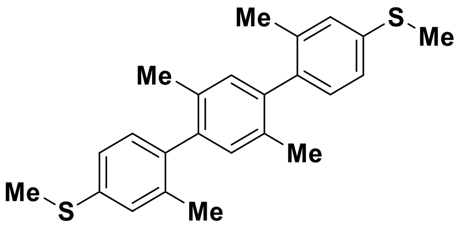

R1-twisted

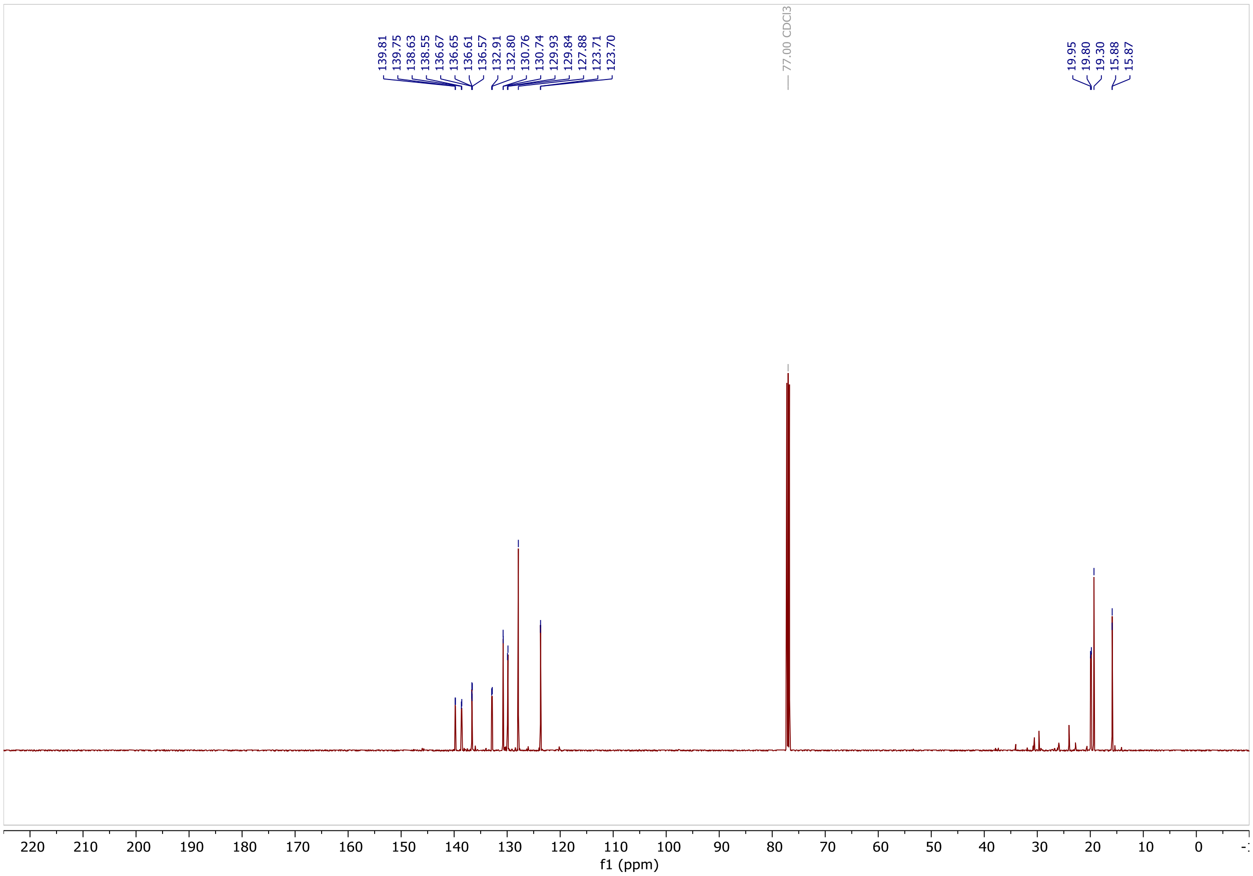

Supplementary Figure 55. ^{13}C-NMR spectrum (126 MHz, $CDCl_3$) for **R1-twisted**.

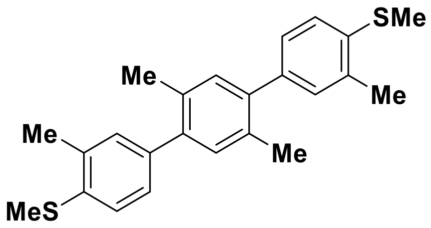

R1-*m*-Me

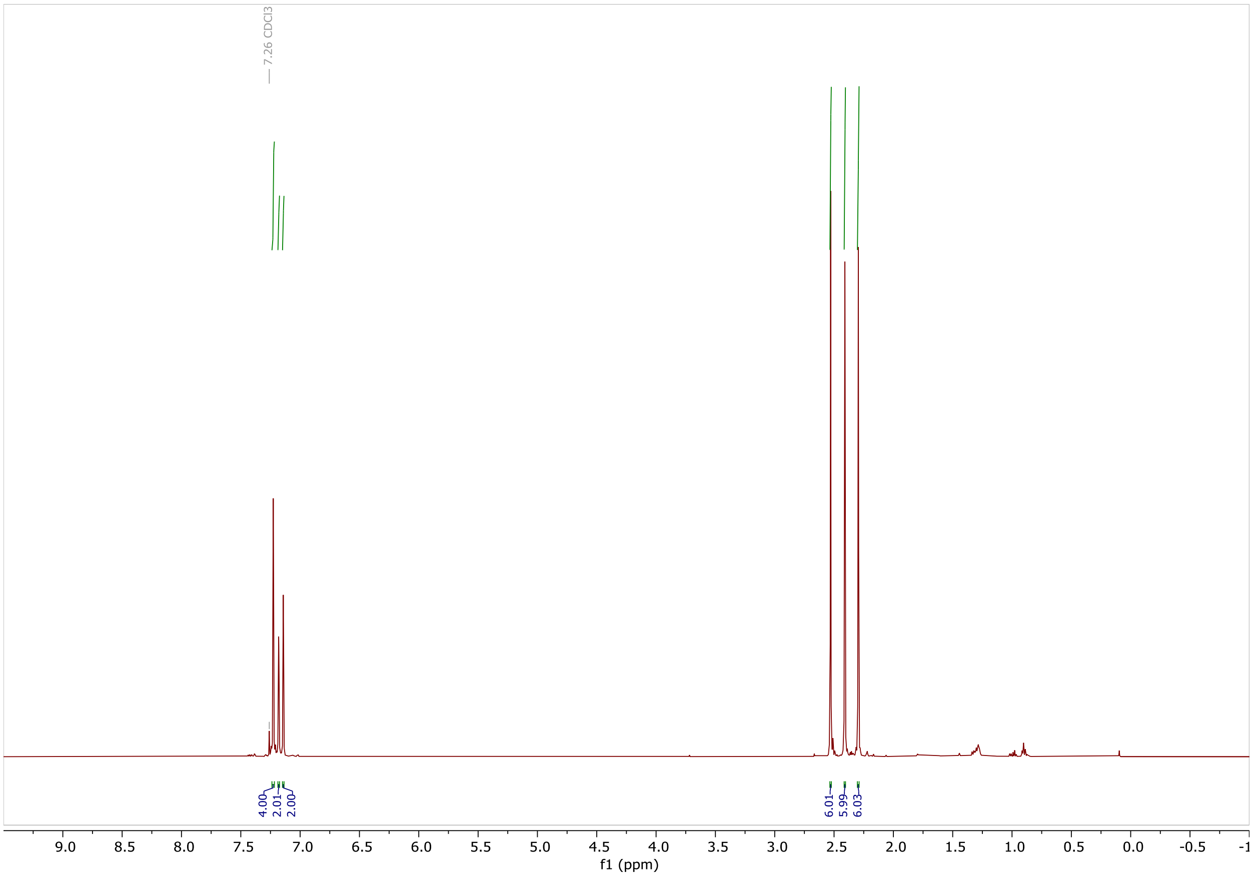

Supplementary Figure 56. ^{1}H-NMR spectrum (500 MHz, $CDCl_3$) for **R1-*m*-Me**.

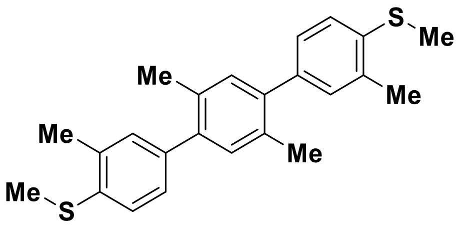

R1-*m*-Me

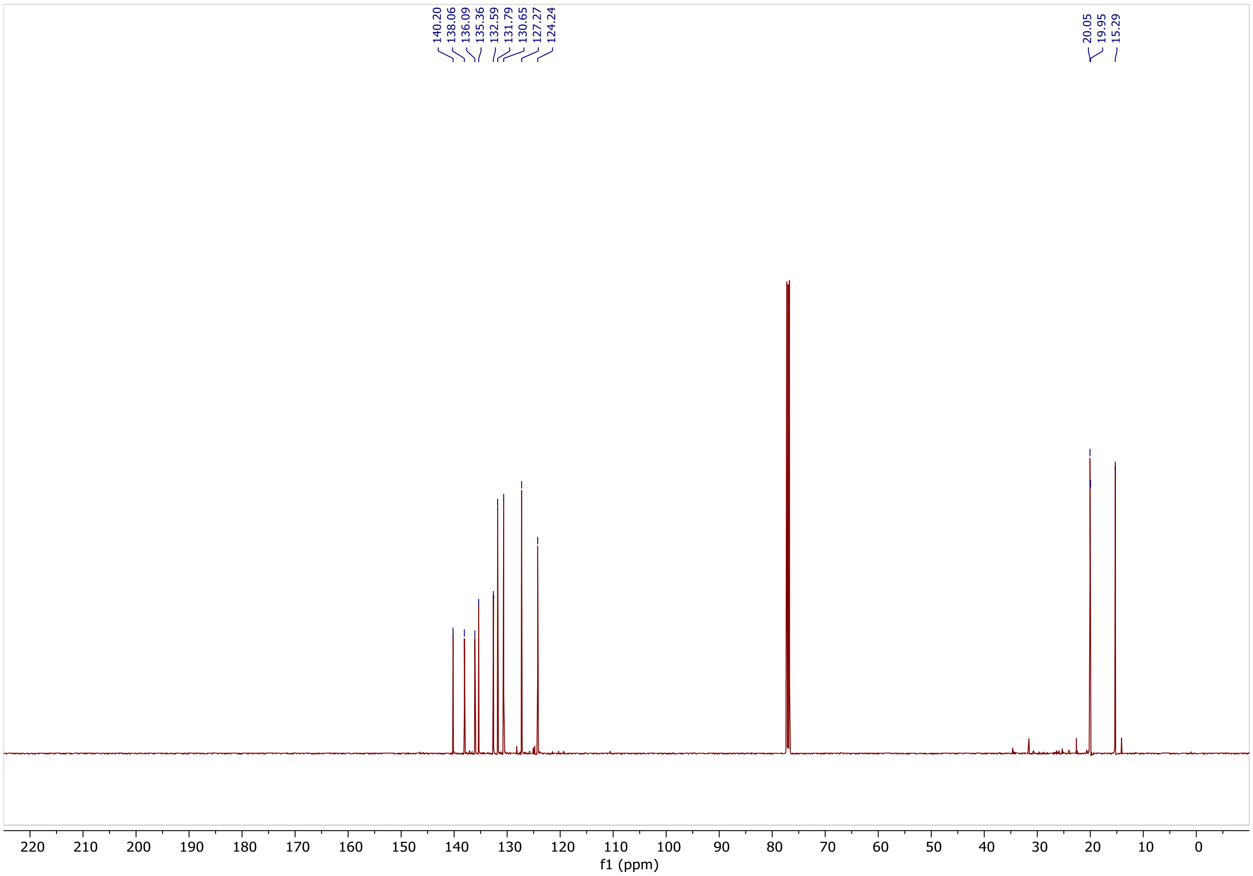

Supplementary Figure 57. ^{13}C-NMR spectrum (126 MHz, $CDCl_3$) for **R1-*m*-Me**.

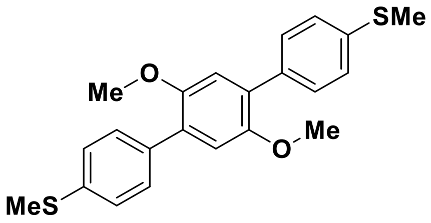

RO1

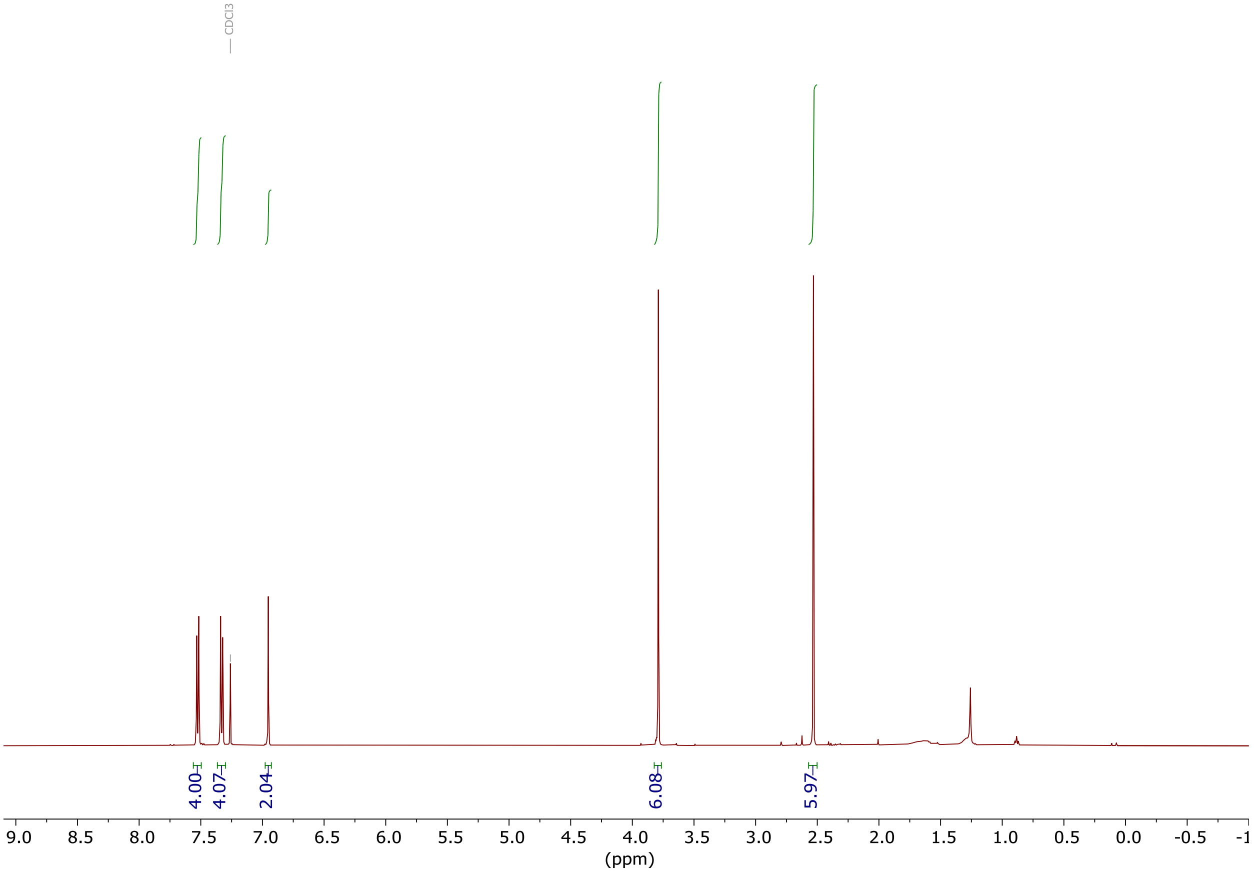

Supplementary Figure 58. ^{1}H-NMR spectrum (500 MHz, $CDCl_3$) for **RO1**.

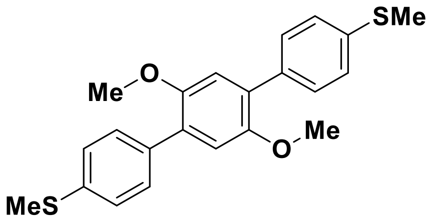

RO1

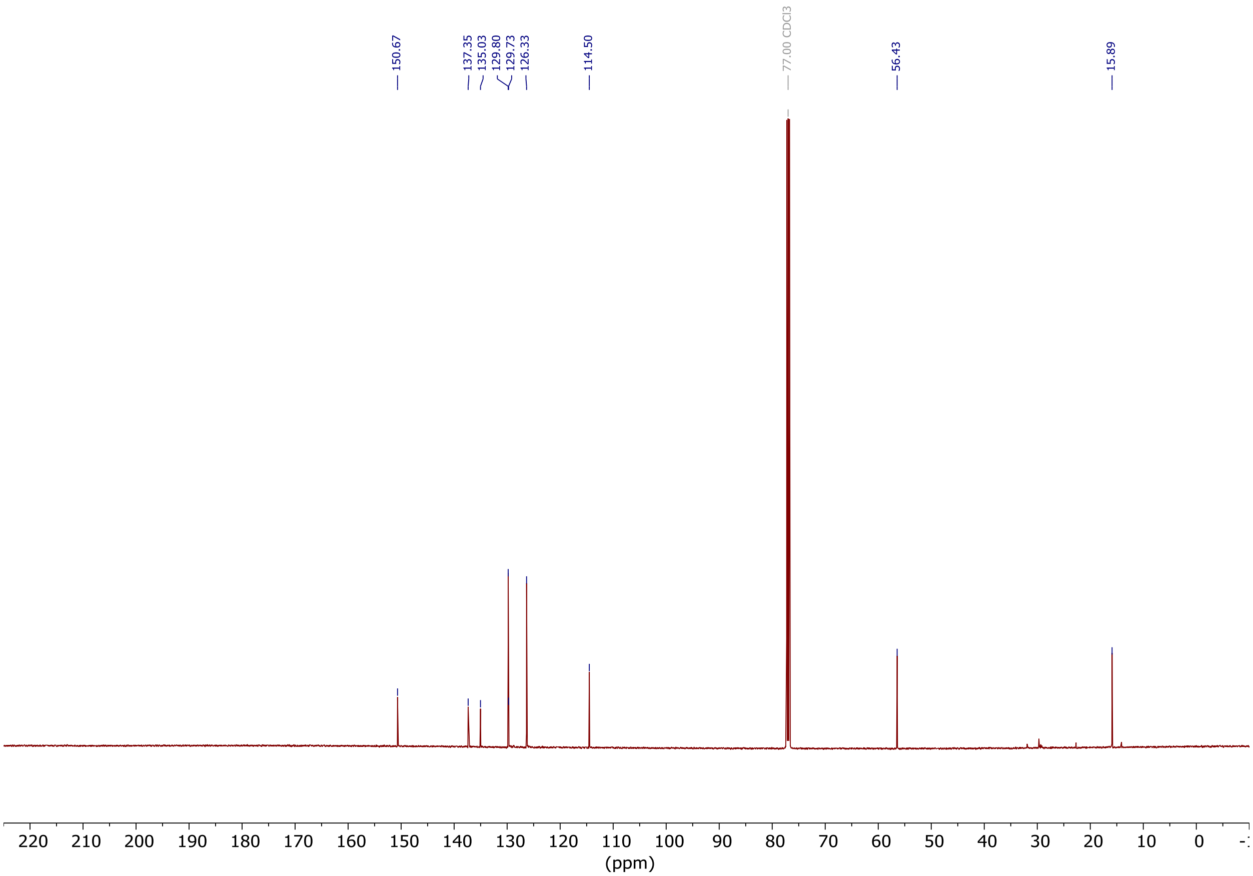

Supplementary Figure 59. ^{13}C-NMR spectrum (126 MHz, $CDCl_3$) for **RO1**.

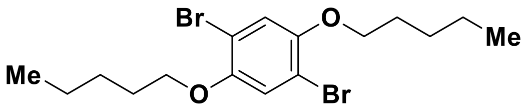

RO5-Br

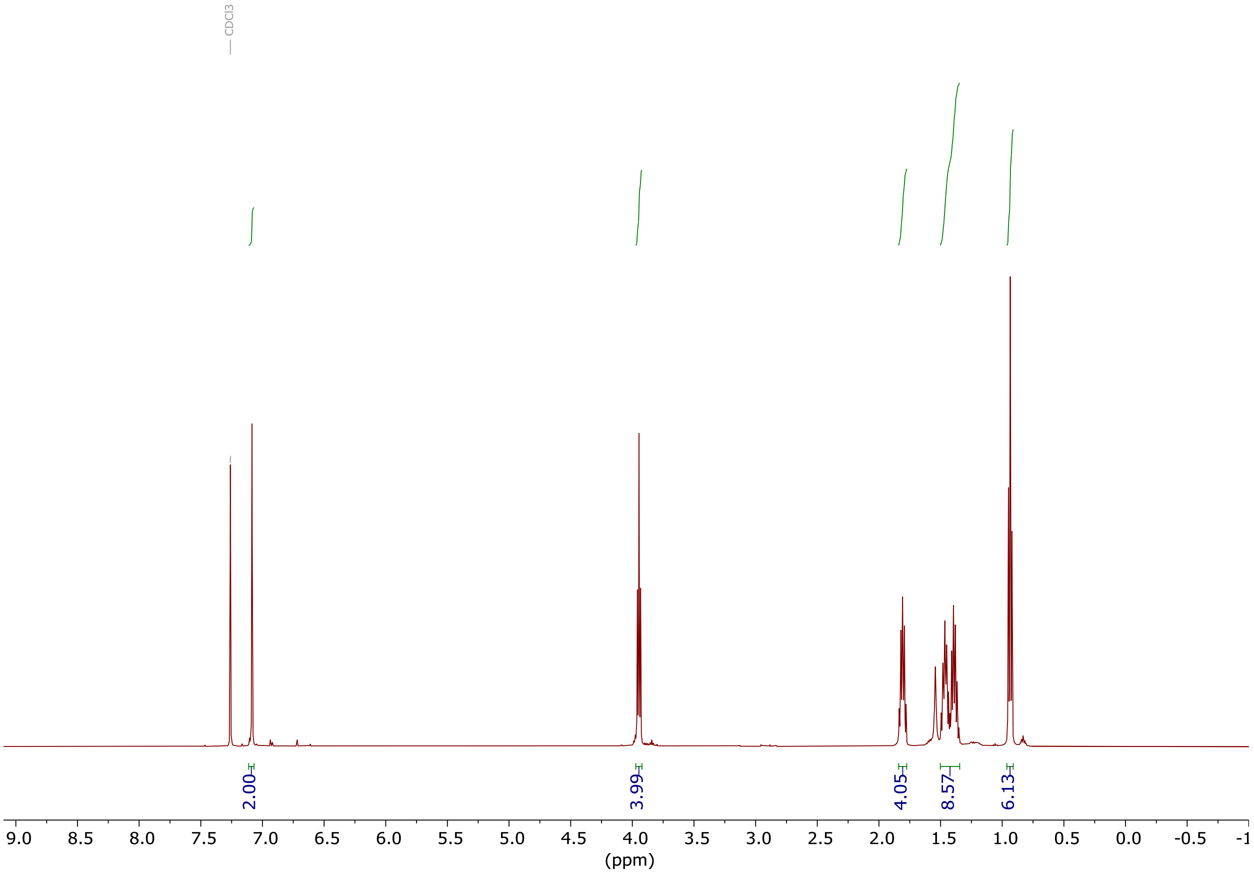

Supplementary Figure 60. ^{1}H-NMR spectrum (500 MHz, $CDCl_3$) for **RO5-Br**.

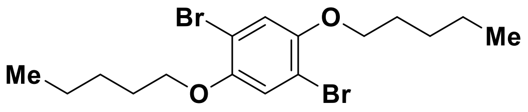

RO5-Br

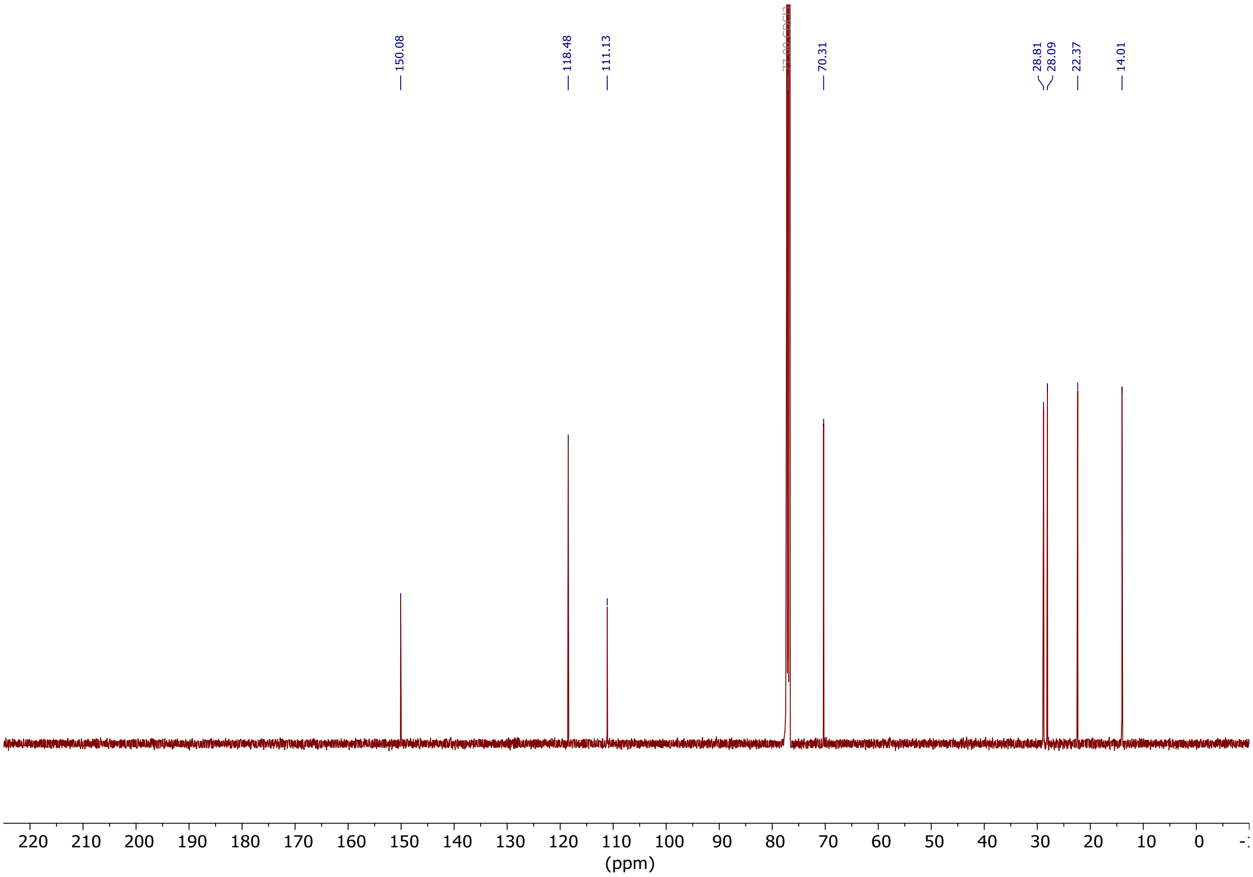

Supplementary Figure 61. ^{13}C-NMR spectrum (126 MHz, $CDCl_3$) for **RO5-Br**.

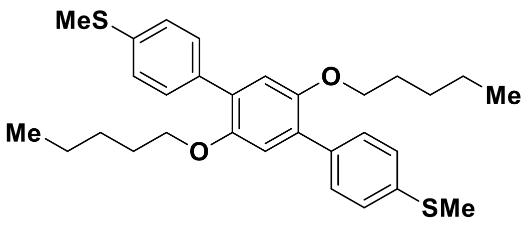

RO5

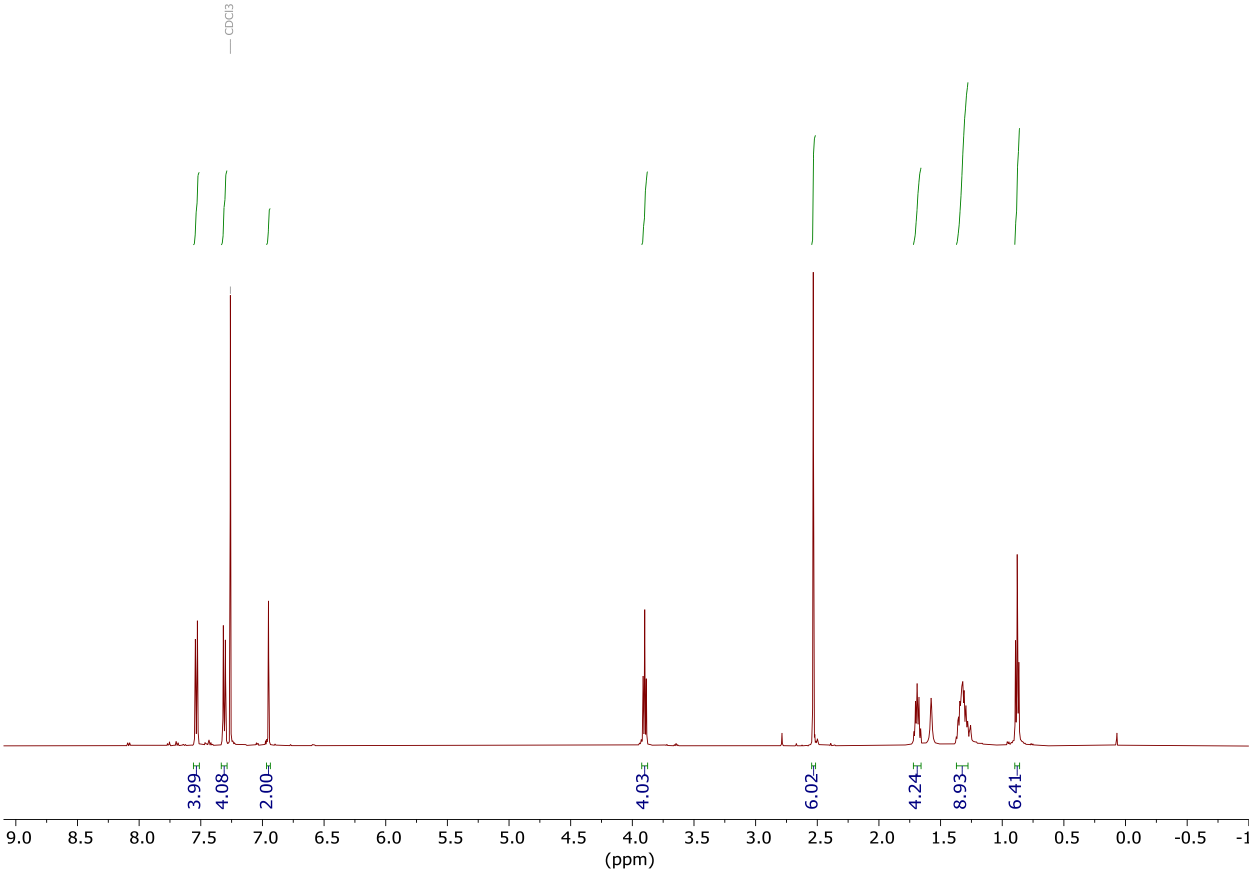

Supplementary Figure 62. ^{1}H-NMR spectrum (500 MHz, $CDCl_3$) for **RO5**.

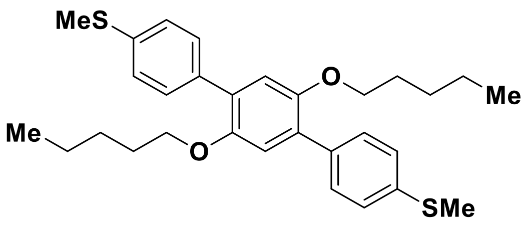

RO5

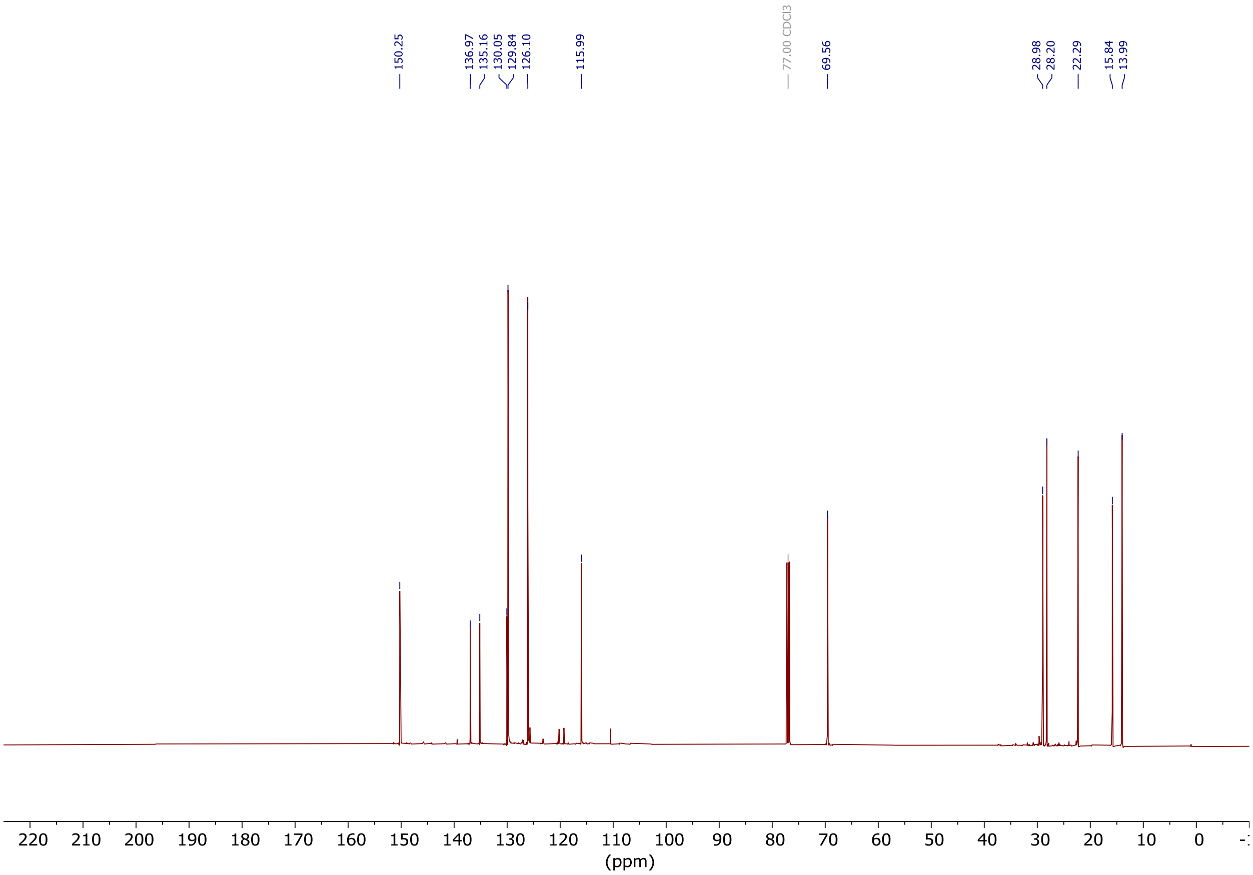

Supplementary Figure 63. ^{13}C-NMR spectrum (126 MHz, $CDCl_3$) for **RO5**.

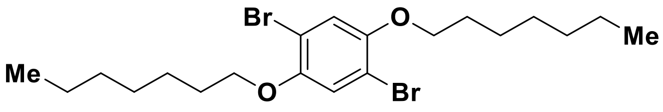

RO7-Br

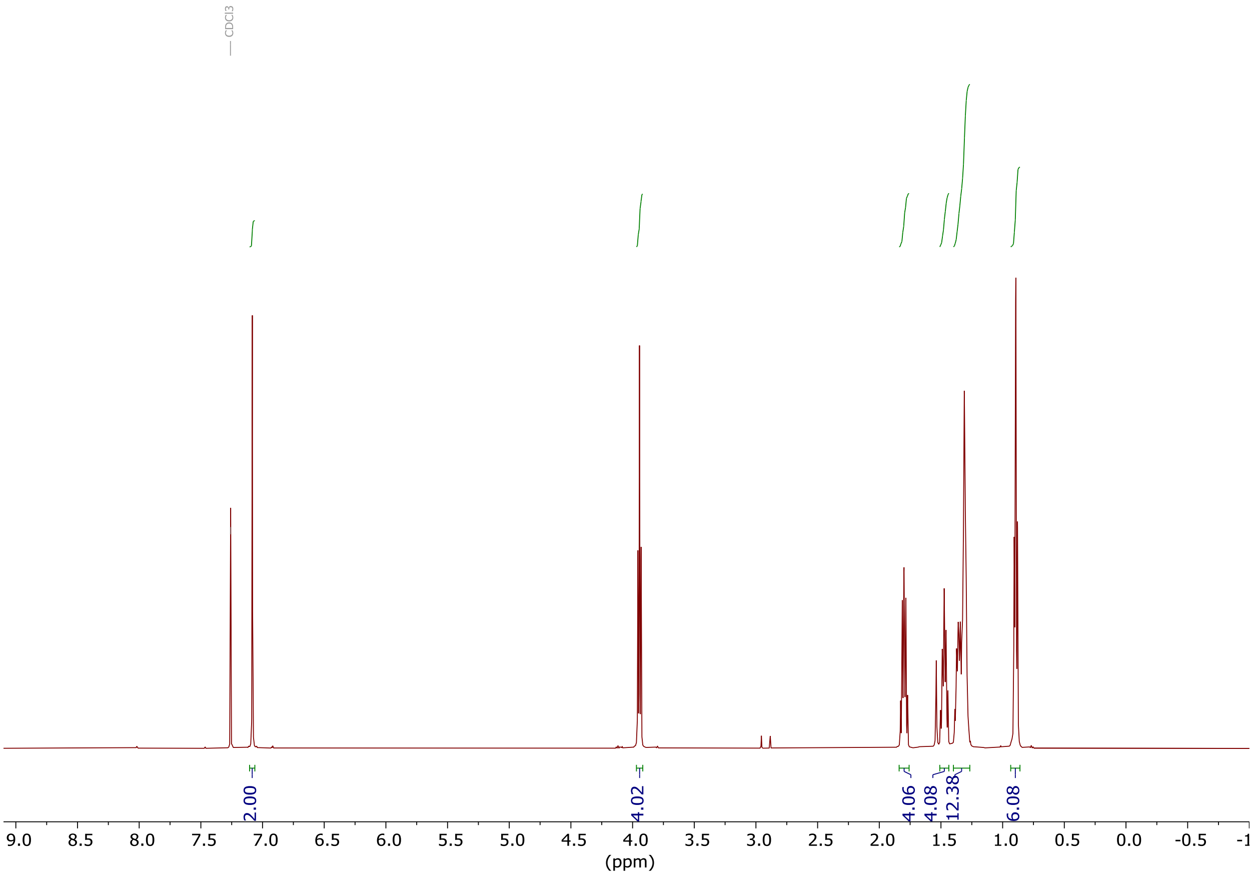

Supplementary Figure 64. ^{1}H-NMR spectrum (500 MHz, $CDCl_3$) for **RO7-Br**.

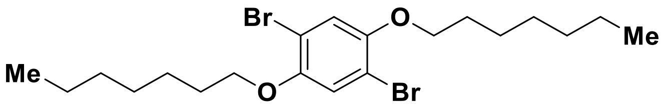

RO7-Br

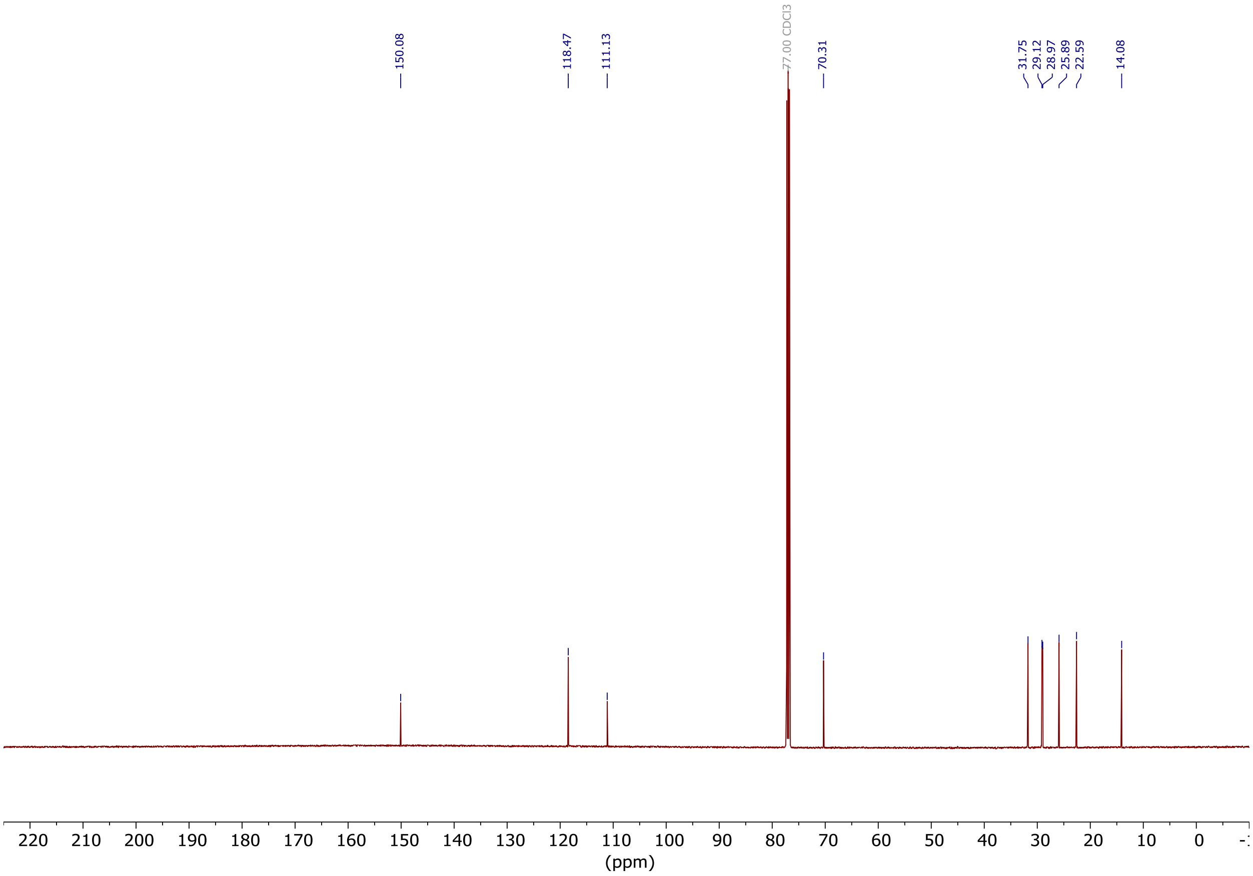

Supplementary Figure 65. ^{13}C-NMR spectrum (126 MHz, $CDCl_3$) for **RO7-Br**.

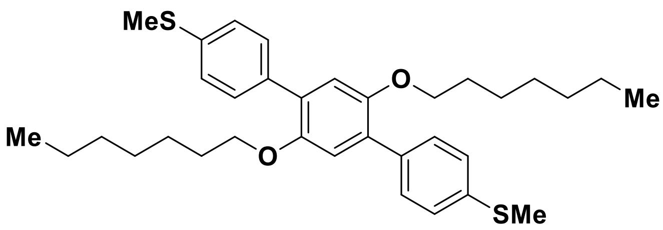

RO7

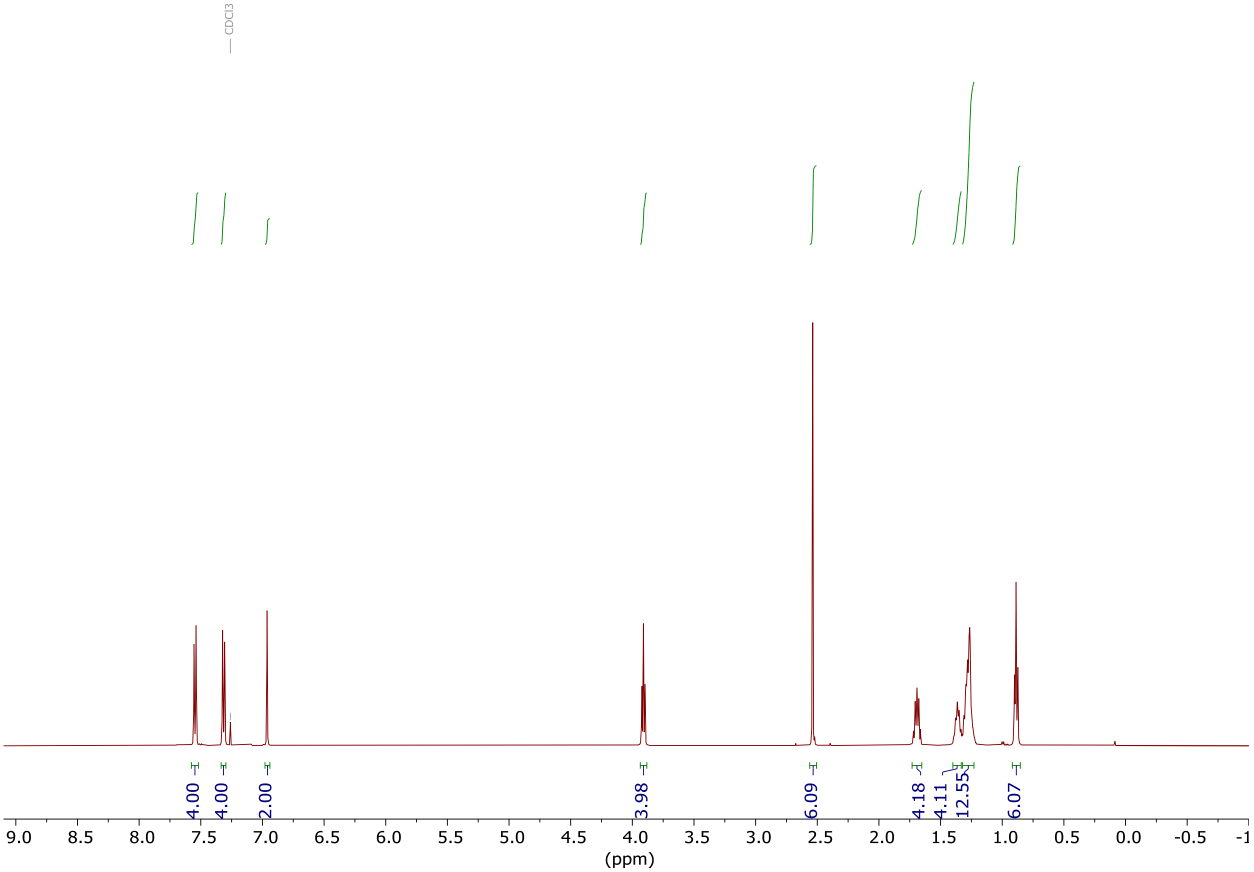

Supplementary Figure 66. ^{1}H-NMR spectrum (500 MHz, $CDCl_3$) for **RO7**.

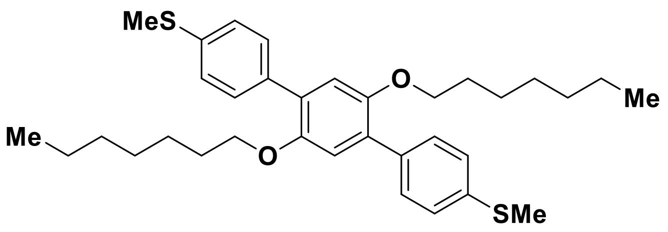

RO7

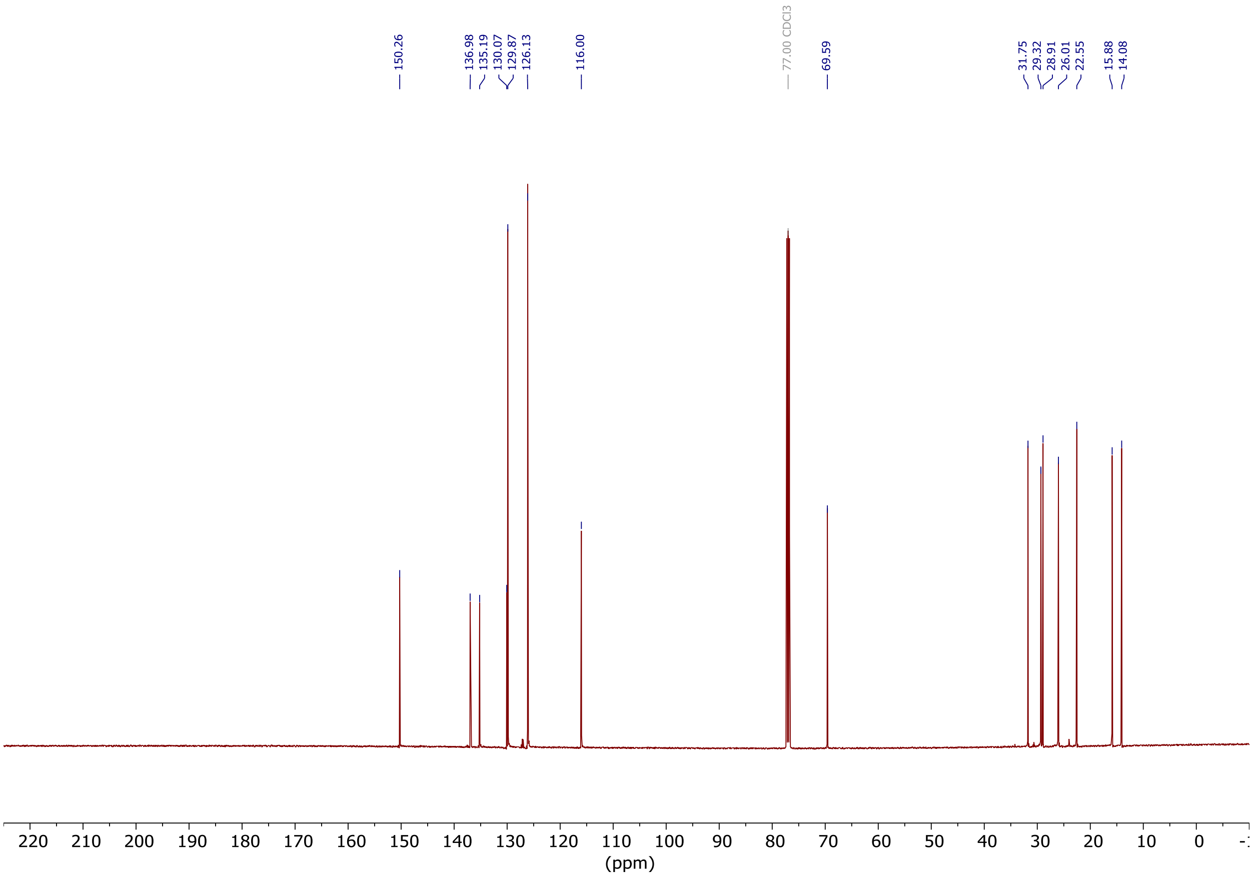

Supplementary Figure 67. ^{13}C-NMR spectrum (126 MHz, $CDCl_3$) for **RO7**.

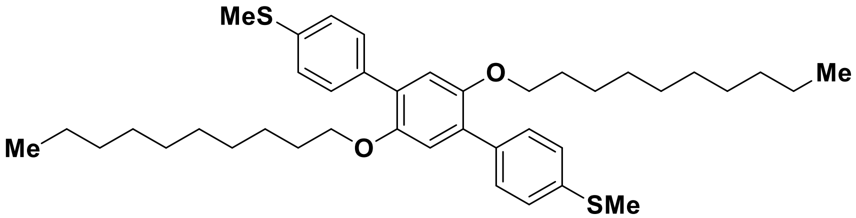

RO10

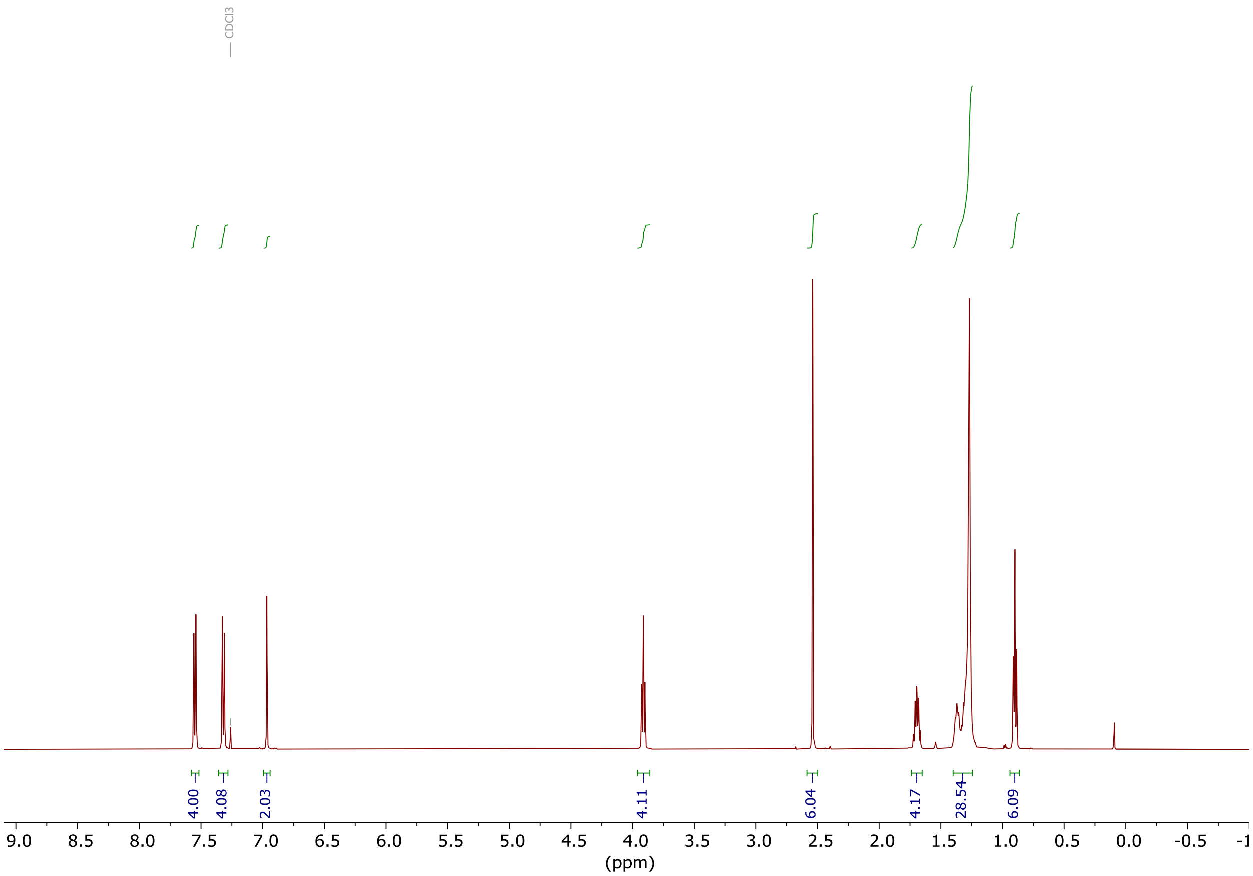

Supplementary Figure 68. ^{1}H-NMR spectrum (500 MHz, $CDCl_3$) for **RO10**.

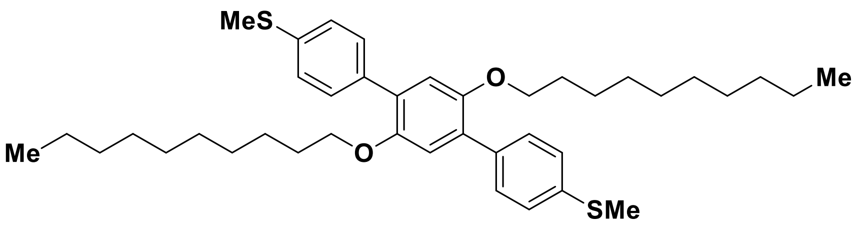

RO10

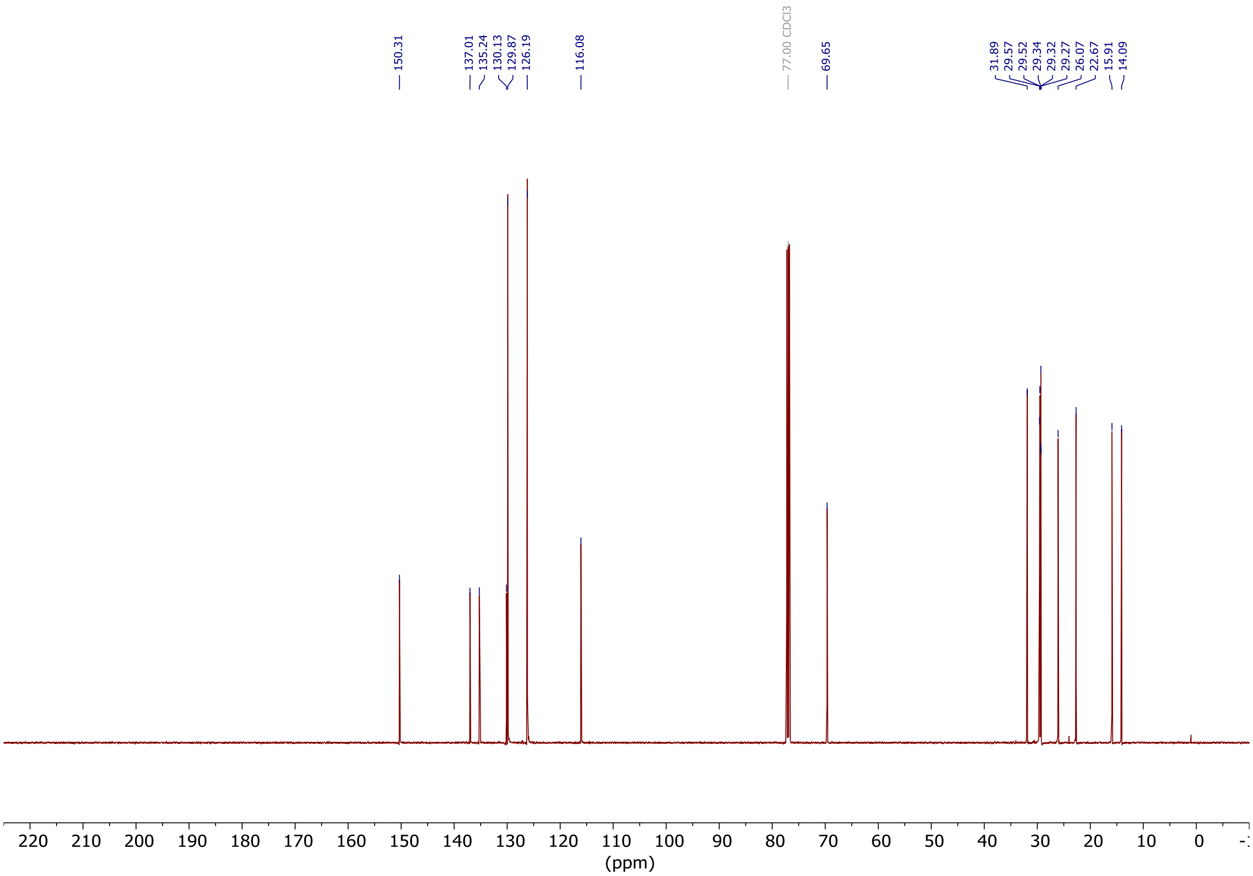

Supplementary Figure 69. ^{13}C-NMR spectrum (126 MHz, $CDCl_3$) for **RO10**.

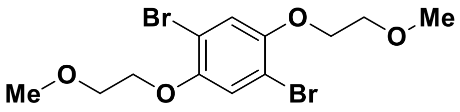

O2-Br

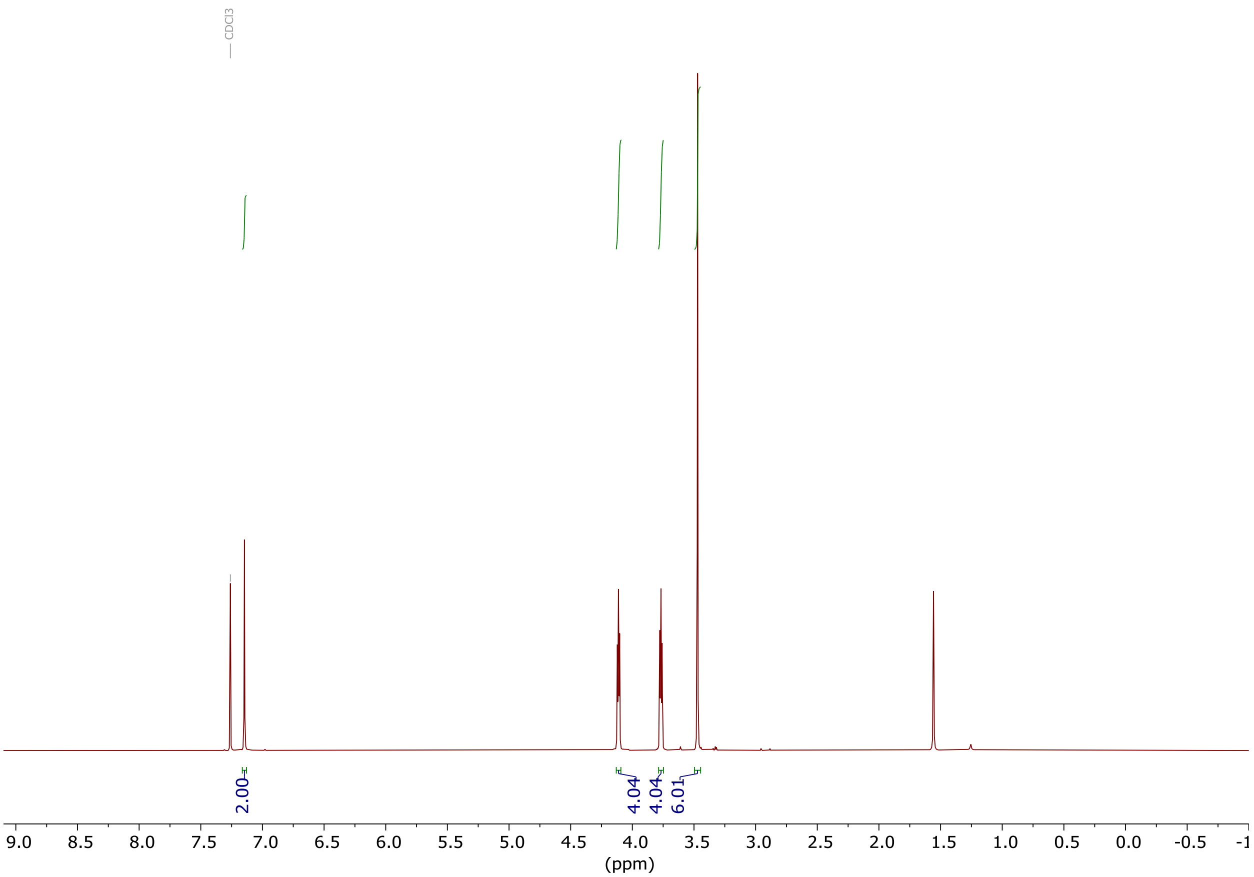

Supplementary Figure 70. ^{1}H-NMR spectrum (500 MHz, $CDCl_3$) for **O2-Br.**

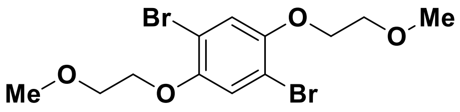

O2-Br

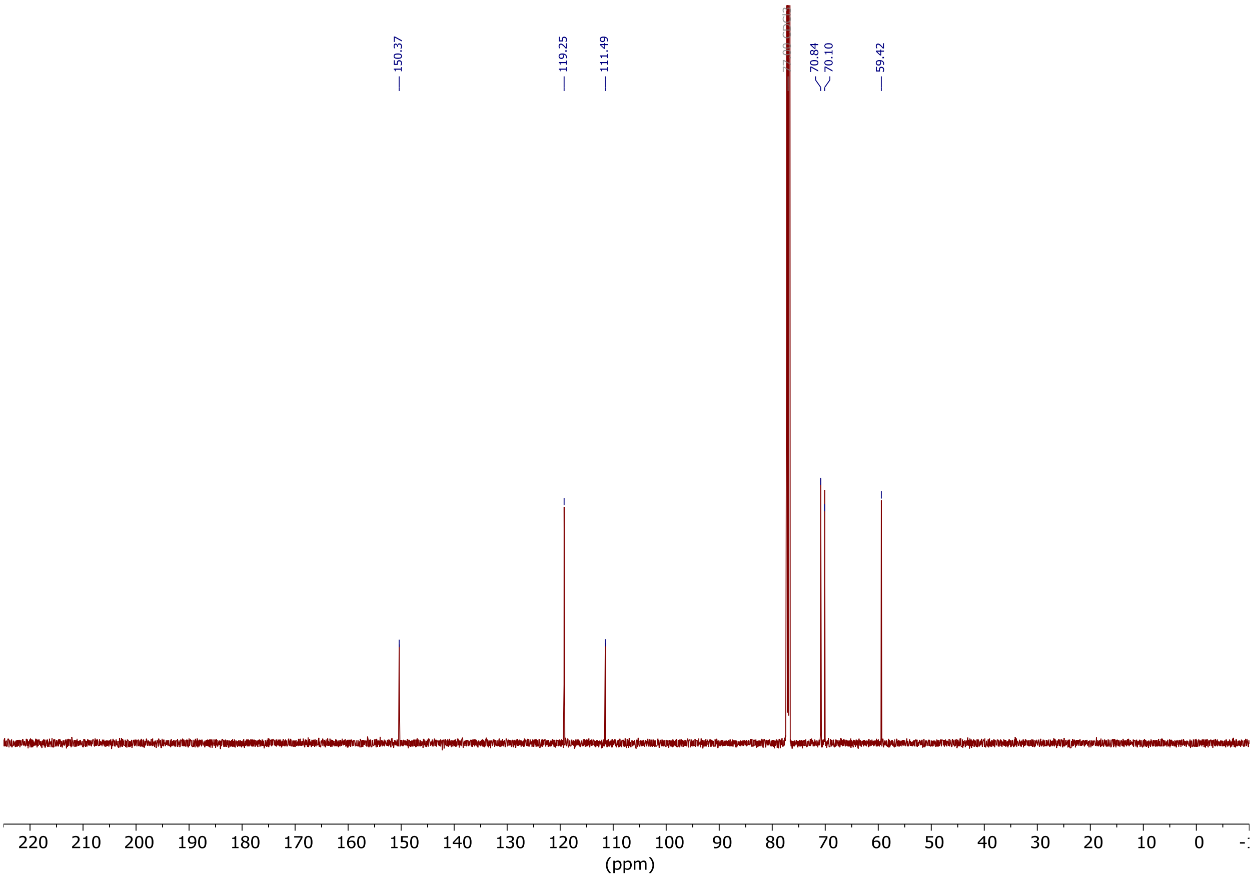

Supplementary Figure 71. ^{13}C-NMR spectrum (126 MHz, $CDCl_3$) for **O2-Br.**

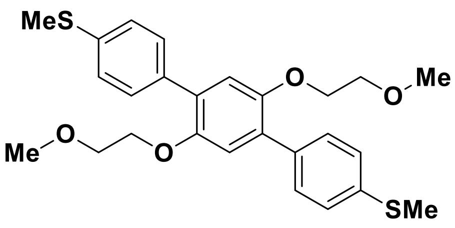

O2

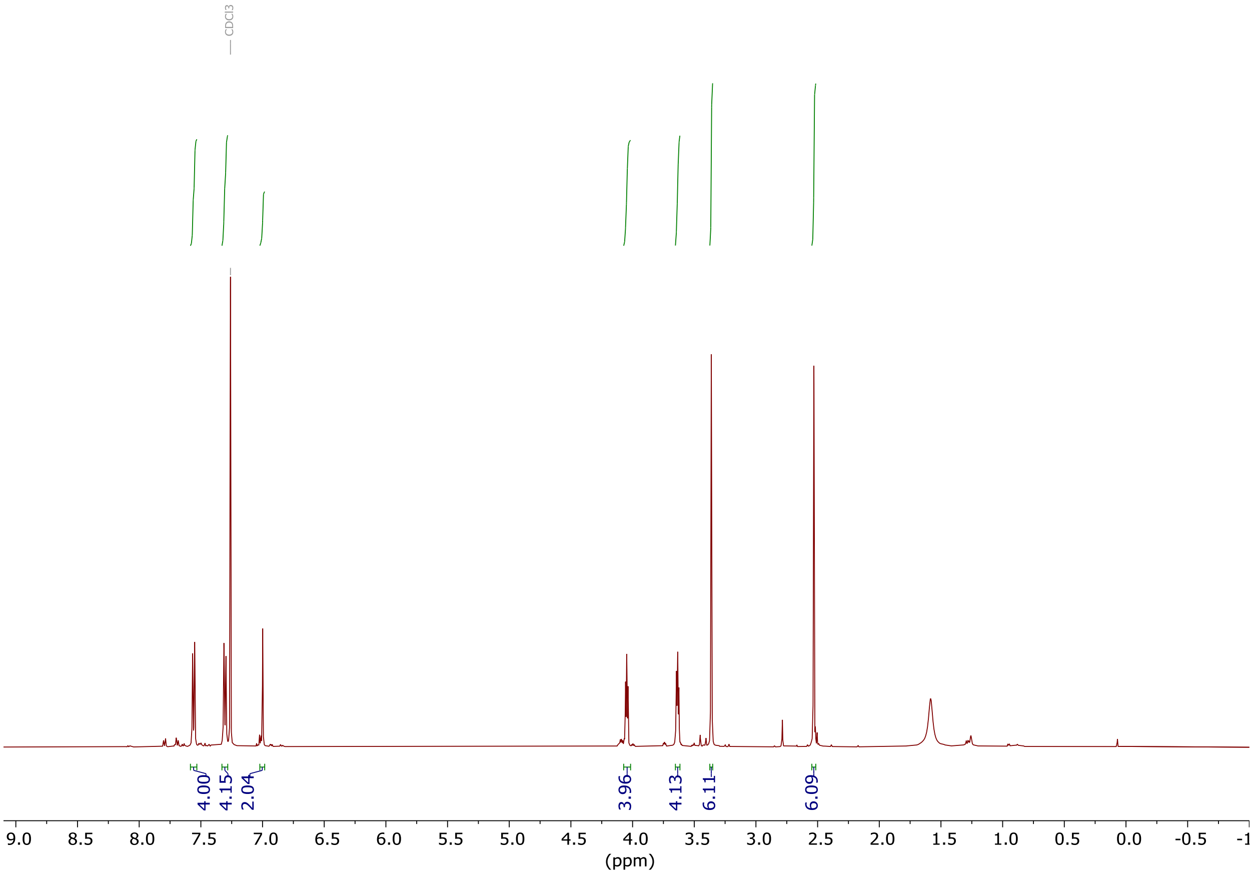

Supplementary Figure 72. ^{1}H-NMR spectrum (500 MHz, $CDCl_3$) for **O2**.

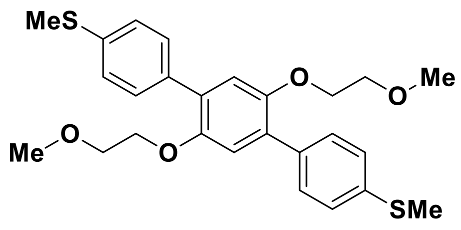

O2

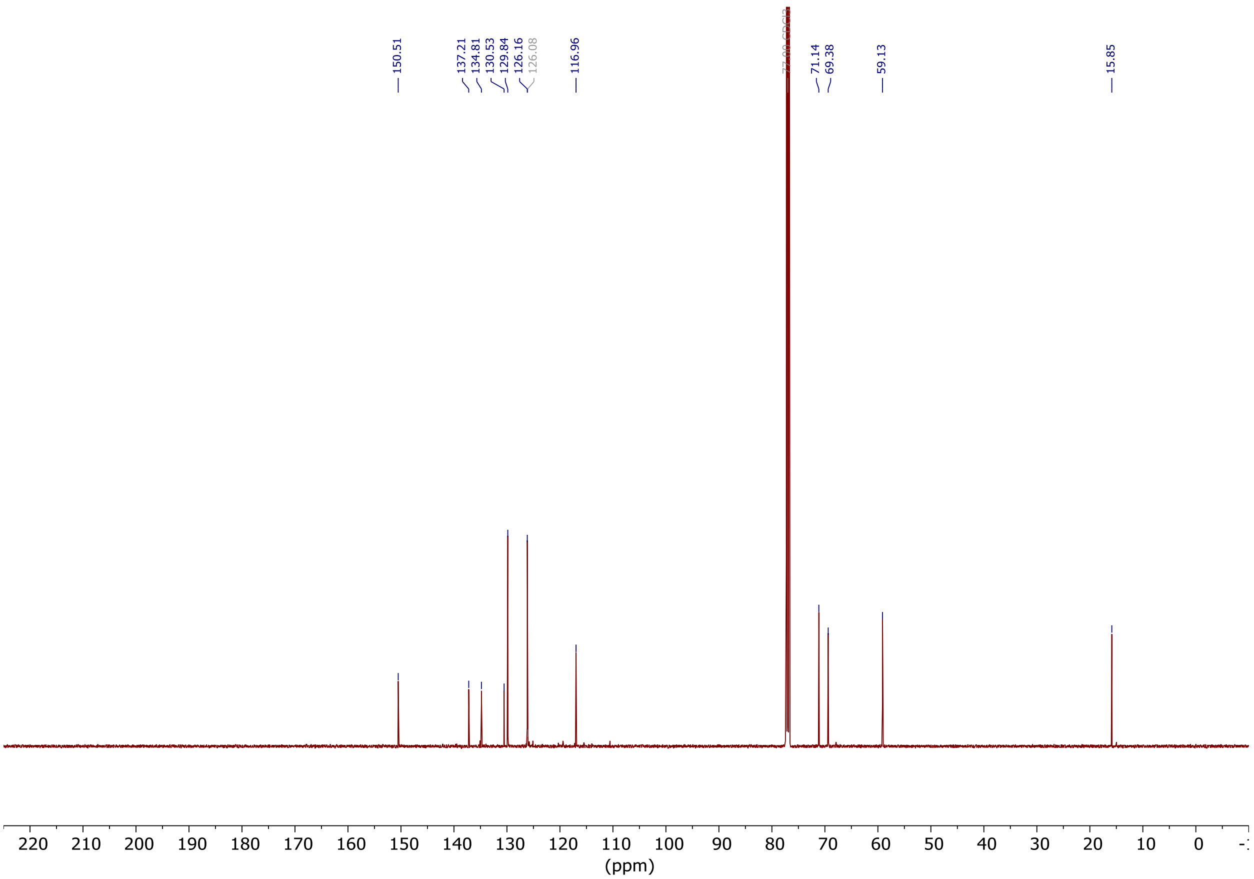

Supplementary Figure 73. ^{13}C-NMR spectrum (126 MHz, $CDCl_3$) for **O2**.

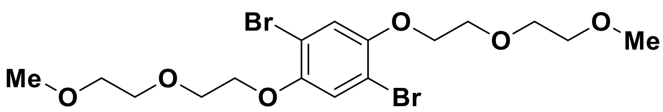

O3-Br

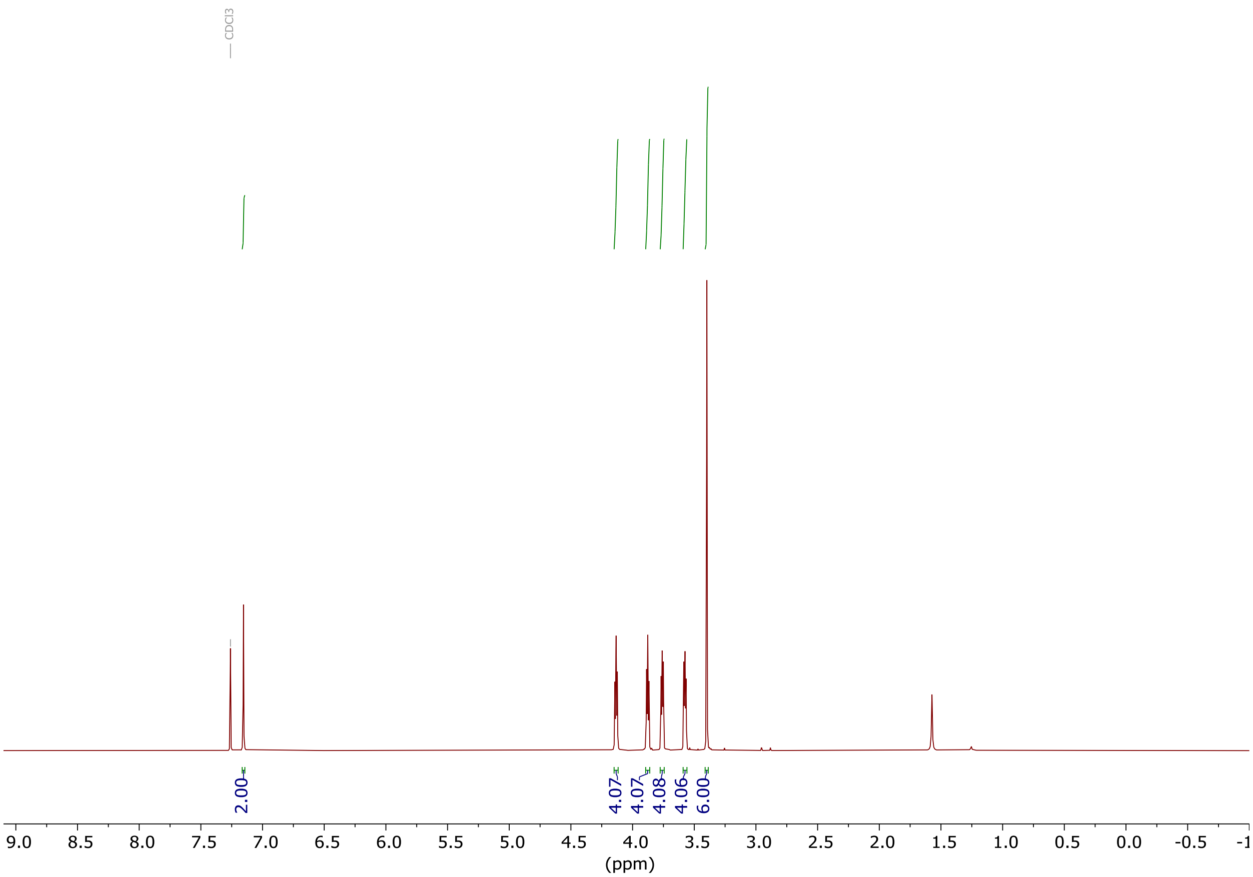

Supplementary Figure 74. ^{1}H-NMR spectrum (500 MHz, $CDCl_3$) for **O3-Br**.

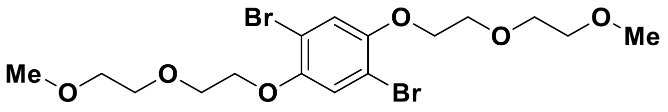

O3-Br

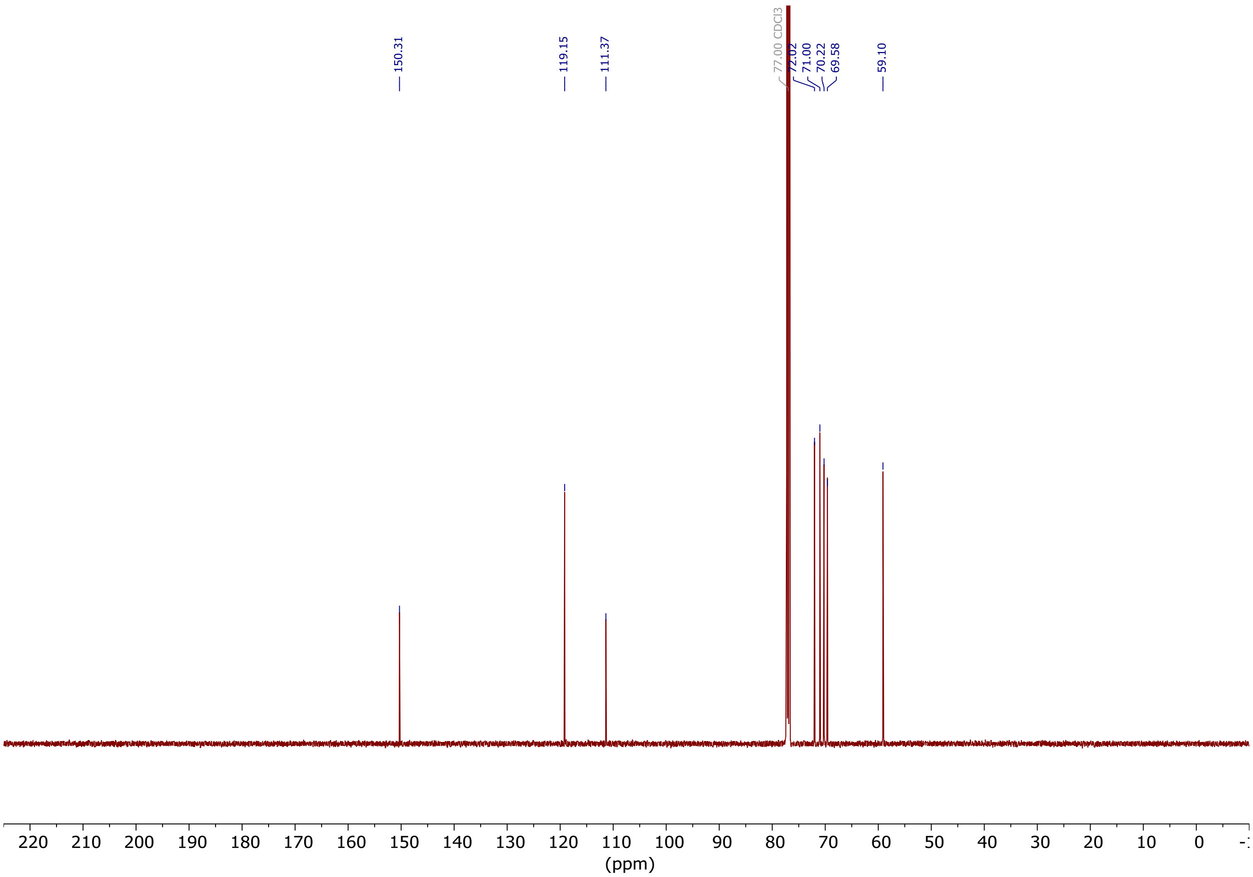

Supplementary Figure 75. ^{13}C-NMR spectrum (126 MHz, $CDCl_3$) for **O3-Br**.

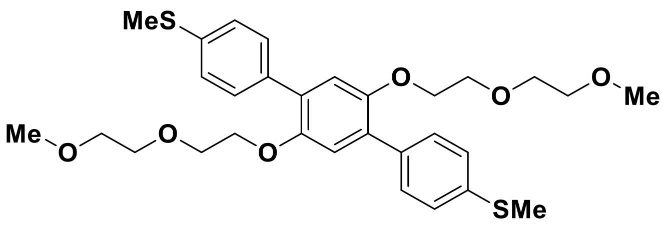

O3

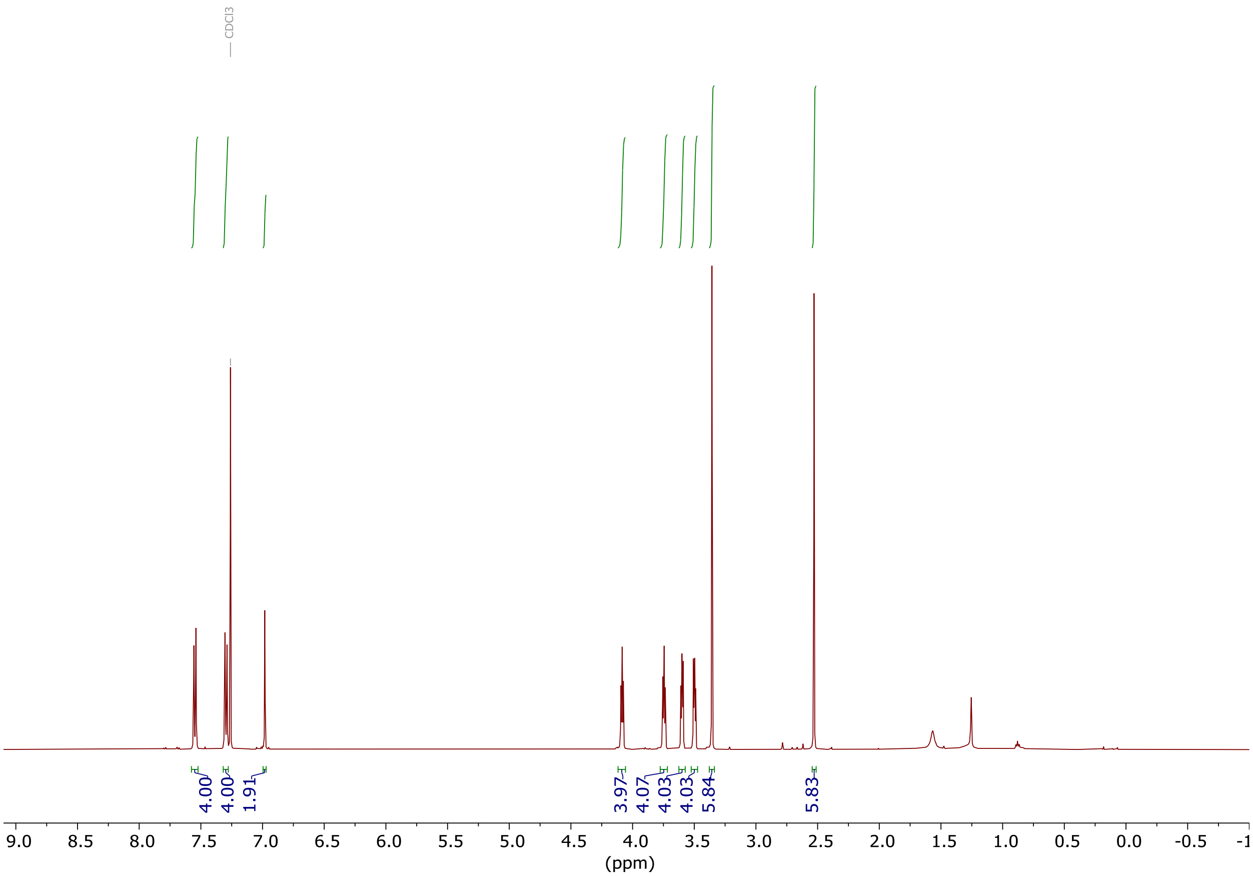

Supplementary Figure 76. ^{1}H-NMR spectrum (500 MHz, $CDCl_3$) for **O3**.

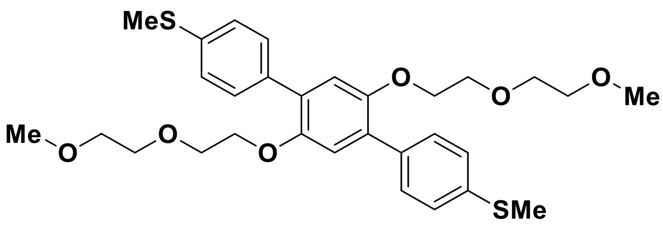

O3

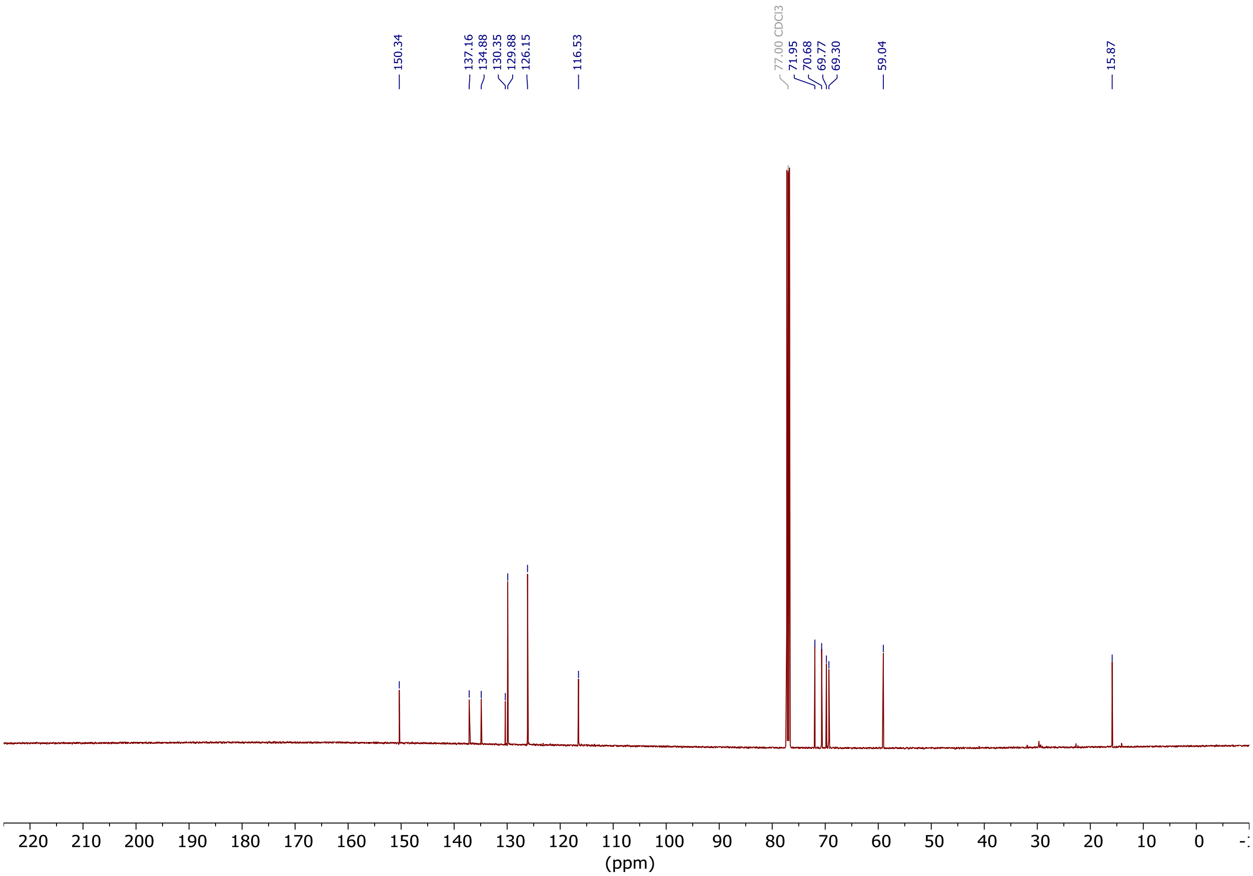

Supplementary Figure 77. ^{13}C-NMR spectrum (126 MHz, $CDCl_3$) for **O3**.

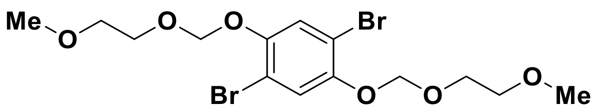

MEM-Br

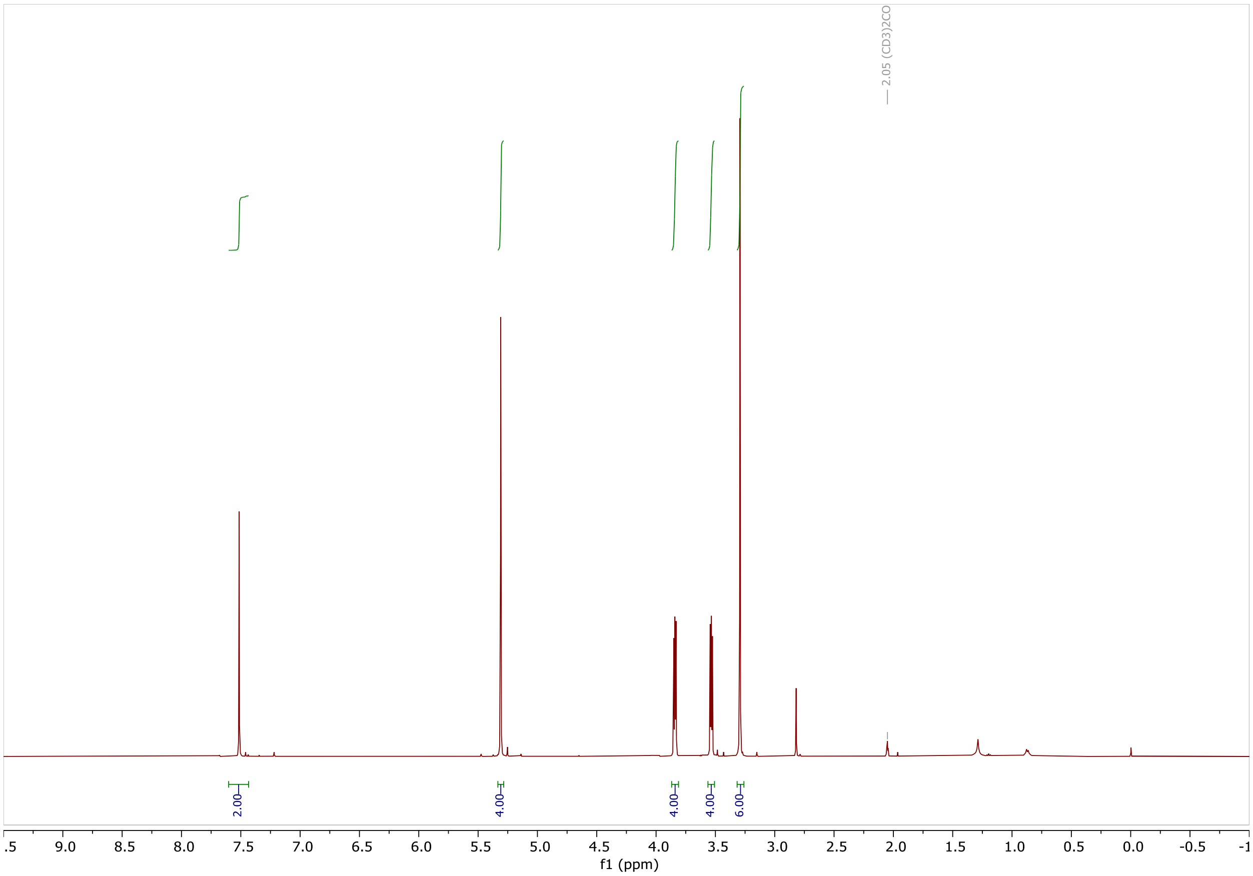

Supplementary Figure 78. ^{1}H-NMR spectrum (500 MHz, $(CD_3)_2CO$) for **MEM-Br**.

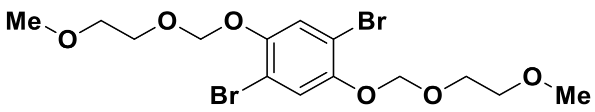

MEM-Br

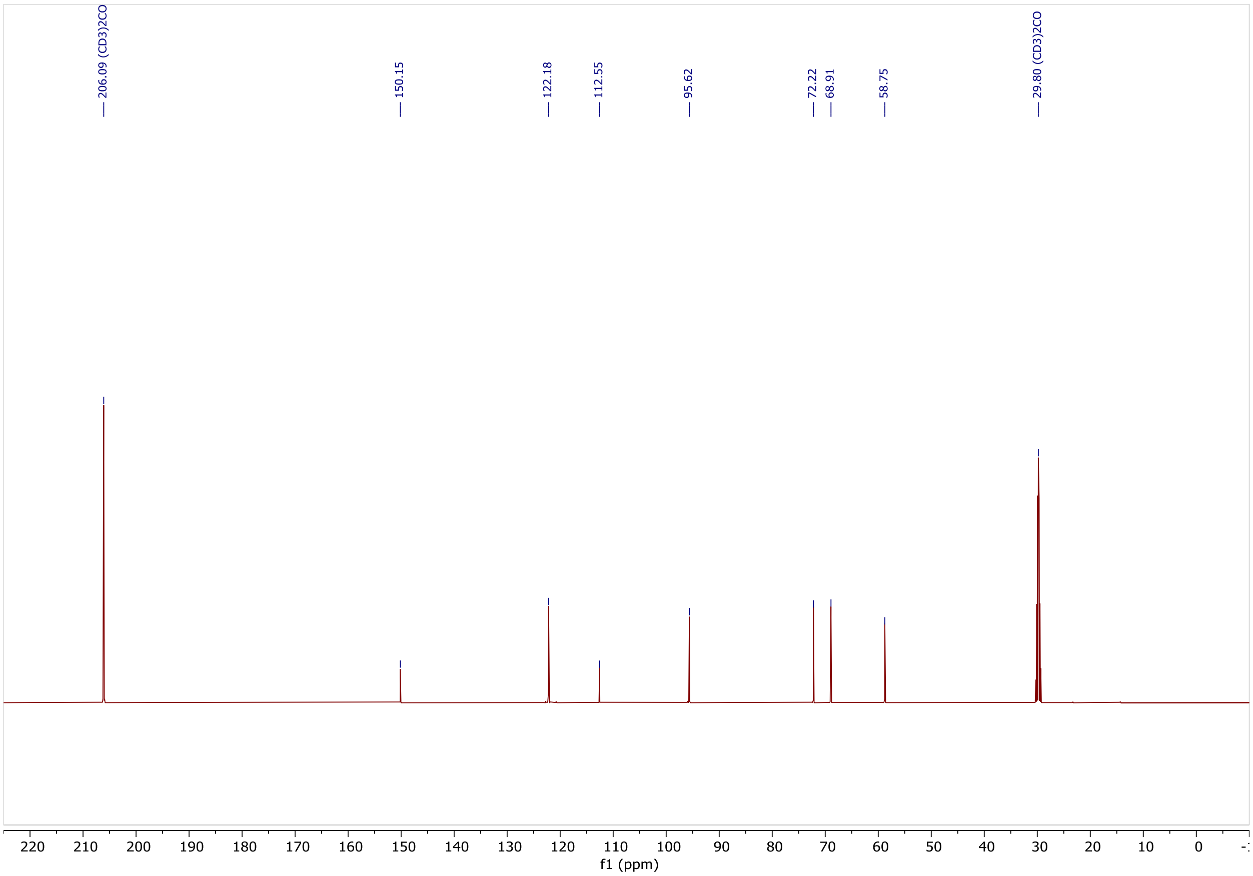

Supplementary Figure 79. ^{13}C-NMR spectrum (126 MHz, $(CD_3)_2CO$) for **MEM-Br**.

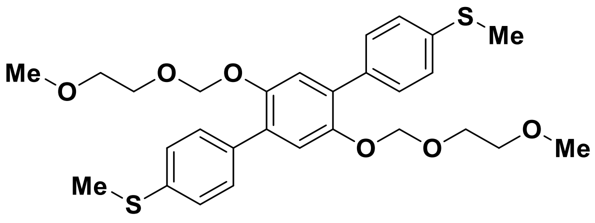

MEM

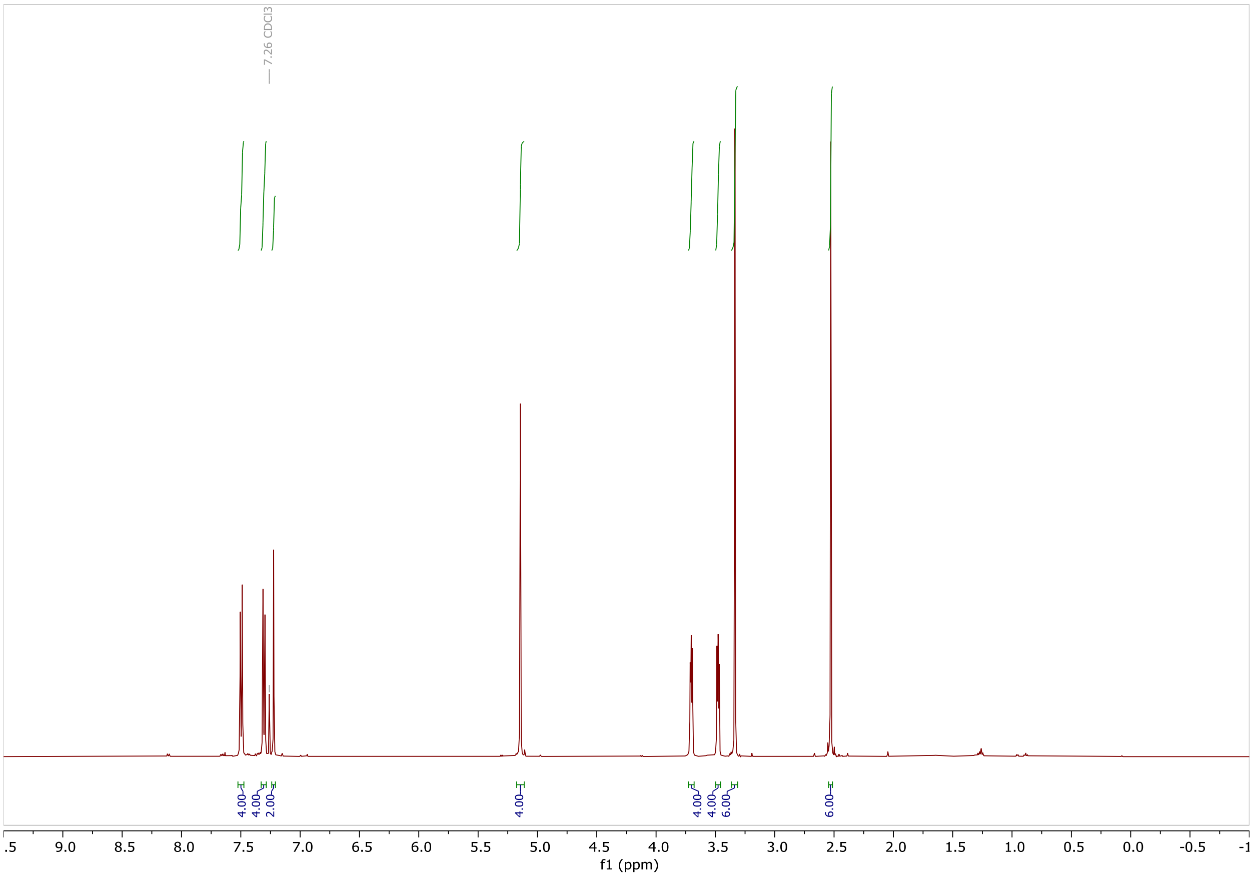

Supplementary Figure 80. ^{1}H-NMR spectrum (500 MHz, $CDCl_3$) for **MEM**.

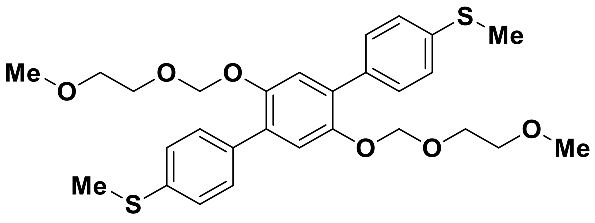

MEM

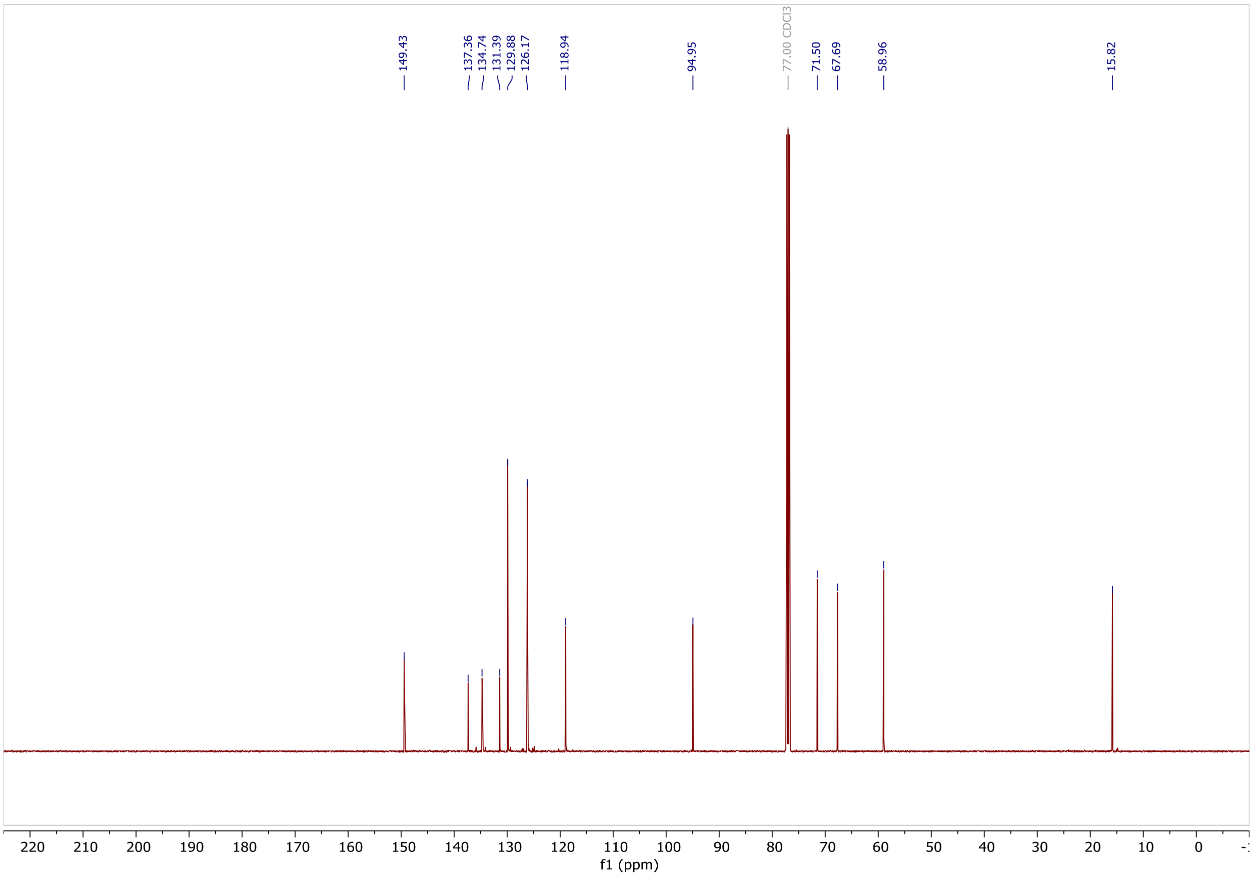

Supplementary Figure 81. ^{13}C-NMR spectrum (126 MHz, $CDCl_3$) for **MEM**.

References

1. Li J, Ballmer SG, Gillis EP, Fujii S, Schmidt MJ, Palazzolo AME*, et al.* Synthesis of many different types of organic small molecules using one automated process. *Science* 2015, **347**(6227)**:** 1221-1226.

2. Pelter A, Jenkins I, Jones DE. The preparations and some properties of mixed aryl-thienyl oligomers and polymers. *Tetrahedron* 1997, **53**(30)**:** 10357-10400.

3. Wessig P, Gerngroß M, Freyse D, Bruhns P, Przezdziak M, Schilde U*, et al.* Molecular Rods Based on Oligo-spiro-thioketals. *The Journal of Organic Chemistry* 2016, **81**(3)**:** 1125-1136.

4. Starr RL, Fu T, Doud EA, Stone I, Roy X, Venkataraman L. Gold–Carbon Contacts from Oxidative Addition of Aryl Iodides. *Journal of the American Chemical Society* 2020, **142**(15)**:** 7128-7133.

5. Hadizad T, Zhang J, Wang ZY, Gorjanc TC, Py C. A General Synthetic Route to Indenofluorene Derivatives as New Organic Semiconductors. *Organic Letters* 2005, **7**(5)**:** 795-797.

6. Zhang H, Kotlear EA, Kushida S, Maier S, Rominger F, Freudenberg J*, et al.* Linear and Star-Shaped Extended Di- and Tristyrylbenzenes: Synthesis, Characterization and Optical Response to Acid and Metal Ions. *Chemistry – A European Journal* 2020, **26**(36)**:** 8137-8143.

7. Snyder SA, Treitler DS. Et2SBr·SbCl5Br: An Effective Reagent for Direct Bromonium-Induced Polyene Cyclizations. *Angewandte Chemie International Edition* 2009, **48**(42)**:** 7899-7903.
